# Supplementary material for: Integration of human organoids single‐cell transcriptomic profiles and human genetics repurposes critical cell type‐specific drug targets for severe COVID‐19
Source: Cell Prolif. 2023 Oct 8;57(3):e13558. doi: 10.1111/cpr.13558 (PMC10905359; doi:10.1111/cpr.13558)
Supplement: Supplementary file 1 — Data S1. Supporting information. [file CPR-57-e13558-s001.docx]

***Supplementary methods***

***RISmed-based published evidence searching***

To validate the identified COVID-19-relevant cell types or drugs, we adopted a widely-used method, RISmed (version 2.3.0)^1^, to conduct a PubMed search for resorting to published supporting evidence with regard to the association between COVID-19 and a particular cell type or drug. The RISmed-based search was based on the keywords as following: COVID-19, the coronavirus disease 2019, SARS-COV-2, and specific cell type or drug. Through counting the number of reported studies using the keywords pairs, we performed the Pearson correlation analysis^2^ to calculate the correlation coefficients between the number publications (log2(n+1)-transformed) and the significant percent of each cell type detected by the scPagwas (version 1.1.0) method^3^. For COVID-19-relevant drugs, we calculated the proportion of drugs with reported supporting evidence among all identified drugs.

***Protein-protein interaction network analysis***

Based on the S-MultiXcan-based transcriptome-wide association analysis, we found that 67 common genes were significantly associated with COVID-19 initiation and progression. In view of previous consensus support that disease-causing genes are more likely to show high inter-communications^4-10^, thus, we used these common genes as input to conduct a protein-protein interaction (PPI) network analysis using the STRING database (version 11.5, <https://string-db.org/>) ^11^, a comprehensive database concentrating on the networks and interactions of proteins in a wide array of species. The STRING network clusters are pre-computed protein clusters derived by hierarchically clustering the full STRING network using an average linkage algorithm. The significance of the network represents the input genes have more interactions among themselves than what would be expected for a random set of genes of the same size and degree distribution drawn from the genome. Based on the STRING database, our results showed these 67 common genes constructed a significant interacted network than random genes, suggesting that these genes as a group are at least partially biologically connected.

***Calculation of per-cell genetic risk scores of 438 COVID-19-relevant genes***

To independently evaluate the genetic relevance of each cell with COVID-19 severity, we adopted a set of 438 COVID-19-relevant genes (i.e., pre-curated gene set) identified from the S-MultiXcan-based transcriptome-wide association analysis^12^ to calculate the genetic risk score for each cell. For given cell *c* and a pre-collected gene set *g*, the raw per-cell genetic risk score GRS*_g_* (*c*) is initially calculated based on the average relative expression of genes from the predefined gene set in the respective cell. However, such raw score may be confounded by the complexity of cells, as cells with higher complexity prone to have more genes identified that lead to achieve higher per-cell genetic risk scores for any expression gene set. To reduce the influence of these confounding factors, we add a control gene set to calculate a control cell score CCS*_g_*(*c*), and subtract it from the raw per-cell genetic risk score to yield final genetic risk score for each cell: $\bar{GRSg}=GRSg-CCSg$. The control gene set is randomly chosen based on the aggregate expression level bins, which have a comparable distribution of expression levels and over size to that of the pre-defined gene set. The *AddModuleScore()* function in Seurat (Version 4.3.0)^13^ R package is utilized to perform this calculation with default parameter.

***Calculation of molecular signature scores of fibroblast marker genes***

As reference to a recent study ^14^, we employed the molecular signature scores of fibroblast marker genes (denoted as fibroblast cell state scores) to compare the similarity between lung MSC positive cells and fibroblasts. The fibroblast cell state scores are calculated based on the average relative expression of the genes from a short-list of common fibroblast markers ^15^, including many extracellular matrix genes (ECM), i.e., *COL1A1*, *COL1A2*, *COL5A1*, *LOXL1*, *LUM*, *FBLN1*, and *FBLN2*, and the cell surface receptors, i.e., *CD34* and *PDGFRA*, in the respective cell. Analogue to the above method used for calculating the per-cell genetic risk score, the *AddModuleScore()* function in Seurat (Version 4.3.0) ^13^ R package is used to perform this calculation with default parameter. The Pearson correlation method is applied to calculate the correlation coefficients between fibroblast cell state scores and scPagwas TRSs across lung MSCs, and the two-sided Wilcoxon sum-rank test ^16^ is leveraged to assess the difference of fibroblast cell state scores between MSC positive cells and negative cells.

***CytoTRACE analysis***

To evaluate the stemness of cells in different clusters of MSCs, we applied the CytoTRACE (v0.3.3) ^17^ to estimate the transcriptional diversity of each cell in MSC clusters in terms of differential statues. CytoTRACE assigns a stemness score ranging from 0 to 1 to each cell according to their differential potential. The higher score indicates higher stemness and less differentiation, and the lower score indicates the lower stemness and more differentiation.

***Developmental trajectory analysis***

To uncover the developmental trajectory of positive cells in MSCs, we used the Monocle2 (v2.28.0) ^18^ to calculate the pseudotime of each cell in clusters of MSCs. The standard protocol of Monocle2 with default settings was leveraged to order cells into potentially differentiated trajectories, and we also used the Wilcoxon sum-rank test to identify genes that alterations in expression over trajectories. The clusterProfiler ^19^ R package was used to perform GO-term enrichment analysis for these branch-dependent genes.

***scDRS analysis for cell scoring in simulated and real scRNA-seq data***

The scDRS (Version 1.0.3) ^20^ method was developed to incorporate scRNA-seq data with GWAS summary statistics for discerning trait-relevant cell subpopulations/clusters at a fine-grain resolution, which show excess expression levels of genes in a given gene set identified from GWAS summary statistics on traits of interest. First, scDRS leverages top 1,000 putative diseases genes identified from GWAS data using gene-based association test methods, such as MAGMA as a default tool^21^. In light of the recent study^3^ has suggested that scPagwas-identified trait-relevant genes could considerably enhance the power of scRDS for inferring trait-relevant cells than that using MAMGA-identified genes, thus, we adopted the top-ranked 1,000 genes from scPagwas as putative disease genes for scDRS-based cell scoring. Then, scDRS quantifies the aggregate expression of genes in the putative disease gene set in each cell to yield cell-specific raw disease scores (*S_True_*). To evaluate the statistical significance for individual cells, scDRS constructs 1,000 sets of control genes by randomly selecting from background genes. These sets of control genes match the mean expression and expression variance of the disease-relevant genes, and are applied for generating 1,000 sets of cell-specific raw control scores (*S_Ctr_*) based on the Monte Carlo (MC) sampling method. After correcting the control gene sets, the cell-specific gold-standard MC P-values are computing using the following equation:

$$P_{c}^{MC}= \frac{1+\sum_{g=1}^{G} (S_{True}\leq S_{Ctr})}{1+G}$$

where *c* is the total number of cells, *G* is the number of selected control gene sets (default 1,000), and $g\in G$. Finally, scDRS provides the estimates of the ideal MC p-values by pooling control scores across all cells. For more detailed information of the scDRS method, please refers to the original paper^20^.

***Network-based analysis for 33 druggable genes***

To investigate the underlying interactions among these identified 33 druggable genes, we performed a network-based enrichment analysis by using the GeneMANIA software (Version 3.5.2, <http://www.genemania.org>)^22^. Through employing a guilt-by-association method to query published genomics and proteomics data, including physical interactions, co-expression links, pathway-based links, shared protein domains, co-localization links, genetic interactions, and predicted links, the GeneMANIA tool could predict putative genes with the similar biological and molecular functions with high efficiency. The Cytoscape platform (Version 3.9.1, <https://cytoscape.org/>)^23^ was used to visualize the network among these 33 druggable genes.

**References**

1. Wang R, Lin D-Y, Jiang Y. EPIC: Inferring relevant cell types for complex traits by integrating genome-wide association studies and single-cell RNA sequencing. *PLoS genetics.* 2022;18(6):e1010251.

2. Cohen I, Huang Y, Chen J, et al. Pearson correlation coefficient. *Noise reduction in speech processing.* 2009:1-4.

3. Ma Y, Deng C, Zhou Y, et al. Polygenic regression uncovers trait-relevant cellular contexts through pathway activation transformation of single-cell RNA sequencing data. *medRxiv.* 2023:2023.2003.2004.23286805.

4. Wang Q, Chen R, Cheng F, et al. A Bayesian framework that integrates multi-omics data and gene networks predicts risk genes from schizophrenia GWAS data. *Nature neuroscience.* 2019;22(5):691-699.

5. Ma Y, Li MD. Establishment of a strong link between smoking and cancer pathogenesis through DNA methylation analysis. *Scientific reports.* 2017;7(1):1-13.

6. Ma X, Wang P, Xu G, Yu F, Ma Y. Integrative genomics analysis of various omics data and networks identify risk genes and variants vulnerable to childhood-onset asthma. *BMC Medical Genomics.* 2020;13:1-17.

7. Dong Z, Ma Y, Zhou H, et al. Integrated genomics analysis highlights important SNPs and genes implicated in moderate-to-severe asthma based on GWAS and eQTL datasets. *BMC pulmonary medicine.* 2020;20(1):1-16.

8. Xiang B, Deng C, Qiu F, et al. Single Cell Sequencing Analysis Identifies Genetics-Modulated ORMDL3+ Cholangiocytes Having Higher Metabolic Effects On Primary Biliary Cholangitis. *Journal of Nanobiotechnology.* 2021;19(1):406.

9. Lv Y, Xu X, Wang Z, Huang Y, Ma Y, Wu M. Integrated multi-omics data analysis identifies a novel genetics-risk gene of IRF4 associated with prognosis of oral cavity cancer. *Current Bioinformatics.* 2022;17(8):744-758.

10. Huang Y, Luo J, Zhang Y, et al. Identification of MKNK1 and TOP3A as ovarian endometriosis risk-associated genes using integrative genomic analyses and functional experiments. *Computational and Structural Biotechnology Journal.* 2023.

11. Szklarczyk D, Gable AL, Lyon D, et al. STRING v11: protein-protein association networks with increased coverage, supporting functional discovery in genome-wide experimental datasets. *Nucleic Acids Res.* 2019;47(D1):D607-d613.

12. Barbeira AN, Pividori M, Zheng J, Wheeler HE, Nicolae DL, Im HK. Integrating predicted transcriptome from multiple tissues improves association detection. *PLoS Genet.* 2019;15(1):e1007889.

13. Butler A, Hoffman P, Smibert P, Papalexi E, Satija R. Integrating single-cell transcriptomic data across different conditions, technologies, and species. *Nat Biotechnol.* 2018;36(5):411-420.

14. Ma Y, Qiu F, Deng C, et al. Integrating single-cell sequencing data with GWAS summary statistics reveals CD16+ monocytes and memory CD8+ T cells involved in severe COVID-19. *Genome medicine.* 2022;14(1):1-21.

15. Muhl L, Genové G, Leptidis S, et al. Single-cell analysis uncovers fibroblast heterogeneity and criteria for fibroblast and mural cell identification and discrimination. *Nat Commun.* 2020;11(1):3953.

16. Su C, Gao L, May CL, et al. 3D chromatin maps of the human pancreas reveal lineage-specific regulatory architecture of T2D risk. *Cell metabolism.* 2022;34(9):1394-1409. e1394.

17. Gulati GS, Sikandar SS, Wesche DJ, et al. Single-cell transcriptional diversity is a hallmark of developmental potential. *Science.* 2020;367(6476):405-411.

18. Qiu X, Mao Q, Tang Y, et al. Reversed graph embedding resolves complex single-cell trajectories. *Nature methods.* 2017;14(10):979-982.

19. Yu G, Wang L-G, Han Y, He Q-Y. clusterProfiler: an R package for comparing biological themes among gene clusters. *Omics: a journal of integrative biology.* 2012;16(5):284-287.

20. Zhang MJ, Hou K, Dey KK, et al. Polygenic enrichment distinguishes disease associations of individual cells in single-cell RNA-seq data. *Nat Genet.* 2022.

21. de Leeuw CA, Mooij JM, Heskes T, Posthuma D. MAGMA: generalized gene-set analysis of GWAS data. *PLoS Comput Biol.* 2015;11(4):e1004219.

22. Warde-Farley D, Donaldson SL, Comes O, et al. The GeneMANIA prediction server: biological network integration for gene prioritization and predicting gene function. *Nucleic acids research.* 2010;38(suppl_2):W214-W220.

23. Shannon P, Markiel A, Ozier O, et al. Cytoscape: a software environment for integrated models of biomolecular interaction networks. *Genome Res.* 2003;13(11):2498-2504.
