# Supplementary material for: Integration of human organoids single‐cell transcriptomic profiles and human genetics repurposes critical cell type‐specific drug targets for severe COVID‐19
Source: Cell Prolif. 2023 Oct 8;57(3):e13558. doi: 10.1111/cpr.13558 (PMC10905359; doi:10.1111/cpr.13558)
Supplement: Supplementary file 3 — Data S3. Supporting Tables. [file CPR-57-e13558-s002.pdf]

Supplementary Tables

Supplementary Table S1. Summary of scRNA-seq datasets on human fetal organs

Fetal Organs	Samples	Cell Count	Sequencing Platform	Accession No.	Data Source
Brain	8	46,985	10X Genomics	GSE119212	GEO
Kidney	1	4,176	10X Genomics	GSE102596	GEO
Liver	1	1,745	10X Genomics	E-MTAB-8210	ArrayExpress
Lung	11	35,848	10X Genomics	E-MTAB-8221	ArrayExpress
Pancreas	4	9,372	10X Genomics	GSE201230	GEO
Intestine	2	7,641	10X Genomics	E-MTAB-9363	ArrayExpress
Intestine	10	54,547	10X Genomics	E-MTAB-9489	ArrayExpress
Skin	7	63,020	10X Genomics	E-MTAB-7407	ArrayExpress
Eye	4	7,902	10X Genomics	GSE142526	GEO

Supplementary Table S2. Summary of GWAS summary statistics on three COVID-19 outcomes and 90 complex diseases and cancers used in the current study

Phenotypes	Year	Data ID	Population	Sample size	Number of SNPs	Resources
Very severe COVID-19	2022	A2_ALL_leave_23andme (Round 7)	Primary European	1,163,698	11,732,503	https://www.covid19hg.org/results/r7/
Hospitalized COVID-19	2022	B2_ALL_leave_23andme (Round 7)	Primary European	2,401,372	12,030,868	https://www.covid19hg.org/results/r7/
Susceptible COVID-19	2022	C2_ALL_leave_23andme (Round 7)	Primary European	2,942,817	14,335,927	https://www.covid19hg.org/results/r7/
Anorexia nervosa	2017	ieu-a-1186	European	14,477	10,641,224	https://gwas.mrcieu.ac.uk/datasets/ieu-a-1186/
Autism spectrum disorder	2017	ieu-a-1185	European	46,351	9,112,386	https://gwas.mrcieu.ac.uk/datasets/ieu-a-1185/
Bipolar disorder	2019	ieu-b-41	European	51,710	13,413,244	https://gwas.mrcieu.ac.uk/datasets/ieu-b-41/
Attention deficit hyperactivity disorder	2017	ieu-a-1183	European	55,374	8,047,420	https://gwas.mrcieu.ac.uk/datasets/ieu-a-1183/
Major Depression Disease	2018	ukb-b-12064	European	462,933	9,851,867	https://gwas.mrcieu.ac.uk/datasets/ukb-b-12064/
Schizophrenia	2020	PGC	European	161,405	7,585,078	https://www.medrxiv.org/content/10.1101/2020.09.12.20192922v1.full.pdf
Insomnia	2019	NA	European	386,533	1,145,847	https://ctg.cncr.nl/software/summary_statistics
Multiple sclerosis	2019	ieu-b-18	European	115,803	6,304,359	https://gwas.mrcieu.ac.uk/datasets/ieu-b-18/
Migraine	2018	ukb-b-16868	European	462,933	9,851,867	https://gwas.mrcieu.ac.uk/datasets/ukb-b-16868/
Ischemic stroke	2018	ebi-a-GCST006908	European	440,328	8,296,492	https://gwas.mrcieu.ac.uk/datasets/ebi-a-GCST006908/
Epilepsy	2018	ieu-b-8	European	44,889	4,880,492	https://gwas.mrcieu.ac.uk/datasets/ieu-b-8/
Focal epilepsy	2018	ieu-b-10	European	39,348	4,862,782	https://gwas.mrcieu.ac.uk/datasets/ieu-b-10/
Generalized epilepsy	2018	ieu-b-9	European	33,446	4,867,068	https://gwas.mrcieu.ac.uk/datasets/ieu-b-9/
Juvenile myoclonic epilepsy	2018	ieu-b-17	European	30,858	4,983,225	https://gwas.mrcieu.ac.uk/datasets/ieu-b-17/
Alzheimer's disease	2019	ieu-b-2	European	63,926	10,528,610	https://gwas.mrcieu.ac.uk/datasets/ieu-b-2/
Parkinson's disease	2019	ieu-b-7	European	482,730	17,891,936	https://gwas.mrcieu.ac.uk/datasets/ieu-b-7/
Snoring	2019	NA	European	359,916	1,146,184	https://ctg.cncr.nl/software/summary_statistics
Daytime Napping	2019	NA	European	386,577	1,160,017	https://ctg.cncr.nl/software/summary_statistics

Happiness	2018	ukb-b-4062	European	152,348	9,851,867	https://gwas.mrcieu.ac.uk/datasets/ukb-b-4062/
Cigarettes smoked per day	2019	ieu-b-142	European	249,752	12,003,613	https://gwas.mrcieu.ac.uk/datasets/ieu-b-142/
Extraversion	2012	ieu-a-115	European	17,375	2,305,823	https://gwas.mrcieu.ac.uk/files/ieu-a-115/ieu-a-115.vcf.gz
Subjective well-being	2016	ebi-a-GCST003766	European	298,420	2,058,391	https://gwas.mrcieu.ac.uk/datasets/ebi-a-GCST003766/
Conscientiousness	2010	ebi-a-GCST006326	European	17,375	2,300,083	https://gwas.mrcieu.ac.uk/files/ebi-a-GCST006326/ebi-a-GCST006326.vcf.gz
Openness to experience	2012	ieu-a-117	European	17,375	2,305,641	https://gwas.mrcieu.ac.uk/datasets/ieu-a-117/
Neuroticism	2017	ebi-a-GCST005232	European	329,821	18,436,568	https://gwas.mrcieu.ac.uk/datasets/ebi-a-GCST005232/
Cognitive Performance	2018	ebi-a-GCST006572	European	257,841	10,066,414	https://gwas.mrcieu.ac.uk/datasets/ebi-a-GCST006572/
Smoking cessation	2019	bbj-a-81	European	76,047	5,961,480	https://gwas.mrcieu.ac.uk/datasets/bbj-a-81/
Age of smoking initiation	2019	ieu-b-24	European	341,427	11,894,779	https://gwas.mrcieu.ac.uk/datasets/ieu-b-24/
Hypertension	2018	ukb-b-14057	European	462,933	9,851,867	https://gwas.mrcieu.ac.uk/datasets/ukb-b-14057/
Systolic blood pressure	2019	bbj-a-46	European	757,601	7,088,083	https://gwas.mrcieu.ac.uk/datasets/bbj-a-46/
Pulse pressure	2019	bbj-a-46	East Asian	136,249	6,108,953	https://gwas.mrcieu.ac.uk/datasets/bbj-a-46/
High blood pressure	2018	ukb-b-14177	European	124,227	9,851,867	https://gwas.mrcieu.ac.uk/datasets/ukb-b-14177/
Diastolic blood pressure	2018	ieu-b-39	European	757,601	7,160,619	https://gwas.mrcieu.ac.uk/datasets/ieu-b-39/
Diastolic blood pressure	2019	bbj-a-17	East Asian	136,615	6,108,953	https://gwas.mrcieu.ac.uk/datasets/bbj-a-17/
Diastolic blood pressure	2019	ebi-a-GCST008029	Hispanic	21,549	31,859,670	https://gwas.mrcieu.ac.uk/datasets/ebi-a-GCST008029/
Coronary artery disease	2017	ebi-a-GCST005195	European	547,261	7,934,254	https://gwas.mrcieu.ac.uk/datasets/ebi-a-GCST005195/
Type II diabetes	2018	ebi-a-GCST006867	European	655,666	5,030,727	https://gwas.mrcieu.ac.uk/datasets/ebi-a-GCST006867/
Ulcerative Colitis	2017	ebi-a-GCST004133	Mixed	45,975	9,474,559	https://gwas.mrcieu.ac.uk/datasets/ebi-a-GCST004133/
Psoriasis	2012	ebi-a-GCST005527	European	33,394	138,661	https://gwas.mrcieu.ac.uk/datasets/ebi-a-GCST005527/
Coeliac disease	2010	ebi-a-GCST000612	European	15,283	528,969	https://gwas.mrcieu.ac.uk/datasets/ebi-a-GCST000612/
Primary biliary cholangitis	2015	ebi-a-GCST003129	European	13,239	1,124,241	https://gwas.mrcieu.ac.uk/datasets/ebi-a-GCST003129/
Rheumatoid arthritis	2014	ieu-a-833	Mixed	80,799	9,739,304	https://gwas.mrcieu.ac.uk/datasets/ieu-a-833/
Inflammatory bowel disease	2017	ebi-a-GCST004131	Mixed	59,957	9,619,016	https://gwas.mrcieu.ac.uk/datasets/ebi-a-GCST004131/
Systemic lupus erythematosus	2015	ebi-a-GCST003156	European	14,267	7,071,163	https://gwas.mrcieu.ac.uk/datasets/ebi-a-GCST003156/
Waist Circumference	2015	ieu-a-60	European	224,459	2,566,630	https://gwas.mrcieu.ac.uk/datasets/ieu-a-60/
Total cholesterol	2013	ieu-a-301	European	187,365	2,446,982	https://gwas.mrcieu.ac.uk/datasets/ieu-a-301/
Chronic kidney disease	2016	ebi-a-GCST003374	European	117,165	2,179,497	https://gwas.mrcieu.ac.uk/datasets/ebi-a-GCST003374/
Body fat percentage	2018	ukb-b-8909	European	454,633	9,851,867	https://gwas.mrcieu.ac.uk/datasets/ukb-b-8909/

Triglyceride level	2017	ebi-a-GCST005073	European	9,745	23,163,055	https://gwas.mrcieu.ac.uk/datasets/ebi-a-GCST005073/
High-density lipoprotein (HDL) cholesterol	2013	ieu-a-299	European	187,167	2,447,442	https://gwas.mrcieu.ac.uk/datasets/ieu-a-299/
Low-density lipoprotein (LDL) cholesterol	2013	ieu-a-300	European	173,082	2,437,752	https://gwas.mrcieu.ac.uk/datasets/ieu-a-300/
Tuberculosis	2018	ukb-b-15622	European	462,933	9,851,867	https://gwas.mrcieu.ac.uk/datasets/ukb-b-15622/
Adult asthma	2018	ukb-b-18113	European	462,933	9,851,867	https://gwas.mrcieu.ac.uk/datasets/ukb-b-18113/
Small cell lung cancer	2022	finngen_R8_C3_SCLC_EXALLC	European	260,160	20,166,402	https://www.finngen.fi/en/access_results
Pulmonary embolism	2018	ukb-b-16048	European	462,933	9,851,867	https://gwas.mrcieu.ac.uk/datasets/ukb-b-16048/
Pulmonary artery	2018	ukb-b-18616	European	463,010	9,851,867	https://gwas.mrcieu.ac.uk/datasets/ukb-b-18616/
Non-small cell lung cancer, squamous	2022	finngen_R8_C3_NSCLC_SQUAM	European	260,793	20,166,432	https://www.finngen.fi/en/access_results
Non-small cell lung cancer, adenocarcinoma	2022	finngen_R8_C3_NSCLC_ADENO	European	260,820	20,166,443	https://www.finngen.fi/en/access_results
Non-small cell lung cancer	2022	finngen_R8_C3_LUNG_NONSMALL	European	263,448	20,166,592	https://www.finngen.fi/en/access_results
Lung volume	2019	ebi-a-GCST90016668	European	32,860	9,275,407	https://gwas.mrcieu.ac.uk/datasets/ebi-a-GCST90016668/
Lung function (FVC)	2019	ebi-a-GCST007429	European	321,047	19,676,344	https://gwas.mrcieu.ac.uk/datasets/ebi-a-GCST007429/
Influenza without pneumonia	2021	finn-b-INFLUENZA	European	291,090	16,380,370	https://gwas.mrcieu.ac.uk/datasets/finn-b-INFLUENZA/
Influenza with pneumonia	2021	finn-b-J10_INFLUPNEU	European	342,499	16,380,466	https://gwas.mrcieu.ac.uk/datasets/finn-b-J10_INFLUPNEU/
All influenza	2021	finn-b-J10_INFLUENZA	European	193,130	16,380,378	https://gwas.mrcieu.ac.uk/datasets/finn-b-J10_INFLUENZA/
Emphysema/chronic bronchitis	2018	ukb-b-7280	European	462,933	9,851,867	https://gwas.mrcieu.ac.uk/datasets/ukb-b-7280/
Chronic obstructive pulmonary disease	2018	ukb-b-16751	European	463,010	9,851,867	https://gwas.mrcieu.ac.uk/datasets/ukb-b-16751/
Bladder cancer	2022	C3_BLADDER_EXALLC	European	240750	16383210	https://www.finngen.fi/fi
Brain cancer	2022	C3_BRAIN_EXALLC	European	239370	16383209	https://www.finngen.fi/fi
Breast cancer	2022	C3_BREAST_EXALLC	European	150685	16383209	https://www.finngen.fi/
Cervix uteri cancer	2022	C3_CERVIX_UTERI_EXALLC	European	140122	16382416	https://www.finngen.fi/
Colon cancer	2022	C3_COLON_EXALLC	European	241667	16383214	https://www.finngen.fi/
Endocrine cancer	2022	C3_ENDOCRINE_EXALLC	European	240219	16383210	https://www.finngen.fi/
Eye adnexa cancer	2022	C3_EYE_ADNEXA_EXALLC	European	238850	16383208	https://www.finngen.fi/
Kidney cancer	2022	C3_KIDNEY_NOTRENALPELVIS_EXALLC	European	240309	16383212	https://www.finngen.fi/
Liver intrahepatic bile ducts cancer	2022	C3_LIVER_INTRAHEPATIC_BILE_DUCTS_EXALLC	European	239196	16383209	https://www.finngen.fi/
Lung non-small cancer	2022	C3_LUNG_NONSMALL_EXALLC	European	241683	16383210	https://www.finngen.fi/
Male genital cancer	2022	C3_MALE_GENITAL_EXALLC	European	111934	16382074	https://www.finngen.fi/
Ovary cancer	2022	C3_OVARY_EXALLC	European	138691	16382404	https://www.finngen.fi/
Prostate cancer	2022	C3_PROSTATE_EXALLC	European	111585	16382073	https://www.finngen.fi/
Rectum cancer	2022	C3_RECTUM_EXALLC	European	240510	16383209	https://www.finngen.fi/
Small intestine cancer	2022	C3_SMALL_INTESTINE_EXALLC	European	239089	16383208	https://www.finngen.fi/

Thyroid gland cancer	2022	C3_THYROID_GLAND_EXALLC	European	240094	16383210	https://www.finngen.fi/
Urinary tract cancer	2022	C3_URINARY_TRACT_EXALLC	European	242517	16383215	https://www.finngen.fi/
Endometrial Carcinoma	2018	ebi-a-GCST006464	European	121885	9470555	https://www.finngen.fi/
Glioma	2013	ieu-a-1013	European	6811	309636	https://gwas.mrcieu.ac.uk/files/ieu-a-1013/ieu-a-1013.vcf.gz
Head and Neck cancer	2021	ieu-b-4912	European	373122	9655080	https://gwas.mrcieu.ac.uk/files/ieu-b-4912/ieu-b-4912.vcf.gz
Lung tumor	2021	ieu-b-4954	European	374687	11078115	https://gwas.mrcieu.ac.uk/files/ieu-b-4954/ieu-b-4954.vcf.gz
Colorectal Cancer	2021	ieu-b-4965	European	377673	11738639	https://gwas.mrcieu.ac.uk/files/ieu-b-4965/ieu-b-4965.vcf.gz
Melanoma	2021	ieu-b-4969	European	375767	11396019	https://gwas.mrcieu.ac.uk/files/ieu-b-4969/ieu-b-4969.vcf.gz
Kidney cancer	2018	ukb-b-1316	European	463010	9851867	https://gwas.mrcieu.ac.uk/files/ukb-b-1316/ukb-b-1316.vcf.gz

Supplementary Table S3. The percent of significant cell types and significant number of datasets for three COVID-19 outcomes using scPagwas

Human organoids	Cell types	Very Severe COVID-19		Hospitalized COVID-19		Susceptible Covid-19	
		Significant Percent	Significant datasets	Significant Percent	Significant datasets	Significant Percent	Significant datasets
Brain	Endothelial Cell	54.55%	12	22.73%	5	54.55%	12
Brain	Microglia	40.00%	2	40.00%	2	60.00%	3
Brain	Radial Glia	34.78%	8	4.35%	1	4.35%	1
Brain	Astrocytic	26.09%	6	4.35%	1	0.00%	0
Brain	IPC	26.09%	6	65.22%	15	26.09%	6
Brain	Excitatory Neuron	25.00%	6	8.33%	2	20.83%	5
Brain	Oligodendrocyte	20.00%	1	0.00%	1	0.00%	1
Brain	Inhibitory Neuron	8.33%	2	12.50%	3	66.67%	16
Brain	NPC	0.00%	0	13.64%	3	9.09%	2
Brain	OPC	0.00%	0	0.00%	0	100.00%	1
Lung	MSC	50.00%	1	50.00%	1	50.00%	1
Intestine	Membranous Cell	63.64%	7	45.45%	5	54.55%	6
Intestine	Enterocyte	50.00%	8	31.25%	5	43.75%	7
Intestine	Tuft	50.00%	6	50.00%	6	16.67%	2
Intestine	Stem Cell	17.65%	3	52.94%	9	41.18%	7
Intestine	Goblet	14.29%	1	14.29%	1	0.00%	0
Intestine	Enteroendocrine	7.69%	1	15.38%	2	23.08%	3
Kidney	NPC	70.00%	7	50.00%	5	50.00%	5
Kidney	DN	55.56%	5	0.00%	0	11.11%	1
Eye	Horizontal	75.00%	3	25.00%	1	25.00%	1
Eye	Rod	75.00%	3	25.00%	1	25.00%	1
Eye	RPC	50.00%	2	50.00%	2	25.00%	1
Eye	RPE	50.00%	1	0.00%	0	0.00%	0
Eye	Astrocytic	33.33%	1	0.00%	0	0.00%	0
Eye	Muller Glia	25.00%	1	25.00%	1	25.00%	1
Eye	Cone	25.00%	1	25.00%	1	25.00%	1
Eye	Bipolar Cell	0.00%	0	33.33%	1	33.33%	1

Eye	RGC	0.00%	0	25.00%	1	50.00%	2
Eye	Amacrine Cell	0.00%	0	0.00%	0	25.00%	1
Eye	PP	0.00%	0	0.00%	0	25.00%	1
Liver	Stellate	100.00%	6	0.00%	0	0.00%	0
Liver	Cholangiocyte	27.27%	3	9.09%	1	0.00%	0
Liver	Hepatocyte	20.00%	2	70.00%	7	100.00%	10
Pancreas	Endothelial Cell	100.00%	1	100.00%	1	100.00%	1
Pancreas	Alpha	100.00%	1	0.00%	0	100.00%	1
Pancreas	Ductal Cell	100.00%	1	0.00%	0	100.00%	1
Pancreas	Proliferating Cell	0.00%	0	100.00%	1	0.00%	0
Heart	Endothelial Cell	100.00%	1	100.00%	1	100.00%	1
Heart	Pluripotent Cell	100.00%	1	0.00%	0	100.00%	1

Supplementary Table S4. The 243 significant genes associated with very severe COVID-19 identified by S-MultiXcan analysis based on 49 GTEx tissues

Gene ID	Gene name	P value	FDR
ENSG00000163817.15	*SLC6A20*	7.16E-227	1.60E-222
ENSG00000173585.15	*CCR9*	6.50E-220	7.26E-216
ENSG00000172215.5	*CXCR6*	2.46E-118	1.83E-114
ENSG00000173578.7	*XCR1*	1.81E-112	1.01E-108
ENSG00000163818.16	*LZTFL1*	1.64E-101	7.33E-98
ENSG00000183625.14	*CCR3*	7.31E-62	2.72E-58
ENSG00000142002.16	*DPP9*	5.70E-51	1.82E-47
ENSG00000163823.3	*CCR1*	2.85E-50	7.96E-47
ENSG00000159110.19	*IFNAR2*	2.05E-47	5.09E-44
ENSG00000160791.13	*CCR5*	9.97E-47	2.23E-43
ENSG00000121807.5	*CCR2*	8.23E-45	1.67E-41
ENSG00000243646.9	*IL10RB*	4.16E-42	7.74E-39
ENSG00000163820.14	*FYCO1*	2.62E-41	4.50E-38
ENSG00000121797.9	*CCRL2*	7.73E-35	1.23E-31
ENSG00000173171.14	*MTX1*	1.52E-27	2.26E-24
ENSG00000185499.16	*MUC1*	5.20E-25	7.26E-22
ENSG00000116539.12	*ASH1L*	4.31E-22	5.66E-19
ENSG00000163462.17	*TRIM46*	1.68E-19	2.08E-16
ENSG00000137166.14	*FOXP4*	2.39E-17	2.79E-14
ENSG00000273102.1	*AP000569.9*	2.50E-17	2.79E-14
ENSG00000283646.1	*LINC02009*	1.41E-15	1.50E-12
ENSG00000105397.13	*TYK2*	1.92E-15	1.95E-12
ENSG00000169241.17	*SLC50A1*	1.78E-14	1.73E-11
ENSG00000241837.6	*ATP5O*	2.45E-14	2.28E-11
ENSG00000012223.12	*LTF*	6.51E-14	5.82E-11
ENSG00000204542.2	*C6orf15*	2.56E-13	2.20E-10
ENSG00000175164.13	*ABO*	4.92E-13	4.07E-10
ENSG00000143537.13	*ADAM15*	5.20E-13	4.15E-10

ENSG00000185361.8	*TNFAIP8L1*	1.00E-12	7.70E-10
ENSG00000243364.7	*EFNA4*	5.09E-12	3.79E-09
ENSG00000121691.4	*CAT*	9.25E-12	6.67E-09
ENSG00000116580.18	*GON4L*	2.16E-11	1.51E-08
ENSG00000243927.5	*MRPS6*	3.52E-11	2.38E-08
ENSG00000204536.13	*CCHCR1*	3.86E-11	2.54E-08
ENSG00000267198.1	*RP11-798G7.6*	9.93E-11	6.34E-08
ENSG00000065989.15	*PDE4A*	2.09E-10	1.30E-07
ENSG00000225190.10	*PLEKHM1*	2.29E-10	1.38E-07
ENSG00000131400.7	*NAPSA*	2.36E-10	1.39E-07
ENSG00000197536.11	*C5orf56*	5.25E-10	3.01E-07
ENSG00000204525.16	*HLA-C*	6.13E-10	3.42E-07
ENSG00000176920.11	*FUT2*	9.04E-10	4.93E-07
ENSG00000204538.3	*PSORS1C2*	1.07E-09	5.69E-07
ENSG00000135374.9	*ELF5*	1.30E-09	6.75E-07
ENSG00000159314.11	*ARHGAP27*	1.36E-09	6.90E-07
ENSG00000133661.15	*SFTPD*	1.94E-09	9.63E-07
ENSG00000176715.15	*ACSF3*	2.03E-09	9.86E-07
ENSG00000232300.1	*FAM215B*	2.12E-09	1.01E-06
ENSG00000137310.11	*TCF19*	2.17E-09	1.01E-06
ENSG00000267673.6	*FDX2*	2.37E-09	1.08E-06
ENSG00000068650.18	*ATP11A*	2.60E-09	1.16E-06
ENSG00000163463.11	*KRTCAP2*	3.02E-09	1.32E-06
ENSG00000105376.4	*ICAM5*	4.01E-09	1.72E-06
ENSG00000089127.12	*OAS1*	4.10E-09	1.73E-06
ENSG00000220201.7	*ZGLP1*	5.36E-09	2.22E-06
ENSG00000168743.12	*NPNT*	5.92E-09	2.40E-06
ENSG00000112787.12	*FBRSL1*	7.15E-09	2.85E-06
ENSG00000108379.9	*WNT3*	7.59E-09	2.97E-06
ENSG00000105538.9	*RASIP1*	1.11E-08	4.28E-06
ENSG00000121073.13	*SLC35B1*	1.21E-08	4.58E-06
ENSG00000163354.14	*DCST2*	1.41E-08	5.25E-06
ENSG00000176909.11	*MAMSTR*	1.60E-08	5.86E-06
ENSG00000142233.11	*NTN5*	1.63E-08	5.87E-06
ENSG00000206503.12	*HLA-A*	1.68E-08	5.96E-06
ENSG00000167680.15	*SEMA6B*	3.46E-08	1.21E-05
ENSG00000185303.16	*SFTPA2*	4.29E-08	1.47E-05
ENSG00000226676.1	*RP11-589B3.6*	4.88E-08	1.65E-05
ENSG00000211456.10	*SACM1L*	7.54E-08	2.51E-05
ENSG00000111331.12	*OAS3*	1.24E-07	4.07E-05
ENSG00000076662.9	*ICAM3*	1.41E-07	4.56E-05
ENSG00000182264.8	*IZUMO1*	1.55E-07	4.95E-05
ENSG00000244733.5	*RP11-506M13.3*	1.73E-07	5.44E-05
ENSG00000228570.7	*NUTM2E*	1.76E-07	5.46E-05
ENSG00000231485.1	*RP4-535B20.1*	1.88E-07	5.75E-05
ENSG00000163825.3	*RTP3*	2.08E-07	6.28E-05

ENSG00000104388.14	*RAB2A*	2.78E-07	8.28E-05
ENSG00000204540.10	*PSORS1C1*	3.12E-07	9.17E-05
ENSG00000090339.8	*ICAM1*	4.35E-07	1.26E-04
ENSG00000161847.13	*RAVER1*	5.26E-07	1.51E-04
ENSG00000272076.1	*RP11-11C20.3*	5.46E-07	1.54E-04
ENSG00000251396.6	*LINC01301*	5.82E-07	1.63E-04
ENSG00000174951.10	*FUT1*	6.25E-07	1.72E-04
ENSG00000166508.17	*MCM7*	7.28E-07	1.98E-04
ENSG00000179335.18	*CLK3*	9.45E-07	2.54E-04
ENSG00000198563.13	*DDX39B*	1.19E-06	3.16E-04
ENSG00000204619.7	*PPP1R11*	1.26E-06	3.31E-04
ENSG00000204539.3	*CDSN*	1.33E-06	3.45E-04
ENSG00000164398.12	*ACSL6*	1.44E-06	3.70E-04
ENSG00000096696.13	*DSP*	1.51E-06	3.83E-04
ENSG00000237541.3	*HLA-DQA2*	1.54E-06	3.87E-04
ENSG00000142166.12	*IFNAR1*	1.60E-06	3.97E-04
ENSG00000074842.7	*MYDGF*	1.86E-06	4.54E-04
ENSG00000272447.1	*RP11-182L21.6*	1.87E-06	4.54E-04
ENSG00000148730.6	*EIF4EBP2*	1.98E-06	4.76E-04
ENSG00000204287.13	*HLA-DRA*	2.22E-06	5.28E-04
ENSG00000238083.7	*LRRC37A2*	2.28E-06	5.33E-04
ENSG00000259803.6	*SLC22A31*	2.29E-06	5.33E-04
ENSG00000106261.16	*ZKSCAN1*	2.40E-06	5.53E-04
ENSG00000272666.1	*CTA-384D8.35*	3.17E-06	7.23E-04
ENSG00000170100.13	*ZNF778*	3.34E-06	7.54E-04
ENSG00000127946.16	*HIP1*	3.52E-06	7.86E-04
ENSG00000105401.8	*CDC37*	3.99E-06	8.82E-04
ENSG00000129910.7	*CDH15*	4.69E-06	1.03E-03
ENSG00000108344.14	*PSMD3*	4.86E-06	1.05E-03
ENSG00000230746.1	*AC006007.1*	4.92E-06	1.06E-03
ENSG00000131398.13	*KCNC3*	6.25E-06	1.32E-03
ENSG00000204642.13	*HLA-F*	6.25E-06	1.32E-03
ENSG00000204516.9	*MICB*	7.39E-06	1.54E-03
ENSG00000186827.10	*TNFRSF4*	7.61E-06	1.57E-03
ENSG00000196683.10	*TOMM7*	8.33E-06	1.71E-03
ENSG00000130733.10	*YIPF2*	8.69E-06	1.76E-03
ENSG00000203684.5	*IBA57-AS1*	1.08E-05	2.15E-03
ENSG00000144802.11	*NFKBIZ*	1.08E-05	2.15E-03
ENSG00000072682.18	*P4HA2*	1.13E-05	2.21E-03
ENSG00000115520.8	*COQ10B*	1.13E-05	2.21E-03
ENSG00000160505.15	*NLRP4*	1.36E-05	2.64E-03
ENSG00000213420.7	*GPC2*	1.39E-05	2.68E-03
ENSG00000188157.14	*AGRN*	1.42E-05	2.69E-03
ENSG00000168090.9	*COPS6*	1.42E-05	2.69E-03
ENSG00000176444.18	*CLK2*	1.82E-05	3.42E-03
ENSG00000160862.12	*AZGP1*	2.15E-05	4.00E-03

ENSG00000170608.2	*FOXA3*	2.24E-05	4.14E-03
ENSG00000143630.9	*HCN3*	2.30E-05	4.21E-03
ENSG00000162434.11	*JAK1*	2.40E-05	4.36E-03
ENSG00000038210.12	*PI4K2B*	2.45E-05	4.41E-03
ENSG00000116521.10	*SCAMP3*	2.50E-05	4.42E-03
ENSG00000180422.3	*LINC00304*	2.51E-05	4.42E-03
ENSG00000169242.11	*EFNA1*	2.52E-05	4.42E-03
ENSG00000160685.13	*ZBTB7B*	2.54E-05	4.42E-03
ENSG00000151498.11	*ACAD8*	2.55E-05	4.42E-03
ENSG00000242574.8	*HLA-DMB*	2.60E-05	4.47E-03
ENSG00000132972.18	*RNF17*	2.82E-05	4.81E-03
ENSG00000204574.12	*ABCF1*	2.88E-05	4.87E-03
ENSG00000142444.6	*TIMM29*	3.10E-05	5.21E-03
ENSG00000205015.1	*LINC02138*	3.33E-05	5.55E-03
ENSG00000185829.17	*ARL17A*	3.43E-05	5.68E-03
ENSG00000204580.13	*DDR1*	3.49E-05	5.73E-03
ENSG00000143622.10	*RIT1*	3.69E-05	6.02E-03
ENSG00000188199.10	*NUTM2B*	3.93E-05	6.36E-03
ENSG00000139725.7	*RHOF*	4.38E-05	7.04E-03
ENSG00000132718.8	*SYT11*	4.61E-05	7.36E-03
ENSG00000166997.7	*CNPY4*	4.78E-05	7.57E-03
ENSG00000151914.19	*DST*	4.97E-05	7.82E-03
ENSG00000166016.5	*ABTB2*	5.05E-05	7.89E-03
ENSG00000162613.16	*FUBP1*	5.10E-05	7.91E-03
ENSG00000163344.5	*PMVK*	5.35E-05	8.24E-03
ENSG00000126214.21	*KLC1*	5.61E-05	8.58E-03
ENSG00000185294.6	*SPPL2C*	5.70E-05	8.62E-03
ENSG00000163346.16	*PBXIP1*	5.71E-05	8.62E-03
ENSG00000108342.12	*CSF3*	5.78E-05	8.67E-03
ENSG00000247626.4	*MARS2*	6.25E-05	9.28E-03
ENSG00000078967.12	*UBE2D4*	6.27E-05	9.28E-03
ENSG00000160752.14	*FDPS*	6.32E-05	9.29E-03
ENSG00000256053.7	*APOPT1*	6.54E-05	9.55E-03
ENSG00000226979.8	*LTA*	6.60E-05	9.57E-03
ENSG00000239521.7	*GATS*	6.76E-05	9.74E-03
ENSG00000117682.16	*DHDDS*	6.88E-05	9.77E-03
ENSG00000171840.11	*NINJ2*	6.89E-05	9.77E-03
ENSG00000008838.19	*MED24*	6.91E-05	9.77E-03
ENSG00000204356.13	*NELFE*	7.86E-05	1.10E-02
ENSG00000263893.2	*CTD-3010D24.3*	7.94E-05	1.11E-02
ENSG00000162616.8	*DNAJB4*	8.40E-05	1.16E-02
ENSG00000087266.15	*SH3BP2*	8.47E-05	1.16E-02
ENSG00000118200.14	*CAMSAP2*	8.53E-05	1.16E-02
ENSG00000168228.14	*ZCCHC4*	8.54E-05	1.16E-02
ENSG00000165424.6	*ZCCHC24*	8.93E-05	1.21E-02
ENSG00000021461.16	*CYP3A43*	9.26E-05	1.25E-02

ENSG00000213722.8	*DDAH2*	9.84E-05	1.32E-02
ENSG00000225180.7	*AATK-AS1*	1.00E-04	1.32E-02
ENSG00000204610.12	*TRIM15*	1.00E-04	1.32E-02
ENSG00000053900.10	*ANAPC4*	1.01E-04	1.32E-02
ENSG00000143590.13	*EFNA3*	1.01E-04	1.32E-02
ENSG00000143443.9	*C1orf56*	1.07E-04	1.39E-02
ENSG00000197093.10	*GAL3ST4*	1.10E-04	1.42E-02
ENSG00000163746.11	*PLSCR2*	1.19E-04	1.52E-02
ENSG00000241370.5	*RPP21*	1.28E-04	1.64E-02
ENSG00000119596.17	*YLPM1*	1.31E-04	1.66E-02
ENSG00000204290.10	*BTNL2*	1.33E-04	1.67E-02
ENSG00000163374.19	*YY1AP1*	1.33E-04	1.67E-02
ENSG00000137478.14	*FCHSD2*	1.34E-04	1.67E-02
ENSG00000228862.3	*RP11-91I20.3*	1.42E-04	1.77E-02
ENSG00000122483.17	*CCDC18*	1.51E-04	1.86E-02
ENSG00000213676.10	*ATF6B*	1.53E-04	1.87E-02
ENSG00000162437.14	*RAVER2*	1.56E-04	1.91E-02
ENSG00000217128.11	*FNIP1*	1.60E-04	1.94E-02
ENSG00000188186.10	*LAMTOR4*	1.65E-04	1.98E-02
ENSG00000197208.5	*SLC22A4*	1.65E-04	1.98E-02
ENSG00000267011.5	*CTB-50L17.16*	1.66E-04	1.98E-02
ENSG00000080819.6	*CPOX*	1.79E-04	2.13E-02
ENSG00000198685.3	*LINC01565*	1.82E-04	2.15E-02
ENSG00000163467.11	*TSACC*	1.95E-04	2.29E-02
ENSG00000145495.15	*6-Mar*	1.97E-04	2.30E-02
ENSG00000179361.17	*ARID3B*	2.07E-04	2.40E-02
ENSG00000129354.11	*AP1M2*	2.11E-04	2.43E-02
ENSG00000160767.20	*FAM189B*	2.11E-04	2.43E-02
ENSG00000103355.13	*PRSS33*	2.21E-04	2.53E-02
ENSG00000256875.2	*RP11-503G7.2*	2.34E-04	2.67E-02
ENSG00000185507.19	*IRF7*	2.40E-04	2.73E-02
ENSG00000243649.8	*CFB*	2.48E-04	2.80E-02
ENSG00000241186.9	*TDGF1*	2.50E-04	2.81E-02
ENSG00000120907.17	*ADRA1A*	2.51E-04	2.81E-02
ENSG00000204174.7	*NPY4R*	2.54E-04	2.82E-02
ENSG00000170791.17	*CHCHD7*	2.70E-04	2.98E-02
ENSG00000115541.10	*HSPE1*	2.81E-04	3.09E-02
ENSG00000198743.6	*SLC5A3*	2.97E-04	3.23E-02
ENSG00000165841.9	*CYP2C19*	2.97E-04	3.23E-02
ENSG00000117505.12	*DR1*	2.98E-04	3.23E-02
ENSG00000172340.14	*SUCLG2*	3.00E-04	3.24E-02
ENSG00000152382.5	*TADA1*	3.06E-04	3.29E-02
ENSG00000110711.9	*AIP*	3.17E-04	3.39E-02
ENSG00000166526.16	*ZNF3*	3.24E-04	3.43E-02
ENSG00000146278.10	*PNRC1*	3.24E-04	3.43E-02
ENSG00000087253.12	*LPCAT2*	3.31E-04	3.48E-02

ENSG00000168010.10	*ATG16L2*	3.34E-04	3.50E-02
ENSG00000150540.13	*HNMT*	3.37E-04	3.52E-02
ENSG00000163902.11	*RPN1*	3.52E-04	3.65E-02
ENSG00000132793.11	*LPIN3*	3.55E-04	3.65E-02
ENSG00000120235.4	*IFNA6*	3.55E-04	3.65E-02
ENSG00000236699.8	*ARHGEF38*	3.63E-04	3.72E-02
ENSG00000198835.3	*GJC2*	3.70E-04	3.77E-02
ENSG00000132676.15	*DAP3*	3.80E-04	3.86E-02
ENSG00000105516.10	*DBP*	3.96E-04	4.00E-02
ENSG00000183682.7	*BMP8A*	4.00E-04	4.03E-02
ENSG00000105559.11	*PLEKHA4*	4.08E-04	4.09E-02
ENSG00000182168.14	*UNC5C*	4.11E-04	4.10E-02
ENSG00000186868.15	*MAPT*	4.21E-04	4.17E-02
ENSG00000227507.2	*LTB*	4.23E-04	4.18E-02
ENSG00000204438.10	*GPANK1*	4.37E-04	4.29E-02
ENSG00000204531.17	*POU5F1*	4.38E-04	4.29E-02
ENSG00000120071.13	*KANSL1*	4.40E-04	4.29E-02
ENSG00000132680.10	*KIAA0907*	4.52E-04	4.37E-02
ENSG00000167914.11	*GSDMA*	4.52E-04	4.37E-02
ENSG00000197102.10	*DYNC1H1*	4.58E-04	4.41E-02
ENSG00000160785.13	*SLC25A44*	4.61E-04	4.42E-02
ENSG00000166589.12	*CDH16*	4.78E-04	4.56E-02
ENSG00000204396.10	*VWA7*	5.00E-04	4.75E-02
ENSG00000204252.13	*HLA-DOA*	5.12E-04	4.85E-02
ENSG00000162944.10	*RFTN2*	5.16E-04	4.86E-02
ENSG00000130164.13	*LDLR*	5.19E-04	4.88E-02
ENSG00000135144.7	*DTX1*	5.25E-04	4.90E-02
ENSG00000177103.13	*DSCAML1*	5.27E-04	4.90E-02
ENSG00000188501.11	*LCTL*	5.30E-04	4.91E-02
ENSG00000104804.7	*TULP2*	5.34E-04	4.93E-02
ENSG00000169567.11	*HINT1*	5.38E-04	4.95E-02

Supplementary Table S5. The 277 significant genes associated with hospitalized COVID-19 identified by S-MultiXcan analysis based on 49 GTEx tissues

Gene ID	Gene name	P value	FDR
ENSG00000173585.15	*CCR9*	3.04E-229	6.79E-225
ENSG00000163817.15	*SLC6A20*	2.30E-217	2.57E-213
ENSG00000172215.5	*CXCR6*	7.53E-114	5.61E-110
ENSG00000163818.16	*LZTFL1*	5.65E-100	3.16E-96
ENSG00000173578.7	*XCR1*	1.53E-86	6.84E-83
ENSG00000183625.14	*CCR3*	6.93E-57	2.58E-53
ENSG00000163820.14	*FYCO1*	2.54E-53	8.11E-50
ENSG00000159110.19	*IFNAR2*	1.67E-50	4.66E-47
ENSG00000163823.3	*CCR1*	5.25E-46	1.30E-42
ENSG00000142002.16	*DPP9*	1.11E-41	2.48E-38

ENSG00000243646.9	*IL10RB*	4.82E-41	9.79E-38
ENSG00000160791.13	*CCR5*	7.76E-39	1.44E-35
ENSG00000121807.5	*CCR2*	5.43E-38	9.33E-35
ENSG00000121797.9	*CCRL2*	5.12E-35	8.17E-32
ENSG00000137166.14	*FOXP4*	5.15E-26	7.67E-23
ENSG00000185499.16	*MUC1*	2.21E-24	3.09E-21
ENSG00000175164.13	*ABO*	9.66E-23	1.27E-19
ENSG00000173171.14	*MTX1*	4.60E-22	5.71E-19
ENSG00000273102.1	*AP000569.9*	5.59E-21	6.57E-18
ENSG00000241837.6	*ATP5O*	4.95E-18	5.53E-15
ENSG00000116539.12	*ASH1L*	3.14E-17	3.34E-14
ENSG00000131400.7	*NAPSA*	4.33E-17	4.40E-14
ENSG00000243927.5	*MRPS6*	2.59E-16	2.52E-13
ENSG00000283646.1	*LINC02009*	4.35E-16	4.05E-13
ENSG00000135374.9	*ELF5*	1.81E-14	1.62E-11
ENSG00000121691.4	*CAT*	3.20E-14	2.75E-11
ENSG00000012223.12	*LTF*	5.44E-14	4.50E-11
ENSG00000144802.11	*NFKBIZ*	6.81E-13	5.43E-10
ENSG00000068650.18	*ATP11A*	2.53E-12	1.95E-09
ENSG00000163462.17	*TRIM46*	8.52E-12	6.34E-09
ENSG00000143537.13	*ADAM15*	9.88E-12	7.12E-09
ENSG00000267198.1	*RP11-798G7.6*	1.60E-11	1.12E-08
ENSG00000169241.17	*SLC50A1*	2.12E-11	1.44E-08
ENSG00000112787.12	*FBRSL1*	2.24E-11	1.47E-08
ENSG00000204542.2	*C6orf15*	6.91E-11	4.41E-08
ENSG00000163463.11	*KRTCAP2*	7.93E-11	4.92E-08
ENSG00000111331.12	*OAS3*	9.33E-11	5.63E-08
ENSG00000144815.15	*NXPE3*	1.06E-10	6.23E-08
ENSG00000267673.6	*FDX2*	1.21E-10	6.93E-08
ENSG00000204536.13	*CCHCR1*	1.38E-10	7.71E-08
ENSG00000133661.15	*SFTPD*	3.46E-10	1.89E-07
ENSG00000220201.7	*ZGLP1*	3.84E-10	2.04E-07
ENSG00000137310.11	*TCF19*	4.83E-10	2.51E-07
ENSG00000159314.11	*ARHGAP27*	6.74E-10	3.42E-07
ENSG00000121073.13	*SLC35B1*	7.18E-10	3.56E-07
ENSG00000204525.16	*HLA-C*	9.51E-10	4.58E-07
ENSG00000197536.11	*C5orf56*	9.66E-10	4.58E-07
ENSG00000198563.13	*DDX39B*	9.84E-10	4.58E-07
ENSG00000105397.13	*TYK2*	1.41E-09	6.34E-07
ENSG00000176920.11	*FUT2*	1.42E-09	6.34E-07
ENSG00000225190.10	*PLEKHM1*	1.67E-09	7.31E-07
ENSG00000232300.1	*FAM215B*	1.71E-09	7.35E-07
ENSG00000065989.15	*PDE4A*	2.66E-09	1.12E-06
ENSG00000161847.13	*RAVER1*	2.81E-09	1.16E-06
ENSG00000182504.10	*CEP97*	2.92E-09	1.19E-06
ENSG00000076662.9	*ICAM3*	4.54E-09	1.81E-06

ENSG00000168228.14	*ZCCHC4*	4.68E-09	1.82E-06
ENSG00000131398.13	*KCNC3*	4.73E-09	1.82E-06
ENSG00000066422.4	*ZBTB11*	5.48E-09	2.07E-06
ENSG00000185361.8	*TNFAIP8L1*	6.77E-09	2.52E-06
ENSG00000108379.9	*WNT3*	9.15E-09	3.35E-06
ENSG00000038210.12	*PI4K2B*	2.11E-08	7.60E-06
ENSG00000204538.3	*PSORS1C2*	3.28E-08	1.16E-05
ENSG00000185303.16	*SFTPA2*	4.27E-08	1.49E-05
ENSG00000129910.7	*CDH15*	4.59E-08	1.58E-05
ENSG00000251396.6	*LINC01301*	4.93E-08	1.67E-05
ENSG00000104388.14	*RAB2A*	5.91E-08	1.97E-05
ENSG00000089127.12	*OAS1*	9.67E-08	3.18E-05
ENSG00000203684.5	*IBA57-AS1*	1.05E-07	3.40E-05
ENSG00000133884.9	*DPF2*	1.33E-07	4.24E-05
ENSG00000163344.5	*PMVK*	1.63E-07	5.13E-05
ENSG00000103653.16	*CSK*	1.86E-07	5.77E-05
ENSG00000204516.9	*MICB*	1.94E-07	5.94E-05
ENSG00000226676.1	*RP11-589B3.6*	2.69E-07	8.12E-05
ENSG00000185829.17	*ARL17A*	2.76E-07	8.22E-05
ENSG00000163825.3	*RTP3*	3.72E-07	1.09E-04
ENSG00000243364.7	*EFNA4*	4.83E-07	1.40E-04
ENSG00000168743.12	*NPNT*	5.17E-07	1.48E-04
ENSG00000211456.10	*SACM1L*	5.31E-07	1.50E-04
ENSG00000197208.5	*SLC22A4*	6.01E-07	1.68E-04
ENSG00000176715.15	*ACSF3*	7.10E-07	1.96E-04
ENSG00000115520.8	*COQ10B*	7.76E-07	2.11E-04
ENSG00000226979.8	*LTA*	8.00E-07	2.15E-04
ENSG00000238083.7	*LRRC37A2*	8.62E-07	2.29E-04
ENSG00000142233.11	*NTN5*	1.07E-06	2.81E-04
ENSG00000166526.16	*ZNF3*	1.09E-06	2.83E-04
ENSG00000170100.13	*ZNF778*	1.21E-06	3.11E-04
ENSG00000198743.6	*SLC5A3*	1.32E-06	3.35E-04
ENSG00000096088.16	*PGC*	1.46E-06	3.66E-04
ENSG00000170608.2	*FOXA3*	1.72E-06	4.22E-04
ENSG00000105376.4	*ICAM5*	1.72E-06	4.22E-04
ENSG00000135144.7	*DTX1*	1.84E-06	4.47E-04
ENSG00000053900.10	*ANAPC4*	1.99E-06	4.78E-04
ENSG00000259803.6	*SLC22A31*	2.06E-06	4.90E-04
ENSG00000106261.16	*ZKSCAN1*	2.26E-06	5.26E-04
ENSG00000074842.7	*MYDGF*	2.26E-06	5.26E-04
ENSG00000112578.9	*BYSL*	3.12E-06	7.19E-04
ENSG00000166508.17	*MCM7*	3.18E-06	7.25E-04
ENSG00000164398.12	*ACSL6*	4.66E-06	1.05E-03
ENSG00000090339.8	*ICAM1*	4.70E-06	1.05E-03
ENSG00000272447.1	*RP11-182L21.6*	4.90E-06	1.08E-03
ENSG00000204287.13	*HLA-DRA*	5.00E-06	1.10E-03

ENSG00000162613.16	*FUBP1*	5.13E-06	1.10E-03
ENSG00000087266.15	*SH3BP2*	5.14E-06	1.10E-03
ENSG00000221843.4	*C2orf16*	5.63E-06	1.20E-03
ENSG00000116580.18	*GON4L*	5.81E-06	1.22E-03
ENSG00000081154.11	*PCNP*	6.17E-06	1.29E-03
ENSG00000138468.15	*SENP7*	6.32E-06	1.31E-03
ENSG00000239521.7	*GATS*	7.30E-06	1.49E-03
ENSG00000160752.14	*FDPS*	7.32E-06	1.49E-03
ENSG00000231485.1	*RP4-535B20.1*	7.79E-06	1.57E-03
ENSG00000140465.13	*CYP1A1*	8.19E-06	1.63E-03
ENSG00000223865.10	*HLA-DPB1*	1.03E-05	2.04E-03
ENSG00000204531.17	*POU5F1*	1.09E-05	2.14E-03
ENSG00000269387.1	*RP11-298J23.8*	1.23E-05	2.39E-03
ENSG00000162616.8	*DNAJB4*	1.36E-05	2.62E-03
ENSG00000137218.10	*FRS3*	1.38E-05	2.63E-03
ENSG00000204540.10	*PSORS1C1*	1.39E-05	2.63E-03
ENSG00000213676.10	*ATF6B*	1.45E-05	2.70E-03
ENSG00000108344.14	*PSMD3*	1.45E-05	2.70E-03
ENSG00000143774.16	*GUK1*	1.58E-05	2.92E-03
ENSG00000163354.14	*DCST2*	1.63E-05	2.98E-03
ENSG00000124593.15	*RP11-298J23.10*	1.80E-05	3.27E-03
ENSG00000213420.7	*GPC2*	2.03E-05	3.66E-03
ENSG00000277186.1	*RP13-554M15.8*	2.10E-05	3.75E-03
ENSG00000196683.10	*TOMM7*	2.23E-05	3.95E-03
ENSG00000204610.12	*TRIM15*	2.70E-05	4.75E-03
ENSG00000204290.10	*BTNL2*	2.97E-05	5.18E-03
ENSG00000180422.3	*LINC00304*	3.06E-05	5.30E-03
ENSG00000168090.9	*COPS6*	3.11E-05	5.34E-03
ENSG00000227507.2	*LTB*	3.44E-05	5.87E-03
ENSG00000147655.10	*RSPO2*	3.59E-05	6.08E-03
ENSG00000122852.14	*SFTPA1*	3.68E-05	6.18E-03
ENSG00000244733.5	*RP11-506M13.3*	3.90E-05	6.50E-03
ENSG00000160685.13	*ZBTB7B*	4.10E-05	6.78E-03
ENSG00000142687.17	*KIAA0319L*	4.19E-05	6.85E-03
ENSG00000227059.6	*ANHX*	4.20E-05	6.85E-03
ENSG00000278505.4	*C17orf78*	4.24E-05	6.86E-03
ENSG00000143793.12	*C1orf35*	4.32E-05	6.94E-03
ENSG00000105538.9	*RASIP1*	4.51E-05	7.20E-03
ENSG00000167183.2	*PRR15L*	4.65E-05	7.36E-03
ENSG00000142166.12	*IFNAR1*	4.71E-05	7.36E-03
ENSG00000204344.14	*STK19*	4.72E-05	7.36E-03
ENSG00000125967.16	*NECAB3*	4.77E-05	7.36E-03
ENSG00000120235.4	*IFNA6*	4.78E-05	7.36E-03
ENSG00000204642.13	*HLA-F*	5.27E-05	8.06E-03
ENSG00000278224.5	*PRICKLE4*	5.55E-05	8.43E-03
ENSG00000175130.6	*MARCKSL1*	6.52E-05	9.84E-03

ENSG00000228570.7	*NUTM2E*	7.04E-05	1.06E-02
ENSG00000266010.1	*GATA6-AS1*	7.34E-05	1.09E-02
ENSG00000168487.17	*BMP1*	7.38E-05	1.09E-02
ENSG00000234745.10	*HLA-B*	7.45E-05	1.09E-02
ENSG00000120071.13	*KANSL1*	7.69E-05	1.12E-02
ENSG00000152582.13	*SPEF2*	7.91E-05	1.15E-02
ENSG00000166016.5	*ABTB2*	8.15E-05	1.17E-02
ENSG00000204356.13	*NELFE*	8.15E-05	1.17E-02
ENSG00000166529.14	*ZSCAN21*	8.23E-05	1.17E-02
ENSG00000188199.10	*NUTM2B*	8.69E-05	1.23E-02
ENSG00000156587.15	*UBE2L6*	8.72E-05	1.23E-02
ENSG00000249931.4	*GOLGA8K*	8.92E-05	1.25E-02
ENSG00000116819.6	*TFAP2E*	9.23E-05	1.28E-02
ENSG00000148737.16	*TCF7L2*	9.52E-05	1.31E-02
ENSG00000125755.18	*SYMPK*	9.69E-05	1.33E-02
ENSG00000164463.12	*CREBRF*	1.04E-04	1.42E-02
ENSG00000125459.14	*MSTO1*	1.07E-04	1.45E-02
ENSG00000122359.17	*ANXA11*	1.07E-04	1.45E-02
ENSG00000008838.19	*MED24*	1.10E-04	1.47E-02
ENSG00000121067.17	*SPOP*	1.14E-04	1.51E-02
ENSG00000272076.1	*RP11-11C20.3*	1.24E-04	1.64E-02
ENSG00000228696.8	*ARL17B*	1.28E-04	1.67E-02
ENSG00000204387.12	*C6orf48*	1.28E-04	1.67E-02
ENSG00000111335.12	*OAS2*	1.33E-04	1.72E-02
ENSG00000186868.15	*MAPT*	1.35E-04	1.75E-02
ENSG00000034713.7	*GABARAPL2*	1.37E-04	1.76E-02
ENSG00000142973.13	*CYP4B1*	1.40E-04	1.79E-02
ENSG00000108342.12	*CSF3*	1.42E-04	1.80E-02
ENSG00000179151.11	*EDC3*	1.45E-04	1.82E-02
ENSG00000243649.8	*CFB*	1.48E-04	1.86E-02
ENSG00000105401.8	*CDC37*	1.52E-04	1.89E-02
ENSG00000141027.20	*NCOR1*	1.53E-04	1.89E-02
ENSG00000028528.14	*SNX1*	1.53E-04	1.89E-02
ENSG00000124721.17	*DNAH8*	1.56E-04	1.91E-02
ENSG00000137767.13	*SQRDL*	1.59E-04	1.94E-02
ENSG00000267505.1	*CTC-296K1.3*	1.64E-04	1.99E-02
ENSG00000159147.17	*DONSON*	1.66E-04	2.00E-02
ENSG00000186075.12	*ZPBP2*	1.66E-04	2.00E-02
ENSG00000163346.16	*PBXIP1*	1.67E-04	2.00E-02
ENSG00000204539.3	*CDSN*	1.68E-04	2.00E-02
ENSG00000020129.15	*NCDN*	1.74E-04	2.06E-02
ENSG00000181873.12	*IBA57*	1.77E-04	2.08E-02
ENSG00000179218.13	*CALR*	1.78E-04	2.08E-02
ENSG00000126091.19	*ST3GAL3*	1.80E-04	2.10E-02
ENSG00000164031.16	*DNAJB14*	1.81E-04	2.10E-02
ENSG00000125743.10	*SNRPD2*	1.87E-04	2.16E-02

ENSG00000176909.11	*MAMSTR*	1.89E-04	2.16E-02
ENSG00000163357.10	*DCST1*	1.96E-04	2.23E-02
ENSG00000172057.9	*ORMDL3*	1.98E-04	2.23E-02
ENSG00000166997.7	*CNPY4*	1.98E-04	2.23E-02
ENSG00000162434.11	*JAK1*	2.06E-04	2.31E-02
ENSG00000126214.21	*KLC1*	2.13E-04	2.38E-02
ENSG00000204520.12	*MICA*	2.14E-04	2.38E-02
ENSG00000230174.1	*LINC01149*	2.27E-04	2.51E-02
ENSG00000106290.14	*TAF6*	2.29E-04	2.51E-02
ENSG00000204438.10	*GPANK1*	2.29E-04	2.51E-02
ENSG00000227456.7	*LINC00310*	2.34E-04	2.54E-02
ENSG00000075539.13	*FRYL*	2.35E-04	2.54E-02
ENSG00000101412.12	*E2F1*	2.35E-04	2.54E-02
ENSG00000204314.10	*PRRT1*	2.39E-04	2.56E-02
ENSG00000237523.1	*LINC00857*	2.43E-04	2.60E-02
ENSG00000214941.7	*ZSWIM7*	2.47E-04	2.63E-02
ENSG00000197093.10	*GAL3ST4*	2.55E-04	2.69E-02
ENSG00000171103.10	*TRMT61B*	2.55E-04	2.69E-02
ENSG00000198835.3	*GJC2*	2.57E-04	2.69E-02
ENSG00000126067.11	*PSMB2*	2.59E-04	2.69E-02
ENSG00000122420.9	*PTGFR*	2.60E-04	2.69E-02
ENSG00000111237.18	*VPS29*	2.60E-04	2.69E-02
ENSG00000204256.12	*BRD2*	2.65E-04	2.73E-02
ENSG00000143590.13	*EFNA3*	2.67E-04	2.74E-02
ENSG00000247626.4	*MARS2*	2.72E-04	2.77E-02
ENSG00000272666.1	*CTA-384D8.35*	2.92E-04	2.96E-02
ENSG00000248161.5	*RP11-499E18.1*	2.93E-04	2.96E-02
ENSG00000167635.11	*ZNF146*	2.95E-04	2.97E-02
ENSG00000113734.17	*BNIP1*	2.98E-04	2.98E-02
ENSG00000178257.3	*PRM3*	3.08E-04	3.08E-02
ENSG00000166797.10	*FAM96A*	3.11E-04	3.09E-02
ENSG00000126215.13	*XRCC3*	3.29E-04	3.25E-02
ENSG00000215009.5	*ACSM4*	3.35E-04	3.30E-02
ENSG00000131435.12	*PDLIM4*	3.38E-04	3.31E-02
ENSG00000188186.10	*LAMTOR4*	3.41E-04	3.32E-02
ENSG00000168476.11	*REEP4*	3.49E-04	3.39E-02
ENSG00000231389.7	*HLA-DPA1*	3.56E-04	3.45E-02
ENSG00000221838.9	*AP4M1*	3.63E-04	3.49E-02
ENSG00000206503.12	*HLA-A*	3.81E-04	3.65E-02
ENSG00000187987.9	*ZSCAN23*	3.90E-04	3.72E-02
ENSG00000198040.10	*ZNF84*	3.94E-04	3.72E-02
ENSG00000140506.16	*LMAN1L*	3.94E-04	3.72E-02
ENSG00000228672.3	*PROB1*	3.95E-04	3.72E-02
ENSG00000259772.6	*RP11-16E12.2*	3.96E-04	3.72E-02
ENSG00000073605.18	*GSDMB*	4.05E-04	3.78E-02
ENSG00000153179.12	*RASSF3*	4.13E-04	3.84E-02

ENSG00000112706.11	*IMPG1*	4.25E-04	3.91E-02
ENSG00000109390.11	*NDUFC1*	4.27E-04	3.91E-02
ENSG00000021461.16	*CYP3A43*	4.27E-04	3.91E-02
ENSG00000225930.3	*DKFZP434L187*	4.29E-04	3.91E-02
ENSG00000122591.11	*FAM126A*	4.29E-04	3.91E-02
ENSG00000213578.5	*CPLX3*	4.31E-04	3.91E-02
ENSG00000161405.16	*IKZF3*	4.40E-04	3.98E-02
ENSG00000187626.8	*ZKSCAN4*	4.56E-04	4.10E-02
ENSG00000171695.10	*LKAAEAR1*	4.57E-04	4.10E-02
ENSG00000167083.6	*GNGT2*	4.62E-04	4.13E-02
ENSG00000163171.7	*CDC42EP3*	4.68E-04	4.15E-02
ENSG00000115541.10	*HSPE1*	4.71E-04	4.15E-02
ENSG00000173418.11	*NAA20*	4.71E-04	4.15E-02
ENSG00000110079.16	*MS4A4A*	4.74E-04	4.15E-02
ENSG00000154358.20	*OBSCN*	4.76E-04	4.15E-02
ENSG00000162913.9	*C1orf145*	4.76E-04	4.15E-02
ENSG00000164663.14	*USP49*	4.79E-04	4.16E-02
ENSG00000167914.11	*GSDMA*	4.83E-04	4.18E-02
ENSG00000120451.10	*SNX19*	4.87E-04	4.19E-02
ENSG00000155034.18	*FBXL18*	4.87E-04	4.19E-02
ENSG00000169242.11	*EFNA1*	4.94E-04	4.23E-02
ENSG00000177202.2	*SPACA4*	5.01E-04	4.27E-02
ENSG00000160505.15	*NLRP4*	5.04E-04	4.28E-02
ENSG00000164134.12	*NAA15*	5.07E-04	4.29E-02
ENSG00000132153.14	*DHX30*	5.10E-04	4.29E-02
ENSG00000168300.13	*PCMTD1*	5.11E-04	4.29E-02
ENSG00000100601.9	*ALKBH1*	5.19E-04	4.34E-02
ENSG00000072682.18	*P4HA2*	5.34E-04	4.45E-02
ENSG00000163374.19	*YY1AP1*	5.35E-04	4.45E-02
ENSG00000078018.19	*MAP2*	5.54E-04	4.58E-02
ENSG00000066923.17	*STAG3*	5.57E-04	4.59E-02
ENSG00000137265.14	*IRF4*	5.61E-04	4.60E-02
ENSG00000105559.11	*PLEKHA4*	5.67E-04	4.64E-02
ENSG00000057294.14	*PKP2*	5.69E-04	4.64E-02
ENSG00000079819.18	*EPB41L2*	5.80E-04	4.72E-02
ENSG00000110711.9	*AIP*	5.87E-04	4.75E-02
ENSG00000204410.14	*MSH5*	5.94E-04	4.79E-02

Supplementary Table S6. The 158 significant genes associated with susceptible COVID-19 identified by S-MultiXcan analysis based on 49 GTEx tissues

Gene	Gene name	P value	FDR
ENSG00000163817.15	*SLC6A20*	1.92E-171	4.29E-167
ENSG00000175164.13	*ABO*	2.37E-82	2.65E-78
ENSG00000173585.15	*CCR9*	5.27E-76	3.92E-72
ENSG00000172215.5	*CXCR6*	2.02E-36	1.13E-32

ENSG00000163818.16	*LZTFL1*	1.59E-35	7.10E-32
ENSG00000173578.7	*XCR1*	8.96E-30	3.34E-26
ENSG00000144802.11	*NFKBIZ*	2.22E-29	7.08E-26
ENSG00000144815.15	*NXPE3*	6.68E-28	1.87E-24
ENSG00000182504.10	*CEP97*	5.21E-27	1.29E-23
ENSG00000066422.4	*ZBTB11*	7.65E-25	1.71E-21
ENSG00000142002.16	*DPP9*	2.27E-23	4.61E-20
ENSG00000163820.14	*FYCO1*	4.83E-20	8.99E-17
ENSG00000160325.14	*CACFD1*	5.89E-20	1.01E-16
ENSG00000104804.7	*TULP2*	5.44E-17	8.68E-14
ENSG00000081154.11	*PCNP*	1.16E-16	1.73E-13
ENSG00000138468.15	*SENP7*	3.46E-16	4.83E-13
ENSG00000183625.14	*CCR3*	5.44E-16	7.15E-13
ENSG00000163823.3	*CCR1*	7.72E-16	9.58E-13
ENSG00000159110.19	*IFNAR2*	9.43E-16	1.11E-12
ENSG00000176920.11	*FUT2*	2.50E-15	2.79E-12
ENSG00000148297.15	*MED22*	1.03E-14	1.10E-11
ENSG00000243646.9	*IL10RB*	1.31E-13	1.33E-10
ENSG00000160326.13	*SLC2A6*	4.51E-13	4.38E-10
ENSG00000137166.14	*FOXP4*	6.10E-13	5.68E-10
ENSG00000105559.11	*PLEKHA4*	7.47E-13	6.67E-10
ENSG00000142233.11	*NTN5*	1.15E-12	9.88E-10
ENSG00000160323.18	*ADAMTS13*	3.38E-12	2.80E-09
ENSG00000087074.7	*PPP1R15A*	1.63E-11	1.30E-08
ENSG00000105538.9	*RASIP1*	3.09E-11	2.38E-08
ENSG00000104805.15	*NUCB1*	4.39E-11	3.27E-08
ENSG00000176909.11	*MAMSTR*	4.55E-11	3.28E-08
ENSG00000160791.13	*CCR5*	7.64E-11	5.33E-08
ENSG00000185499.16	*MUC1*	8.53E-11	5.77E-08
ENSG00000121807.5	*CCR2*	9.53E-11	6.26E-08
ENSG00000174951.10	*FUT1*	2.93E-10	1.87E-07
ENSG00000133661.15	*SFTPD*	9.84E-10	6.11E-07
ENSG00000182264.8	*IZUMO1*	1.13E-09	6.82E-07
ENSG00000148300.11	*REXO4*	1.92E-09	1.13E-06
ENSG00000163462.17	*TRIM46*	6.17E-09	3.53E-06
ENSG00000163463.11	*KRTCAP2*	1.51E-08	8.43E-06
ENSG00000121797.9	*CCRL2*	1.83E-08	9.97E-06
ENSG00000087076.8	*HSD17B14*	1.90E-08	1.01E-05
ENSG00000108342.12	*CSF3*	2.46E-08	1.28E-05
ENSG00000148303.16	*RPL7A*	2.55E-08	1.29E-05
ENSG00000131400.7	*NAPSA*	3.19E-08	1.58E-05
ENSG00000204536.13	*CCHCR1*	3.53E-08	1.71E-05
ENSG00000111331.12	*OAS3*	4.69E-08	2.23E-05
ENSG00000169231.13	*THBS3*	5.75E-08	2.68E-05
ENSG00000173171.14	*MTX1*	6.21E-08	2.83E-05
ENSG00000008838.19	*MED24*	1.16E-07	5.18E-05

ENSG00000167914.11	*GSDMA*	1.45E-07	6.35E-05
ENSG00000108344.14	*PSMD3*	1.65E-07	7.09E-05
ENSG00000267673.6	*FDX2*	1.71E-07	7.21E-05
ENSG00000204525.16	*HLA-C*	2.34E-07	9.68E-05
ENSG00000273102.1	*AP000569.9*	2.41E-07	9.79E-05
ENSG00000265799.1	*RP11-387H17.6*	2.47E-07	9.85E-05
ENSG00000231389.7	*HLA-DPA1*	3.07E-07	1.20E-04
ENSG00000233577.6	*RP3-462D8.2*	3.56E-07	1.37E-04
ENSG00000116604.17	*MEF2D*	3.76E-07	1.42E-04
ENSG00000114391.12	*RPL24*	5.53E-07	2.06E-04
ENSG00000177628.15	*GBA*	6.65E-07	2.44E-04
ENSG00000241837.6	*ATP5O*	7.14E-07	2.57E-04
ENSG00000081148.11	*IMPG2*	8.01E-07	2.80E-04
ENSG00000204538.3	*PSORS1C2*	8.02E-07	2.80E-04
ENSG00000105516.10	*DBP*	8.83E-07	3.03E-04
ENSG00000223865.10	*HLA-DPB1*	1.78E-06	6.02E-04
ENSG00000177202.2	*SPACA4*	2.07E-06	6.90E-04
ENSG00000089127.12	*OAS1*	2.11E-06	6.93E-04
ENSG00000106327.12	*TFR2*	2.19E-06	7.09E-04
ENSG00000160785.13	*SLC25A44*	2.29E-06	7.31E-04
ENSG00000160783.19	*PMF1*	2.49E-06	7.83E-04
ENSG00000106330.11	*MOSPD3*	2.80E-06	8.69E-04
ENSG00000077080.9	*ACTL6B*	3.26E-06	9.98E-04
ENSG00000243927.5	*MRPS6*	3.42E-06	1.03E-03
ENSG00000185361.8	*TNFAIP8L1*	3.48E-06	1.04E-03
ENSG00000169242.11	*EFNA1*	3.93E-06	1.16E-03
ENSG00000153922.10	*CHD1*	4.09E-06	1.19E-03
ENSG00000106333.12	*PCOLCE*	4.47E-06	1.28E-03
ENSG00000063180.8	*CA11*	5.05E-06	1.43E-03
ENSG00000143590.13	*EFNA3*	5.41E-06	1.51E-03
ENSG00000215375.6	*MYL5*	5.97E-06	1.65E-03
ENSG00000131355.14	*ADGRE3*	6.06E-06	1.65E-03
ENSG00000166250.11	*CLMP*	6.16E-06	1.66E-03
ENSG00000121691.4	*CAT*	7.14E-06	1.90E-03
ENSG00000146839.18	*ZAN*	7.54E-06	1.98E-03
ENSG00000105552.14	*BCAT2*	7.74E-06	2.01E-03
ENSG00000137310.11	*TCF19*	1.12E-05	2.88E-03
ENSG00000169241.17	*SLC50A1*	1.29E-05	3.27E-03
ENSG00000143537.13	*ADAM15*	1.36E-05	3.41E-03
ENSG00000204540.10	*PSORS1C1*	1.95E-05	4.84E-03
ENSG00000148291.9	*SURF2*	2.36E-05	5.79E-03
ENSG00000170498.8	*KISS1*	2.87E-05	6.97E-03
ENSG00000143627.17	*PKLR*	3.00E-05	7.21E-03
ENSG00000105523.3	*FAM83E*	3.04E-05	7.22E-03
ENSG00000012223.12	*LTF*	3.32E-05	7.81E-03
ENSG00000241186.9	*TDGF1*	3.50E-05	8.14E-03

ENSG00000131398.13	*KCNC3*	4.64E-05	1.07E-02
ENSG00000112787.12	*FBRSL1*	4.84E-05	1.10E-02
ENSG00000125503.12	*PPP1R12C*	6.21E-05	1.40E-02
ENSG00000101443.17	*WFDC2*	7.09E-05	1.57E-02
ENSG00000106018.13	*VIPR2*	7.11E-05	1.57E-02
ENSG00000166508.17	*MCM7*	7.15E-05	1.57E-02
ENSG00000242252.1	*BGLAP*	7.54E-05	1.64E-02
ENSG00000108370.16	*RGS9*	7.87E-05	1.69E-02
ENSG00000146830.9	*GIGYF1*	8.74E-05	1.86E-02
ENSG00000109111.14	*SUPT6H*	9.62E-05	2.03E-02
ENSG00000277186.1	*RP13-554M15.8*	1.06E-04	2.22E-02
ENSG00000106952.7	*TNFSF8*	1.08E-04	2.23E-02
ENSG00000204248.10	*COL11A2*	1.10E-04	2.26E-02
ENSG00000105610.4	*KLF1*	1.26E-04	2.55E-02
ENSG00000161847.13	*RAVER1*	1.27E-04	2.55E-02
ENSG00000272892.1	*RP11-57G10.8*	1.30E-04	2.59E-02
ENSG00000167491.17	*GATAD2A*	1.33E-04	2.60E-02
ENSG00000164683.16	*HEY1*	1.33E-04	2.60E-02
ENSG00000243364.7	*EFNA4*	1.36E-04	2.64E-02
ENSG00000164398.12	*ACSL6*	1.41E-04	2.70E-02
ENSG00000076662.9	*ICAM3*	1.41E-04	2.70E-02
ENSG00000126351.12	*THRA*	1.42E-04	2.70E-02
ENSG00000113140.10	*SPARC*	1.46E-04	2.72E-02
ENSG00000103148.15	*NPRL3*	1.47E-04	2.72E-02
ENSG00000131037.14	*EPS8L1*	1.47E-04	2.72E-02
ENSG00000238012.1	*AC114752.1*	1.50E-04	2.74E-02
ENSG00000229891.1	*LINC01315*	1.51E-04	2.74E-02
ENSG00000176715.15	*ACSF3*	1.52E-04	2.74E-02
ENSG00000074842.7	*MYDGF*	1.56E-04	2.78E-02
ENSG00000278463.1	*HIST1H2AB*	1.57E-04	2.78E-02
ENSG00000140262.17	*TCF12*	1.62E-04	2.85E-02
ENSG00000130427.2	*EPO*	1.63E-04	2.85E-02
ENSG00000235568.6	*NFAM1*	1.68E-04	2.92E-02
ENSG00000172057.9	*ORMDL3*	1.75E-04	3.00E-02
ENSG00000197343.10	*ZNF655*	1.76E-04	3.01E-02
ENSG00000086570.12	*FAT2*	1.80E-04	3.04E-02
ENSG00000104388.14	*RAB2A*	1.83E-04	3.07E-02
ENSG00000172354.9	*GNB2*	1.90E-04	3.17E-02
ENSG00000034713.7	*GABARAPL2*	1.94E-04	3.22E-02
ENSG00000131323.14	*TRAF3*	1.98E-04	3.25E-02
ENSG00000146463.11	*ZMYM4*	2.01E-04	3.27E-02
ENSG00000171885.13	*AQP4*	2.06E-04	3.33E-02
ENSG00000135374.9	*ELF5*	2.11E-04	3.38E-02
ENSG00000134917.9	*ADAMTS8*	2.12E-04	3.38E-02
ENSG00000132676.15	*DAP3*	2.25E-04	3.54E-02
ENSG00000220201.7	*ZGLP1*	2.25E-04	3.54E-02

ENSG00000167123.18	*CERCAM*	2.44E-04	3.80E-02
ENSG00000106261.16	*ZKSCAN1*	2.49E-04	3.86E-02
ENSG00000226846.1	*LINC00348*	2.63E-04	4.05E-02
ENSG00000226676.1	*RP11-589B3.6*	2.66E-04	4.06E-02
ENSG00000151461.19	*UPF2*	2.69E-04	4.08E-02
ENSG00000276410.3	*HIST1H2BB*	2.79E-04	4.20E-02
ENSG00000251396.6	*LINC01301*	2.80E-04	4.20E-02
ENSG00000105607.12	*GCDH*	2.93E-04	4.37E-02
ENSG00000124126.13	*PREX1*	3.10E-04	4.58E-02
ENSG00000180305.4	*WFDC10A*	3.15E-04	4.63E-02
ENSG00000186075.12	*ZPBP2*	3.26E-04	4.75E-02
ENSG00000204542.2	*C6orf15*	3.37E-04	4.85E-02
ENSG00000197935.6	*ZNF311*	3.37E-04	4.85E-02
ENSG00000267100.1	*ILF3-AS1*	3.40E-04	4.87E-02
ENSG00000185303.16	*SFTPA2*	3.43E-04	4.87E-02
ENSG00000184203.7	*PPP1R2*	3.48E-04	4.93E-02

Supplementary Table S7. The 67 common risk genes associated with three COVID-19 outcomes identified by S-MultiXcan analysis based on 49 GTEx tissues

Gene name	Susceptible COVID-19 (P-value)	Susceptible COVID-19 (FDR)	Hospitalized COVID-19 (P-value)	Hospitalized COVID-19 (FDR)	Very severe COVID-19 (P-value)	Very severe COVID-19 (FDR)
ABO	2.37E-82	2.65E-78	9.66E-23	1.27E-19	4.92E-13	4.07E-10
ACSF3	1.52E-04	2.74E-02	7.10E-07	1.96E-04	2.03E-09	9.86E-07
ACSL6	1.41E-04	2.70E-02	4.66E-06	1.05E-03	1.44E-06	3.70E-04
ADAM15	1.36E-05	3.41E-03	9.88E-12	7.12E-09	5.20E-13	4.15E-10
AP000569.9	2.41E-07	9.79E-05	5.59E-21	6.57E-18	2.50E-17	2.79E-14
ATP5O	7.14E-07	2.57E-04	4.95E-18	5.53E-15	2.45E-14	2.28E-11
C6orf15	3.37E-04	4.85E-02	6.91E-11	4.41E-08	2.56E-13	2.20E-10
CAT	7.14E-06	1.90E-03	3.20E-14	2.75E-11	9.25E-12	6.67E-09
CCHCR1	3.53E-08	1.71E-05	1.38E-10	7.71E-08	3.86E-11	2.54E-08
CCR1	7.72E-16	9.58E-13	5.25E-46	1.30E-42	2.85E-50	7.96E-47
CCR2	9.53E-11	6.26E-08	5.43E-38	9.33E-35	8.23E-45	1.67E-41
CCR3	5.44E-16	7.15E-13	6.93E-57	2.58E-53	7.31E-62	2.72E-58
CCR5	7.64E-11	5.33E-08	7.76E-39	1.44E-35	9.97E-47	2.23E-43
CCR9	5.27E-76	3.92E-72	3.04E-229	6.79E-225	6.50E-220	7.26E-216
CCRL2	1.83E-08	9.97E-06	5.12E-35	8.17E-32	7.73E-35	1.23E-31
CSF3	2.46E-08	1.28E-05	1.42E-04	1.80E-02	5.78E-05	8.67E-03
CXCR6	2.02E-36	1.13E-32	7.53E-114	5.61E-110	2.46E-118	1.83E-114
DPP9	2.27E-23	4.61E-20	1.11E-41	2.48E-38	5.70E-51	1.82E-47
EFNA1	3.93E-06	1.16E-03	4.94E-04	4.23E-02	2.52E-05	4.42E-03
EFNA3	5.41E-06	1.51E-03	2.67E-04	2.74E-02	1.01E-04	1.32E-02
EFNA4	1.36E-04	2.64E-02	4.83E-07	1.40E-04	5.09E-12	3.79E-09
ELF5	2.11E-04	3.38E-02	1.81E-14	1.62E-11	1.30E-09	6.75E-07

FBRSL1	4.84E-05	1.10E-02	2.24E-11	1.47E-08	7.15E-09	2.85E-06
FDX2	1.71E-07	7.21E-05	1.21E-10	6.93E-08	2.37E-09	1.08E-06
FOXP4	6.10E-13	5.68E-10	5.15E-26	7.67E-23	2.39E-17	2.79E-14
FUT2	2.50E-15	2.79E-12	1.42E-09	6.34E-07	9.04E-10	4.93E-07
FYCO1	4.83E-20	8.99E-17	2.54E-53	8.11E-50	2.62E-41	4.50E-38
GSDMA	1.45E-07	6.35E-05	4.83E-04	4.18E-02	4.52E-04	4.37E-02
HLA-C	2.34E-07	9.68E-05	9.51E-10	4.58E-07	6.13E-10	3.42E-07
ICAM3	1.41E-04	2.70E-02	4.54E-09	1.81E-06	1.41E-07	4.56E-05
IFNAR2	9.43E-16	1.11E-12	1.67E-50	4.66E-47	2.05E-47	5.09E-44
IL10RB	1.31E-13	1.33E-10	4.82E-41	9.79E-38	4.16E-42	7.74E-39
KCNC3	4.64E-05	1.07E-02	4.73E-09	1.82E-06	6.25E-06	1.32E-03
KRTCAP2	1.51E-08	8.43E-06	7.93E-11	4.92E-08	3.02E-09	1.32E-06
LINC01301	2.80E-04	4.20E-02	4.93E-08	1.67E-05	5.82E-07	1.63E-04
LTF	3.32E-05	7.81E-03	5.44E-14	4.50E-11	6.51E-14	5.82E-11
LZTFL1	1.59E-35	7.10E-32	5.65E-100	3.16E-96	1.64E-101	7.33E-98
MAMSTR	4.55E-11	3.28E-08	1.89E-04	2.16E-02	1.60E-08	5.86E-06
MCM7	7.15E-05	1.57E-02	3.18E-06	7.25E-04	7.28E-07	1.98E-04
MED24	1.16E-07	5.18E-05	1.10E-04	1.47E-02	6.91E-05	9.77E-03
MRPS6	3.42E-06	1.03E-03	2.59E-16	2.52E-13	3.52E-11	2.38E-08
MTX1	6.21E-08	2.83E-05	4.60E-22	5.71E-19	1.52E-27	2.26E-24
MUC1	8.53E-11	5.77E-08	2.21E-24	3.09E-21	5.20E-25	7.26E-22
MYDGF	1.56E-04	2.78E-02	2.26E-06	5.26E-04	1.86E-06	4.54E-04
NAPSA	3.19E-08	1.58E-05	4.33E-17	4.40E-14	2.36E-10	1.39E-07
NFKBIZ	2.22E-29	7.08E-26	6.81E-13	5.43E-10	1.08E-05	2.15E-03
NTN5	1.15E-12	9.88E-10	1.07E-06	2.81E-04	1.63E-08	5.87E-06
OAS1	2.11E-06	6.93E-04	9.67E-08	3.18E-05	4.10E-09	1.73E-06
OAS3	4.69E-08	2.23E-05	9.33E-11	5.63E-08	1.24E-07	4.07E-05
PLEKHA4	7.47E-13	6.67E-10	5.67E-04	4.64E-02	4.08E-04	4.09E-02
PSMD3	1.65E-07	7.09E-05	1.45E-05	2.70E-03	4.86E-06	1.05E-03
PSORS1C1	1.95E-05	4.84E-03	1.39E-05	2.63E-03	3.12E-07	9.17E-05
PSORS1C2	8.02E-07	2.80E-04	3.28E-08	1.16E-05	1.07E-09	5.69E-07
RAB2A	1.83E-04	3.07E-02	5.91E-08	1.97E-05	2.78E-07	8.28E-05
RASIP1	3.09E-11	2.38E-08	4.51E-05	7.20E-03	1.11E-08	4.28E-06
RAVER1	1.27E-04	2.55E-02	2.81E-09	1.16E-06	5.26E-07	1.51E-04
RP11-589B3.6	2.66E-04	4.06E-02	2.69E-07	8.12E-05	4.88E-08	1.65E-05
SFTPA2	3.43E-04	4.87E-02	4.27E-08	1.49E-05	4.29E-08	1.47E-05
SFTPD	9.84E-10	6.11E-07	3.46E-10	1.89E-07	1.94E-09	9.63E-07
SLC50A1	1.29E-05	3.27E-03	2.12E-11	1.44E-08	1.78E-14	1.73E-11
SLC6A20	1.92E-171	4.29E-167	2.30E-217	2.57E-213	7.16E-227	1.60E-222
TCF19	1.12E-05	2.88E-03	4.83E-10	2.51E-07	2.17E-09	1.01E-06
TNFAIP8L1	3.48E-06	1.04E-03	6.77E-09	2.52E-06	1.00E-12	7.70E-10
TRIM46	6.17E-09	3.53E-06	8.52E-12	6.34E-09	1.68E-19	2.08E-16
XCR1	8.96E-30	3.34E-26	1.53E-86	6.84E-83	1.81E-112	1.01E-108
ZGLP1	2.25E-04	3.54E-02	3.84E-10	2.04E-07	5.36E-09	2.22E-06
ZKSCAN1	2.49E-04	3.86E-02	2.26E-06	5.26E-04	2.40E-06	5.53E-04

Supplementary Table S8. The S-MultiXcan-identified 438 risk genes validated by S-PrediXcan analysis based on GTEx lung tissue

Gene Name	Susceptible COVID-19 (P-value)	Susceptible COVID-19 (FDR)	Hospitalized COVID-19 (P-value)	Hospitalized COVID-19 (FDR)	Very severe COVID-19 (P-value)	Very severe COVID-19 (FDR)
CCR9	NA	NA	NA	NA	NA	NA
SLC6A20	2.21E-17	8.06E-14	8.23E-08	6.67E-05	9.32E-08	7.77E-05
CXCR6	1.10E-16	3.21E-13	1.67E-83	2.44E-79	1.13E-87	1.65E-83
XCR1	4.61E-02	5.81E-01	3.06E-01	8.56E-01	7.59E-02	6.42E-01
LZTFL1	5.40E-02	6.05E-01	4.32E-01	9.02E-01	7.16E-01	9.68E-01
ABO	3.36E-52	4.90E-48	1.58E-14	2.88E-11	3.33E-05	1.24E-02
CCR3	5.47E-03	3.38E-01	1.94E-10	2.57E-07	8.87E-11	1.26E-07
FYCO1	2.72E-02	5.16E-01	2.39E-19	8.71E-16	8.27E-22	4.02E-18
DPP9	9.08E-02	6.99E-01	3.54E-01	8.76E-01	2.95E-02	5.29E-01
IFNAR2	1.16E-03	1.75E-01	1.93E-06	1.08E-03	1.94E-06	1.09E-03
CCR1	4.69E-01	9.25E-01	7.51E-03	2.98E-01	3.09E-04	6.09E-02
CCR5	2.00E-17	8.06E-14	2.66E-43	1.94E-39	3.69E-50	2.69E-46
CCR2	3.12E-04	6.81E-02	3.55E-02	5.22E-01	6.00E-02	6.26E-01
IL10RB	4.25E-09	5.63E-06	2.30E-18	6.68E-15	1.32E-18	3.85E-15
CCRL2	3.93E-02	5.58E-01	1.57E-04	3.42E-02	3.79E-05	1.38E-02
NFKBIZ	NA	NA	NA	NA	NA	NA
NXPE3	1.89E-12	3.06E-09	1.56E-03	1.45E-01	4.26E-03	2.82E-01
MTX1	4.72E-08	4.05E-05	8.75E-01	9.88E-01	6.63E-04	1.04E-01
CEP97	9.42E-28	6.87E-24	1.25E-11	1.82E-08	5.66E-05	1.79E-02
FOXP4	2.17E-15	4.52E-12	5.44E-30	2.64E-26	1.67E-19	6.09E-16
MUC1	7.36E-05	2.16E-02	6.18E-03	2.73E-01	1.60E-09	1.94E-06
ZBTB11	1.46E-08	1.64E-05	5.78E-04	8.43E-02	3.51E-02	5.42E-01
ASH1L	NA	NA	NA	NA	NA	NA
AP000569.9	NA	NA	NA	NA	NA	NA
CACFD1	2.05E-04	5.06E-02	7.67E-01	9.75E-01	9.60E-01	9.96E-01
TRIM46	NA	NA	NA	NA	NA	NA
ATP5O	3.09E-05	1.10E-02	2.75E-18	6.68E-15	4.07E-13	9.89E-10
NAPSA	8.14E-01	9.86E-01	5.30E-02	5.69E-01	8.50E-01	9.87E-01
TULP2	1.18E-13	2.15E-10	3.48E-04	5.76E-02	1.03E-03	1.40E-01
PCNP	2.13E-06	1.00E-03	6.77E-02	6.13E-01	2.15E-01	7.94E-01
MRPS6	1.40E-01	7.50E-01	9.26E-02	6.58E-01	5.49E-01	9.34E-01
SENP7	1.45E-06	7.56E-04	7.25E-03	2.95E-01	4.40E-01	8.98E-01
LINC02009	NA	NA	NA	NA	NA	NA
TYK2	3.70E-03	2.94E-01	8.75E-05	2.16E-02	2.80E-08	2.92E-05
FUT2	1.35E-16	3.28E-13	3.79E-10	4.25E-07	9.53E-11	1.26E-07
MED22	2.60E-04	6.02E-02	1.80E-02	4.21E-01	1.74E-02	4.65E-01
SLC50A1	2.07E-08	2.16E-05	7.27E-02	6.24E-01	8.21E-02	6.57E-01
ELF5	1.12E-05	4.46E-03	2.83E-15	5.90E-12	5.87E-11	9.51E-08
CAT	2.75E-03	2.57E-01	5.36E-05	1.56E-02	5.38E-05	1.74E-02
LTF	7.80E-01	9.82E-01	8.97E-01	9.89E-01	3.43E-01	8.69E-01
C6orf15	NA	NA	NA	NA	NA	NA

SLC2A6	NA	NA	NA	NA	NA	NA
ADAM15	3.78E-01	8.95E-01	1.83E-04	3.87E-02	8.13E-08	7.41E-05
PLEKHA4	1.57E-07	1.09E-04	1.69E-02	4.10E-01	2.64E-02	5.23E-01
TNFAIP8L1	5.69E-04	1.04E-01	4.44E-03	2.25E-01	4.11E-02	5.65E-01
NTN5	1.13E-05	4.46E-03	2.20E-07	1.53E-04	6.31E-06	2.97E-03
ATP11A	4.23E-01	9.09E-01	5.65E-01	9.36E-01	2.08E-01	7.89E-01
ADAMTS13	3.60E-02	5.45E-01	2.53E-01	8.26E-01	5.18E-01	9.22E-01
EFNA4	NA	NA	NA	NA	NA	NA
RP11-798G7.6	NA	NA	NA	NA	NA	NA
PPP1R15A	2.76E-08	2.68E-05	9.51E-03	3.24E-01	1.83E-02	4.71E-01
GON4L	NA	NA	NA	NA	NA	NA
FBRSL1	9.01E-01	9.93E-01	7.38E-02	6.26E-01	2.63E-02	5.23E-01
RASIP1	4.63E-09	5.63E-06	6.31E-04	8.85E-02	6.33E-08	6.15E-05
CCHCR1	4.84E-05	1.60E-02	1.90E-07	1.39E-04	1.39E-04	3.55E-02
NUCB1	1.22E-09	1.78E-06	7.73E-03	3.02E-01	1.03E-02	3.88E-01
MAMSTR	1.25E-01	7.37E-01	1.24E-01	7.05E-01	8.01E-03	3.70E-01
KRTCAP2	NA	NA	NA	NA	NA	NA
OAS3	2.10E-01	8.20E-01	1.70E-02	4.11E-01	7.16E-02	6.37E-01
FDX2	1.47E-02	4.37E-01	1.57E-01	7.45E-01	2.92E-02	5.29E-01
PDE4A	7.52E-01	9.77E-01	4.66E-01	9.13E-01	9.56E-01	9.95E-01
PLEKHM1	NA	NA	NA	NA	NA	NA
FUT1	1.21E-02	4.22E-01	2.55E-01	8.30E-01	5.75E-02	6.15E-01
SFTPD	3.38E-03	2.90E-01	1.30E-02	3.62E-01	5.25E-01	9.24E-01
ZGLP1	4.78E-03	3.27E-01	1.58E-03	1.45E-01	2.85E-02	5.28E-01
TCF19	9.72E-07	5.25E-04	9.44E-08	7.25E-05	3.90E-07	2.47E-04
C5orf56	8.38E-02	6.82E-01	2.09E-01	7.97E-01	1.35E-01	7.27E-01
HLA-C	7.54E-04	1.22E-01	2.29E-05	7.95E-03	6.04E-05	1.81E-02
ARHGAP27	2.69E-03	2.57E-01	2.26E-10	2.75E-07	3.57E-11	6.51E-08
SLC35B1	5.67E-01	9.48E-01	8.28E-01	9.85E-01	6.12E-01	9.46E-01
DDX39B	7.36E-01	9.75E-01	1.27E-02	3.60E-01	3.50E-01	8.71E-01
PSORS1C2	3.97E-06	1.81E-03	4.99E-05	1.55E-02	7.33E-06	3.33E-03
IZUMO1	2.38E-03	2.50E-01	3.00E-01	8.51E-01	2.41E-02	5.06E-01
FAM215B	3.11E-01	8.72E-01	5.45E-05	1.56E-02	1.30E-02	4.17E-01
REXO4	5.24E-03	3.35E-01	7.37E-01	9.72E-01	3.59E-01	8.75E-01
ACSF3	5.40E-01	9.43E-01	3.25E-02	5.11E-01	2.84E-04	5.73E-02
RAVER1	5.66E-05	1.76E-02	2.66E-05	9.02E-03	5.66E-04	9.86E-02
ICAM5	1.59E-01	7.79E-01	1.56E-01	7.42E-01	3.14E-03	2.41E-01
OAS1	3.07E-07	2.04E-04	8.22E-09	7.49E-06	4.75E-06	2.39E-03
ICAM3	1.11E-01	7.22E-01	3.81E-02	5.29E-01	2.37E-02	5.02E-01
ZCCHC4	1.10E-05	4.46E-03	2.27E-08	1.95E-05	6.09E-05	1.81E-02
KCNC3	1.39E-02	4.36E-01	8.91E-03	3.18E-01	2.79E-01	8.43E-01
NPNT	4.86E-01	9.32E-01	1.31E-02	3.63E-01	1.15E-02	4.00E-01
WNT3	1.09E-02	4.10E-01	1.81E-12	2.93E-09	1.25E-12	2.60E-09
DCST2	4.45E-03	3.16E-01	1.58E-01	7.46E-01	4.74E-01	9.09E-01
HLA-A	6.16E-02	6.27E-01	8.76E-01	9.88E-01	1.79E-02	4.67E-01
HSD17B14	1.28E-07	9.63E-05	1.58E-03	1.45E-01	2.10E-03	1.96E-01

PI4K2B	1.32E-01	7.40E-01	1.12E-01	6.90E-01	5.87E-03	3.27E-01
CSF3	7.39E-08	5.99E-05	9.81E-06	4.21E-03	3.17E-06	1.65E-03
RPL7A	5.43E-01	9.44E-01	4.86E-01	9.19E-01	8.02E-01	9.80E-01
SEMA6B	1.94E-01	8.15E-01	2.93E-01	8.51E-01	3.50E-01	8.71E-01
SFTPA2	1.29E-04	3.42E-02	4.32E-07	2.86E-04	9.60E-08	7.77E-05
CDH15	2.19E-02	4.98E-01	6.56E-06	3.09E-03	6.45E-04	1.03E-01
RP11-589B3.6	2.00E-02	4.80E-01	2.40E-03	1.82E-01	1.07E-06	6.50E-04
LINC01301	2.69E-05	9.81E-03	4.16E-09	4.04E-06	1.09E-07	7.95E-05
THBS3	4.15E-07	2.53E-04	5.91E-01	9.42E-01	9.03E-03	3.78E-01
RAB2A	2.69E-05	9.81E-03	4.16E-09	4.04E-06	1.09E-07	7.95E-05
SACM1L	8.29E-01	9.86E-01	5.70E-01	9.38E-01	5.25E-02	6.04E-01
IBA57-AS1	NA	NA	NA	NA	NA	NA
MED24	8.86E-02	6.96E-01	2.17E-01	8.02E-01	3.63E-01	8.75E-01
DPF2	2.53E-01	8.50E-01	4.97E-04	7.54E-02	7.27E-01	9.70E-01
GSDMA	1.32E-07	9.63E-05	6.28E-06	3.05E-03	5.70E-06	2.77E-03
PMVK	3.47E-02	5.39E-01	3.96E-01	8.92E-01	5.82E-02	6.16E-01
PSMD3	7.33E-01	9.75E-01	3.60E-01	8.77E-01	8.42E-01	9.85E-01
RP11-506M13.3	NA	NA	NA	NA	NA	NA
NUTM2E	9.64E-01	9.98E-01	1.30E-01	7.10E-01	7.74E-04	1.18E-01
CSK	1.73E-01	7.98E-01	3.25E-01	8.64E-01	5.60E-01	9.36E-01
RP4-535B20.1	NA	NA	NA	NA	NA	NA
MICB	1.23E-01	7.37E-01	4.24E-01	8.98E-01	1.96E-01	7.83E-01
RTP3	NA	NA	NA	NA	NA	NA
RP11-387H17.6	1.73E-06	8.66E-04	3.51E-03	2.11E-01	7.06E-03	3.49E-01
ARL17A	1.01E-01	7.15E-01	4.54E-02	5.55E-01	1.10E-01	6.96E-01
HLA-DPA1	3.98E-01	9.00E-01	8.19E-01	9.82E-01	7.82E-01	9.78E-01
PSORS1C1	4.59E-02	5.81E-01	4.48E-01	9.08E-01	4.10E-01	8.89E-01
RP3-462D8.2	NA	NA	NA	NA	NA	NA
MEF2D	NA	NA	NA	NA	NA	NA
ICAM1	NA	NA	NA	NA	NA	NA
RP11-11C20.3	3.29E-01	8.81E-01	2.82E-02	4.90E-01	1.09E-04	2.90E-02
RPL24	5.96E-07	3.40E-04	1.32E-04	2.96E-02	1.45E-03	1.69E-01
SLC22A4	8.96E-01	9.93E-01	4.65E-01	9.13E-01	8.97E-01	9.93E-01
GBA	1.28E-01	7.37E-01	2.14E-01	8.01E-01	4.98E-01	9.17E-01
MCM7	5.69E-01	9.49E-01	5.16E-01	9.26E-01	2.32E-01	8.07E-01
COQ10B	7.72E-01	9.81E-01	4.52E-03	2.25E-01	1.50E-01	7.49E-01
LTA	NA	NA	NA	NA	NA	NA
IMPG2	2.06E-01	8.18E-01	4.68E-01	9.14E-01	4.08E-01	8.88E-01
LRRC37A2	2.56E-01	8.53E-01	2.06E-05	7.51E-03	3.14E-03	2.41E-01
DBP	NA	NA	NA	NA	NA	NA
CLK3	5.33E-01	9.41E-01	7.08E-02	6.23E-01	7.29E-01	9.71E-01
ZNF3	9.16E-02	7.02E-01	9.15E-01	9.91E-01	6.86E-01	9.60E-01
ZNF778	1.82E-02	4.60E-01	6.47E-07	3.93E-04	7.95E-05	2.19E-02

PPP1R11	7.72E-02	6.74E-01	8.88E-03	3.18E-01	6.01E-05	1.81E-02
SLC5A3	6.92E-01	9.70E-01	7.57E-02	6.28E-01	1.79E-01	7.70E-01
CDSN	NA	NA	NA	NA	NA	NA
ACSL6	3.69E-03	2.94E-01	7.39E-05	2.00E-02	1.12E-02	3.99E-01
PGC	1.74E-01	7.98E-01	9.77E-02	6.63E-01	1.18E-01	7.05E-01
DSP	4.95E-02	5.92E-01	1.93E-03	1.63E-01	1.47E-07	1.02E-04
HLA-DQA2	8.05E-02	6.80E-01	2.79E-03	1.94E-01	1.83E-04	4.23E-02
IFNAR1	1.22E-01	7.37E-01	3.12E-05	1.01E-02	8.91E-06	3.82E-03
FOXA3	5.61E-01	9.48E-01	3.14E-03	2.00E-01	4.87E-02	5.92E-01
HLA-DPB1	8.22E-01	9.86E-01	3.13E-02	5.05E-01	1.84E-01	7.73E-01
DTX1	2.04E-01	8.18E-01	1.17E-01	6.97E-01	1.04E-02	3.90E-01
MYDGF	6.15E-02	6.27E-01	2.51E-01	8.26E-01	4.05E-01	8.87E-01
RP11-182L21.6	1.99E-01	8.15E-01	3.77E-02	5.29E-01	5.58E-01	9.36E-01
EIF4EBP2	1.82E-01	8.04E-01	9.02E-01	9.90E-01	6.58E-02	6.37E-01
ANAPC4	2.80E-02	5.16E-01	8.04E-05	2.02E-02	7.44E-04	1.14E-01
SLC22A31	4.84E-02	5.87E-01	2.32E-03	1.78E-01	3.38E-02	5.39E-01
SPACA4	NA	NA	NA	NA	NA	NA
TFR2	4.16E-07	2.53E-04	6.52E-04	8.89E-02	2.28E-03	2.07E-01
HLA-DRA	2.54E-02	5.10E-01	1.08E-01	6.83E-01	1.04E-01	6.86E-01
ZKSCAN1	4.25E-01	9.09E-01	3.82E-01	8.86E-01	6.71E-02	6.37E-01
SLC25A44	4.25E-06	1.88E-03	1.70E-03	1.49E-01	6.14E-04	1.02E-01
PMF1	5.69E-06	2.44E-03	1.46E-02	3.83E-01	6.69E-04	1.04E-01
MOSPD3	6.06E-07	3.40E-04	7.54E-04	9.77E-02	2.49E-03	2.15E-01
BYSL	7.89E-02	6.79E-01	2.74E-02	4.86E-01	6.16E-01	9.46E-01
CTA-384D8.35	4.72E-01	9.26E-01	8.38E-02	6.45E-01	8.15E-01	9.81E-01
ACTL6B	NA	NA	NA	NA	NA	NA
HIP1	2.75E-01	8.64E-01	4.68E-03	2.27E-01	2.13E-04	4.70E-02
EFNA1	4.51E-08	4.05E-05	4.61E-01	9.13E-01	1.15E-02	4.00E-01
CDC37	NA	NA	NA	NA	NA	NA
CHD1	NA	NA	NA	NA	NA	NA
PCOLCE	7.17E-01	9.73E-01	5.86E-02	5.87E-01	2.25E-01	8.04E-01
AC006007.1	NA	NA	NA	NA	NA	NA
CA11	4.91E-01	9.33E-01	3.31E-01	8.66E-01	4.96E-01	9.17E-01
FUBP1	3.13E-03	2.78E-01	5.33E-07	3.38E-04	2.44E-06	1.32E-03
SH3BP2	6.04E-02	6.23E-01	1.13E-02	3.43E-01	1.49E-01	7.47E-01
EFNA3	NA	NA	NA	NA	NA	NA
C2orf16	1.89E-02	4.65E-01	8.79E-07	5.13E-04	1.11E-03	1.44E-01
MYL5	8.14E-01	9.86E-01	1.32E-01	7.12E-01	2.44E-01	8.18E-01
ADGRE3	1.99E-01	8.15E-01	4.16E-01	8.98E-01	5.84E-01	9.41E-01
CLMP	1.28E-02	4.25E-01	2.02E-03	1.67E-01	9.59E-04	1.34E-01
HLA-F	1.61E-01	7.82E-01	2.51E-01	8.26E-01	4.93E-02	5.92E-01
GATS	NA	NA	NA	NA	NA	NA
FDPS	4.65E-01	9.23E-01	7.69E-01	9.75E-01	1.92E-02	4.77E-01
ZAN	NA	NA	NA	NA	NA	NA
TNFRSF4	3.25E-01	8.79E-01	4.01E-01	8.94E-01	2.51E-01	8.23E-01

BCAT2	1.78E-06	8.66E-04	3.73E-02	5.28E-01	1.37E-01	7.31E-01
CYP1A1	6.41E-04	1.08E-01	1.10E-05	4.46E-03	5.25E-02	6.04E-01
TOMM7	2.63E-01	8.59E-01	8.30E-04	1.01E-01	7.04E-05	2.05E-02
YIPF2	4.93E-01	9.33E-01	6.75E-01	9.59E-01	2.83E-02	5.27E-01
POU5F1	1.76E-01	7.98E-01	8.27E-01	9.85E-01	4.97E-02	5.92E-01
P4HA2	4.43E-01	9.15E-01	2.26E-01	8.09E-01	6.27E-01	9.48E-01
RP11-298J23.8	NA	NA	NA	NA	NA	NA
DNAJB4	1.42E-01	7.54E-01	6.64E-01	9.57E-01	2.91E-01	8.47E-01
NLRP4	NA	NA	NA	NA	NA	NA
FRS3	6.88E-02	6.55E-01	8.67E-01	9.88E-01	6.44E-01	9.51E-01
GPC2	NA	NA	NA	NA	NA	NA
AGRN	3.18E-01	8.75E-01	6.21E-01	9.46E-01	6.18E-01	9.46E-01
COPS6	2.35E-01	8.37E-01	4.42E-01	9.05E-01	5.95E-02	6.22E-01
ATF6B	3.96E-02	5.60E-01	1.45E-05	5.72E-03	4.03E-05	1.43E-02
GUK1	2.31E-01	8.34E-01	2.16E-02	4.50E-01	1.03E-02	3.88E-01
RP11-298J23.10	4.66E-01	9.23E-01	7.02E-01	9.66E-01	7.71E-01	9.77E-01
CLK2	8.23E-01	9.86E-01	6.25E-03	2.73E-01	1.69E-05	6.66E-03
RP13-554M15.8	1.59E-01	7.79E-01	3.01E-02	4.99E-01	4.47E-02	5.81E-01
AZGP1	NA	NA	NA	NA	NA	NA
HCN3	7.96E-01	9.86E-01	4.73E-03	2.27E-01	2.30E-05	8.82E-03
SURF2	3.77E-01	8.95E-01	9.68E-01	9.96E-01	6.54E-01	9.51E-01
JAK1	7.98E-03	3.82E-01	4.34E-06	2.18E-03	3.02E-07	2.00E-04
SCAMP3	NA	NA	NA	NA	NA	NA
LINC00304	5.47E-01	9.45E-01	1.38E-03	1.36E-01	2.45E-04	5.26E-02
ZBTB7B	NA	NA	NA	NA	NA	NA
ACAD8	7.26E-01	9.73E-01	1.01E-01	6.69E-01	3.76E-02	5.52E-01
HLA-DMB	8.09E-02	6.81E-01	9.18E-03	3.21E-01	4.87E-03	3.01E-01
TRIM15	5.84E-01	9.51E-01	3.42E-01	8.71E-01	3.13E-02	5.30E-01
RNF17	NA	NA	NA	NA	NA	NA
KISS1	2.66E-01	8.61E-01	5.95E-02	5.89E-01	1.21E-02	4.03E-01
ABCF1	3.45E-01	8.88E-01	6.75E-02	6.13E-01	8.51E-02	6.61E-01
BTNL2	7.64E-01	9.80E-01	7.04E-01	9.66E-01	8.43E-01	9.86E-01
PKLR	NA	NA	NA	NA	NA	NA
FAM83E	3.79E-04	7.67E-02	1.43E-02	3.78E-01	2.25E-02	5.00E-01
TIMM29	NA	NA	NA	NA	NA	NA
LINC02138	NA	NA	NA	NA	NA	NA
LTB	NA	NA	NA	NA	NA	NA
DDR1	5.30E-02	6.04E-01	3.37E-01	8.69E-01	2.86E-01	8.45E-01
TDGF1	2.82E-01	8.66E-01	3.51E-02	5.22E-01	8.97E-03	3.78E-01
RSPO2	8.60E-01	9.89E-01	8.87E-01	9.88E-01	2.35E-01	8.11E-01
SFTPA1	1.17E-03	1.75E-01	3.07E-06	1.66E-03	6.70E-09	7.51E-06
RIT1	2.10E-01	8.20E-01	6.88E-01	9.62E-01	9.89E-01	9.98E-01
NUTM2B	1.97E-01	8.15E-01	3.47E-02	5.22E-01	2.15E-02	4.90E-01
KIAA0319L	9.89E-03	3.97E-01	2.12E-05	7.54E-03	6.35E-04	1.03E-01

ANHX	NA	NA	NA	NA	NA	NA
C17orf78	NA	NA	NA	NA	NA	NA
C1orf35	NA	NA	NA	NA	NA	NA
RHOF	NA	NA	NA	NA	NA	NA
SYT11	6.74E-01	9.66E-01	1.98E-01	7.89E-01	4.67E-05	1.61E-02
PRR15L	8.38E-04	1.31E-01	2.17E-03	1.72E-01	5.11E-03	3.08E-01
STK19	2.85E-01	8.68E-01	1.64E-03	1.47E-01	7.32E-05	2.09E-02
NECAB3	8.47E-01	9.89E-01	3.36E-01	8.68E-01	2.38E-01	8.13E-01
CNPY4	1.06E-01	7.20E-01	5.61E-03	2.56E-01	9.41E-03	3.79E-01
IFNA6	NA	NA	NA	NA	NA	NA
DST	NA	NA	NA	NA	NA	NA
ABTB2	2.01E-02	4.81E-01	1.34E-04	2.96E-02	2.08E-04	4.66E-02
PRICKLE4	4.12E-01	9.05E-01	1.46E-01	7.30E-01	1.93E-01	7.80E-01
KLC1	7.18E-01	9.73E-01	7.40E-01	9.73E-01	9.15E-01	9.93E-01
SPPL2C	NA	NA	NA	NA	NA	NA
PBXIP1	NA	NA	NA	NA	NA	NA
PPP1R12C	NA	NA	NA	NA	NA	NA
MARS2	NA	NA	NA	NA	NA	NA
UBE2D4	9.54E-01	9.97E-01	2.93E-01	8.51E-01	2.99E-02	5.30E-01
MARCKSL1	NA	NA	NA	NA	NA	NA
APOPT1	7.64E-03	3.76E-01	8.13E-06	3.71E-03	1.40E-06	8.16E-04
DHDDS	5.69E-04	1.04E-01	5.10E-03	2.39E-01	1.58E-02	4.53E-01
NINJ2	NA	NA	NA	NA	NA	NA
WFDC2	NA	NA	NA	NA	NA	NA
VIPR2	1.17E-01	7.27E-01	9.23E-01	9.92E-01	9.41E-01	9.95E-01
GATA6-AS1	NA	NA	NA	NA	NA	NA
BMP1	4.28E-02	5.68E-01	4.14E-05	1.31E-02	2.75E-03	2.30E-01
HLA-B	6.65E-01	9.66E-01	6.55E-01	9.55E-01	2.90E-01	8.46E-01
BGLAP	5.22E-01	9.40E-01	9.63E-01	9.95E-01	8.97E-01	9.93E-01
KANSL1	NA	NA	NA	NA	NA	NA
NELFE	4.60E-01	9.20E-01	6.75E-01	9.59E-01	5.38E-01	9.29E-01
RGS9	NA	NA	NA	NA	NA	NA
SPEF2	6.02E-01	9.53E-01	5.47E-01	9.32E-01	2.40E-01	8.14E-01
CTD-3010D24.3	NA	NA	NA	NA	NA	NA
ZSCAN21	9.36E-02	7.06E-01	1.45E-03	1.39E-01	9.73E-02	6.74E-01
CAMSAP2	4.12E-01	9.05E-01	4.19E-01	8.98E-01	2.65E-01	8.34E-01
UBE2L6	3.67E-01	8.92E-01	1.38E-01	7.20E-01	2.09E-01	7.89E-01
GIGYF1	6.17E-03	3.48E-01	8.63E-01	9.88E-01	7.39E-02	6.39E-01
GOLGA8K	NA	NA	NA	NA	NA	NA
ZCCHC24	4.37E-01	9.12E-01	7.24E-01	9.71E-01	5.41E-01	9.31E-01
TFAP2E	1.15E-01	7.27E-01	6.44E-04	8.89E-02	2.47E-03	2.15E-01
CYP3A43	7.53E-01	9.77E-01	8.47E-01	9.87E-01	2.29E-01	8.06E-01
TCF7L2	5.95E-01	9.53E-01	8.31E-01	9.85E-01	1.48E-01	7.47E-01
SUPT6H	NA	NA	NA	NA	NA	NA
SYMPK	8.10E-01	9.86E-01	8.03E-01	9.80E-01	8.88E-01	9.91E-01
DDAH2	6.20E-01	9.55E-01	3.00E-01	8.51E-01	8.89E-02	6.66E-01

AATK-AS1	NA	NA	NA	NA	NA	NA
CREBRF	NA	NA	NA	NA	NA	NA
C1orf56	3.14E-01	8.72E-01	8.66E-02	6.51E-01	8.06E-03	3.70E-01
MSTO1	NA	NA	NA	NA	NA	NA
ANXA11	5.18E-01	9.40E-01	6.95E-01	9.66E-01	5.89E-01	9.41E-01
TNFSF8	NA	NA	NA	NA	NA	NA
GAL3ST4	2.98E-01	8.69E-01	1.09E-01	6.84E-01	2.57E-02	5.20E-01
COL11A2	4.30E-01	9.10E-01	8.87E-01	9.88E-01	6.09E-01	9.45E-01
SPOP	2.11E-01	8.22E-01	1.52E-01	7.35E-01	4.05E-01	8.87E-01
PLSCR2	8.53E-01	9.89E-01	2.28E-01	8.10E-01	1.13E-01	7.00E-01
KLF1	8.24E-03	3.85E-01	2.48E-01	8.25E-01	7.99E-01	9.80E-01
ARL17B	3.11E-01	8.72E-01	5.45E-05	1.56E-02	1.30E-02	4.17E-01
C6orf48	3.13E-01	8.72E-01	2.33E-01	8.13E-01	7.35E-01	9.72E-01
RPP21	2.09E-01	8.19E-01	8.25E-01	9.84E-01	4.58E-01	9.05E-01
RP11-57G10.8	NA	NA	NA	NA	NA	NA
YLPM1	6.58E-01	9.65E-01	6.37E-03	2.77E-01	7.54E-06	3.33E-03
GATAD2A	3.97E-01	9.00E-01	6.71E-01	9.58E-01	5.47E-01	9.33E-01
OAS2	4.13E-01	9.05E-01	1.35E-02	3.67E-01	5.85E-02	6.17E-01
HEY1	NA	NA	NA	NA	NA	NA
YY1AP1	8.77E-01	9.92E-01	1.33E-01	7.13E-01	1.32E-03	1.56E-01
FCHSD2	1.98E-01	8.15E-01	8.77E-01	9.88E-01	3.73E-01	8.76E-01
MAPT	NA	NA	NA	NA	NA	NA
GABARAPL2	3.65E-01	8.92E-01	2.35E-03	1.79E-01	1.61E-01	7.59E-01
CYP4B1	1.58E-01	7.78E-01	3.50E-01	8.75E-01	3.19E-01	8.60E-01
RP11-91I20.3	NA	NA	NA	NA	NA	NA
THRA	7.23E-04	1.19E-01	2.20E-03	1.72E-01	2.43E-03	2.14E-01
EDC3	NA	NA	NA	NA	NA	NA
SPARC	9.11E-02	7.00E-01	2.79E-01	8.46E-01	9.54E-01	9.95E-01
NPRL3	1.84E-01	8.05E-01	1.64E-01	7.56E-01	6.83E-01	9.59E-01
EPS8L1	1.39E-03	1.89E-01	4.10E-02	5.44E-01	7.37E-01	9.72E-01
CFB	NA	NA	NA	NA	NA	NA
AC114752.1	NA	NA	NA	NA	NA	NA
CCDC18	1.82E-01	8.04E-01	2.59E-01	8.32E-01	1.90E-02	4.76E-01
LINC01315	1.11E-02	4.12E-01	9.02E-01	9.90E-01	6.75E-01	9.56E-01
NCOR1	2.43E-02	5.08E-01	5.63E-04	8.38E-02	2.29E-03	2.07E-01
SNX1	5.98E-01	9.53E-01	7.77E-02	6.32E-01	9.26E-02	6.71E-01
DNAH8	NA	NA	NA	NA	NA	NA
RAVER2	6.55E-01	9.64E-01	2.16E-01	8.02E-01	9.64E-01	9.96E-01
HIST1H2AB	NA	NA	NA	NA	NA	NA
SQRDL	8.61E-02	6.88E-01	1.98E-04	4.12E-02	4.01E-02	5.62E-01
FNIP1	NA	NA	NA	NA	NA	NA
TCF12	1.43E-04	3.71E-02	1.84E-01	7.72E-01	2.51E-01	8.23E-01
EPO	NA	NA	NA	NA	NA	NA
CTC-296K1.3	2.92E-02	5.17E-01	8.52E-02	6.49E-01	1.86E-01	7.75E-01

LAMTOR4	2.74E-01	8.64E-01	3.75E-01	8.82E-01	8.08E-01	9.80E-01
CTB-50L17.16	NA	NA	NA	NA	NA	NA
DONSON	4.82E-01	9.30E-01	1.24E-02	3.57E-01	4.33E-01	8.96E-01
ZPBP2	NA	NA	NA	NA	NA	NA
NFAM1	NA	NA	NA	NA	NA	NA
NCDN	6.41E-02	6.37E-01	7.33E-01	9.71E-01	8.23E-01	9.81E-01
ORMDL3	5.47E-05	1.73E-02	2.03E-05	7.51E-03	1.92E-03	1.94E-01
ZNF655	7.85E-01	9.83E-01	9.33E-02	6.59E-01	3.44E-01	8.70E-01
IBA57	5.78E-02	6.19E-01	7.90E-04	9.92E-02	3.05E-02	5.30E-01
CALR	7.78E-01	9.82E-01	7.45E-01	9.74E-01	8.99E-01	9.93E-01
CPOX	3.88E-03	2.98E-01	1.49E-01	7.34E-01	6.01E-02	6.26E-01
FAT2	1.27E-01	7.37E-01	3.15E-02	5.06E-01	6.35E-02	6.36E-01
ST3GAL3	9.63E-01	9.98E-01	2.41E-02	4.66E-01	1.71E-02	4.62E-01
DNAJB14	9.05E-01	9.94E-01	4.08E-01	8.97E-01	8.88E-01	9.91E-01
LINC01565	6.70E-01	9.66E-01	5.63E-03	2.56E-01	1.13E-01	7.00E-01
SNRPD2	1.65E-01	7.89E-01	7.15E-02	6.24E-01	5.82E-01	9.41E-01
GNB2	1.53E-02	4.38E-01	2.60E-01	8.34E-01	2.41E-01	8.14E-01
TSACC	1.84E-01	8.04E-01	8.55E-01	9.87E-01	9.83E-03	3.85E-01
DCST1	NA	NA	NA	NA	NA	NA
6-Mar	NA	NA	NA	NA	NA	NA
TRAF3	2.05E-03	2.34E-01	1.47E-01	7.31E-01	1.10E-02	3.97E-01
ZMYM4	NA	NA	NA	NA	NA	NA
AQP4	7.96E-01	9.86E-01	4.12E-01	8.97E-01	4.32E-02	5.78E-01
ARID3B	NA	NA	NA	NA	NA	NA
AP1M2	9.85E-02	7.15E-01	9.49E-01	9.95E-01	8.13E-01	9.80E-01
FAM189B	7.15E-01	9.73E-01	6.00E-01	9.43E-01	1.94E-01	7.81E-01
ADAMTS8	6.39E-01	9.59E-01	6.02E-01	9.43E-01	7.20E-01	9.69E-01
MICA	8.65E-01	9.90E-01	1.28E-02	3.60E-01	3.11E-02	5.30E-01
PRSS33	NA	NA	NA	NA	NA	NA
DAP3	8.77E-01	9.92E-01	1.33E-01	7.13E-01	1.32E-03	1.56E-01
LINC01149	NA	NA	NA	NA	NA	NA
TAF6	3.94E-01	9.00E-01	1.02E-01	6.69E-01	7.68E-01	9.77E-01
GPANK1	1.96E-01	8.15E-01	2.00E-01	7.93E-01	3.55E-02	5.42E-01
LINC00310	4.64E-01	9.21E-01	2.65E-02	4.81E-01	7.95E-02	6.53E-01
RP11-503G7.2	NA	NA	NA	NA	NA	NA
FRYL	3.13E-02	5.27E-01	3.43E-04	5.76E-02	8.70E-03	3.77E-01
E2F1	7.04E-02	6.59E-01	4.63E-02	5.56E-01	4.11E-01	8.89E-01
PRRT1	1.03E-01	7.16E-01	1.28E-02	3.60E-01	2.92E-02	5.29E-01
IRF7	1.55E-02	4.38E-01	2.17E-02	4.50E-01	8.51E-03	3.76E-01
LINC00857	NA	NA	NA	NA	NA	NA
CERCAM	7.76E-03	3.80E-01	5.44E-01	9.31E-01	5.82E-01	9.41E-01
ZSWIM7	1.27E-02	4.24E-01	5.89E-05	1.65E-02	2.51E-04	5.31E-02
ADRA1A	1.45E-01	7.58E-01	3.04E-01	8.56E-01	4.68E-01	9.08E-01
NPY4R	NA	NA	NA	NA	NA	NA
TRMT61B	3.04E-03	2.76E-01	7.86E-05	2.02E-02	9.96E-02	6.79E-01

GJC2	2.73E-01	8.63E-01	2.61E-02	4.79E-01	1.97E-03	1.94E-01
PSMB2	2.34E-01	8.36E-01	3.21E-03	2.02E-01	5.71E-02	6.14E-01
PTGFR	9.06E-01	9.94E-01	8.78E-01	9.88E-01	8.91E-01	9.92E-01
VPS29	5.69E-01	9.49E-01	6.13E-01	9.43E-01	2.91E-01	8.46E-01
LINC00348	NA	NA	NA	NA	NA	NA
BRD2	5.89E-02	6.21E-01	4.76E-01	9.17E-01	6.64E-01	9.54E-01
UPF2	1.08E-01	7.20E-01	9.55E-01	9.95E-01	8.49E-02	6.61E-01
CHCHD7	8.30E-02	6.82E-01	7.51E-02	6.26E-01	1.23E-03	1.52E-01
HIST1H2BB	NA	NA	NA	NA	NA	NA
HSPE1	9.47E-01	9.97E-01	1.31E-01	7.11E-01	3.52E-03	2.52E-01
RP11-499E18.1	5.49E-01	9.45E-01	1.34E-01	7.14E-01	3.81E-01	8.79E-01
GCDH	7.57E-01	9.78E-01	2.15E-01	8.01E-01	4.77E-01	9.10E-01
ZNF146	6.74E-01	9.66E-01	5.23E-02	5.67E-01	9.69E-04	1.34E-01
CYP2C19	5.19E-01	9.40E-01	3.52E-01	8.75E-01	4.89E-02	5.92E-01
BNIP1	2.23E-01	8.30E-01	3.93E-02	5.38E-01	8.37E-01	9.84E-01
DR1	2.60E-03	2.57E-01	4.04E-03	2.25E-01	3.21E-04	6.23E-02
SUCLG2	3.47E-01	8.89E-01	3.85E-01	8.88E-01	8.69E-01	9.89E-01
TADA1	5.63E-01	9.48E-01	7.24E-02	6.24E-01	1.99E-01	7.84E-01
PRM3	NA	NA	NA	NA	NA	NA
PREX1	2.46E-01	8.47E-01	4.60E-01	9.13E-01	8.30E-01	9.83E-01
FAM96A	NA	NA	NA	NA	NA	NA
WFDC10A	NA	NA	NA	NA	NA	NA
AIP	2.95E-02	5.17E-01	4.93E-04	7.54E-02	1.83E-04	4.23E-02
PNRC1	4.25E-01	9.09E-01	2.96E-01	8.51E-01	1.15E-02	4.00E-01
XRCC3	2.81E-02	5.16E-01	3.55E-04	5.81E-02	2.15E-03	1.98E-01
LPCAT2	NA	NA	NA	NA	NA	NA
ATG16L2	1.67E-01	7.92E-01	3.65E-03	2.16E-01	1.57E-05	6.54E-03
ACSM4	7.42E-01	9.76E-01	2.25E-01	8.08E-01	8.55E-02	6.62E-01
ZNF311	5.24E-02	6.03E-01	5.44E-01	9.31E-01	6.43E-01	9.51E-01
HNMT	1.20E-01	7.34E-01	1.79E-01	7.68E-01	3.84E-01	8.80E-01
PDLIM4	7.95E-01	9.86E-01	9.35E-01	9.93E-01	7.51E-01	9.75E-01
ILF3-AS1	3.68E-02	5.48E-01	4.88E-01	9.19E-01	4.23E-01	8.92E-01
PPP1R2	4.01E-01	9.00E-01	9.50E-01	9.95E-01	8.63E-01	9.88E-01
REEP4	4.47E-01	9.17E-01	6.98E-01	9.66E-01	9.97E-01	9.99E-01
RPN1	8.92E-01	9.93E-01	9.43E-02	6.59E-01	3.64E-01	8.75E-01
LPIN3	9.74E-03	3.95E-01	4.13E-02	5.45E-01	5.01E-02	5.95E-01
ARHGEF38	NA	NA	NA	NA	NA	NA
AP4M1	2.52E-02	5.10E-01	8.54E-04	1.03E-01	3.41E-02	5.40E-01
ZSCAN23	6.21E-01	9.55E-01	1.02E-02	3.32E-01	3.89E-02	5.58E-01
ZNF84	1.57E-01	7.75E-01	8.96E-02	6.54E-01	7.44E-02	6.39E-01
LMAN1L	NA	NA	NA	NA	NA	NA
PROB1	3.71E-03	2.94E-01	2.16E-02	4.50E-01	1.86E-03	1.94E-01
RP11-16E12.2	3.40E-01	8.86E-01	5.69E-02	5.80E-01	6.58E-02	6.37E-01
BMP8A	5.02E-01	9.35E-01	2.78E-01	8.46E-01	1.00E+00	1.00E+00
GSDMB	1.81E-04	4.55E-02	4.28E-06	2.18E-03	8.35E-04	1.23E-01

UNC5C	NA	NA	NA	NA	NA	NA
RASSF3	7.05E-01	9.70E-01	1.82E-01	7.70E-01	3.23E-01	8.62E-01
IMPG1	7.84E-01	9.83E-01	9.65E-01	9.96E-01	6.13E-01	9.46E-01
NDUFC1	6.86E-01	9.70E-01	4.73E-01	9.15E-01	8.64E-01	9.88E-01
DKFZP434L187	NA	NA	NA	NA	NA	NA
FAM126A	1.66E-01	7.90E-01	7.61E-01	9.75E-01	6.36E-01	9.50E-01
CPLX3	3.11E-01	8.72E-01	2.43E-04	4.82E-02	7.23E-03	3.50E-01
IKZF3	6.26E-04	1.08E-01	1.24E-04	2.88E-02	1.99E-03	1.94E-01
KIAA0907	3.10E-01	8.72E-01	7.86E-01	9.77E-01	2.03E-03	1.94E-01
ZKSCAN4	3.64E-02	5.47E-01	4.34E-03	2.25E-01	1.07E-01	6.90E-01
LKAAEAR1	1.65E-01	7.89E-01	1.38E-03	1.36E-01	2.03E-01	7.87E-01
DYNC1H1	5.52E-02	6.10E-01	1.17E-01	6.97E-01	4.52E-02	5.81E-01
GNGT2	9.03E-02	6.98E-01	2.11E-03	1.71E-01	3.78E-02	5.53E-01
CDC42EP3	NA	NA	NA	NA	NA	NA
NAA20	NA	NA	NA	NA	NA	NA
MS4A4A	4.08E-01	9.04E-01	1.16E-02	3.47E-01	6.47E-02	6.37E-01
OBSCN	NA	NA	NA	NA	NA	NA
C1orf145	NA	NA	NA	NA	NA	NA
CDH16	NA	NA	NA	NA	NA	NA
USP49	1.36E-02	4.33E-01	2.09E-04	4.24E-02	3.32E-01	8.64E-01
SNX19	1.14E-02	4.17E-01	7.74E-04	9.81E-02	2.43E-02	5.08E-01
FBXL18	1.80E-01	8.01E-01	1.88E-01	7.75E-01	9.29E-01	9.95E-01
VWA7	7.28E-01	9.73E-01	3.31E-02	5.14E-01	3.14E-02	5.30E-01
NAA15	7.16E-01	9.73E-01	2.46E-03	1.82E-01	6.69E-02	6.37E-01
DHX30	NA	NA	NA	NA	NA	NA
PCMTD1	7.90E-01	9.84E-01	1.40E-02	3.74E-01	5.51E-03	3.25E-01
HLA-DOA	4.25E-01	9.09E-01	4.73E-01	9.15E-01	7.79E-01	9.78E-01
RFTN2	NA	NA	NA	NA	NA	NA
ALKBH1	4.89E-01	9.32E-01	1.88E-02	4.27E-01	6.08E-01	9.45E-01
LDLR	NA	NA	NA	NA	NA	NA
DSCAML1	7.97E-01	9.86E-01	3.49E-01	8.74E-01	1.65E-01	7.60E-01
LCTL	9.66E-01	9.98E-01	6.64E-02	6.10E-01	1.84E-02	4.71E-01
HINT1	1.02E-01	7.15E-01	1.88E-01	7.76E-01	1.92E-02	4.77E-01
MAP2	2.00E-02	4.80E-01	3.18E-01	8.60E-01	4.88E-01	9.14E-01
STAG3	8.31E-01	9.86E-01	9.67E-01	9.96E-01	3.33E-01	8.66E-01
IRF4	3.56E-02	5.43E-01	3.15E-04	5.51E-02	5.89E-02	6.19E-01
PKP2	2.78E-01	8.64E-01	5.17E-01	9.26E-01	1.96E-01	7.83E-01
EPB41L2	6.37E-01	9.58E-01	8.85E-01	9.88E-01	2.39E-01	8.13E-01
MSH5	NA	NA	NA	NA	NA	NA

Note: NA represents not applicable.

Supplementary Table S9. The S-MultiXcan-identified 438 risk genes validated by S-PrediXcan analysis based on the GTEx whole blood

Gene Name	Susceptible COVID-19 (P-value)	Susceptible COVID-19 (FDR)	Hospitalized COVID-19 (P-value)	Hospitalized COVID-19 (FDR)	Very severe COVID-19 (P-value)	Very severe COVID-19 (FDR)

CCR9	5.72E-71	6.99E-67	9.15E-216	1.12E-211	5.26E-217	6.43E-213
SLC6A20	NA	NA	NA	NA	NA	NA
CXCR6	NA	NA	NA	NA	NA	NA
XCR1	1.98E-04	7.14E-02	9.90E-09	2.02E-05	4.80E-08	5.86E-05
LZTFL1	5.19E-03	3.50E-01	2.45E-02	4.57E-01	1.34E-02	4.36E-01
ABO	3.32E-16	2.03E-12	5.61E-05	1.52E-02	6.07E-04	1.03E-01
CCR3	1.45E-01	7.54E-01	4.99E-02	5.85E-01	2.58E-02	5.11E-01
FYCO1	3.42E-01	8.86E-01	5.35E-05	1.49E-02	7.88E-04	1.14E-01
DPP9	1.49E-05	9.59E-03	4.29E-07	4.77E-04	1.80E-09	3.66E-06
IFNAR2	7.05E-02	6.45E-01	6.61E-05	1.72E-02	2.66E-03	2.29E-01
CCR1	2.92E-03	2.75E-01	1.84E-01	7.85E-01	2.74E-01	8.44E-01
CCR5	6.78E-02	6.39E-01	1.59E-01	7.60E-01	1.66E-01	7.83E-01
CCR2	1.19E-04	5.21E-02	3.47E-01	8.77E-01	6.40E-01	9.55E-01
IL10RB	6.71E-01	9.61E-01	4.23E-02	5.43E-01	9.89E-04	1.28E-01
CCRL2	NA	NA	NA	NA	NA	NA
NFKBIZ	NA	NA	NA	NA	NA	NA
NXPE3	6.49E-14	2.64E-10	7.25E-04	8.44E-02	7.95E-04	1.14E-01
MTX1	4.66E-08	5.77E-05	4.32E-02	5.48E-01	7.42E-01	9.70E-01
CEP97	4.09E-01	9.11E-01	3.52E-01	8.80E-01	5.87E-01	9.49E-01
FOXP4	NA	NA	NA	NA	NA	NA
MUC1	5.96E-01	9.57E-01	7.00E-10	2.14E-06	8.86E-15	5.41E-11
ZBTB11	NA	NA	NA	NA	NA	NA
ASH1L	NA	NA	NA	NA	NA	NA
AP000569.9	NA	NA	NA	NA	NA	NA
CACFD1	6.70E-01	9.61E-01	7.06E-01	9.68E-01	8.77E-01	9.84E-01
TRIM46	NA	NA	NA	NA	NA	NA
ATP5O	4.25E-01	9.15E-01	2.91E-06	1.87E-03	3.56E-04	7.38E-02
NAPSA	1.06E-08	2.16E-05	2.17E-18	1.33E-14	7.17E-11	2.19E-07
TULP2	NA	NA	NA	NA	NA	NA
PCNP	2.19E-03	2.46E-01	2.51E-03	1.62E-01	2.87E-01	8.48E-01
MRPS6	8.40E-01	9.84E-01	6.28E-01	9.49E-01	8.80E-01	9.84E-01
SENP7	2.62E-10	8.01E-07	5.24E-04	6.97E-02	1.63E-01	7.83E-01
LINC02009	2.94E-02	5.31E-01	2.62E-03	1.67E-01	1.46E-02	4.43E-01
TYK2	2.76E-04	8.65E-02	2.11E-08	3.68E-05	2.79E-12	1.14E-08
FUT2	NA	NA	NA	NA	NA	NA
MED22	5.35E-01	9.46E-01	4.51E-01	9.17E-01	2.97E-01	8.59E-01
SLC50A1	8.99E-02	6.79E-01	8.75E-01	9.88E-01	3.40E-01	8.73E-01
ELF5	NA	NA	NA	NA	NA	NA
CAT	3.95E-02	5.64E-01	3.90E-03	2.10E-01	4.40E-03	2.76E-01
LTF	NA	NA	NA	NA	NA	NA
C6orf15	NA	NA	NA	NA	NA	NA
SLC2A6	1.68E-08	2.93E-05	4.03E-03	2.13E-01	3.39E-02	5.45E-01
ADAM15	4.81E-04	1.20E-01	1.47E-04	2.85E-02	4.95E-03	2.88E-01
PLEKHA4	3.18E-01	8.76E-01	2.41E-01	8.21E-01	2.06E-01	8.13E-01
TNFAIP8L1	8.25E-03	4.09E-01	3.07E-02	4.95E-01	1.00E-01	7.08E-01
NTN5	2.05E-05	1.21E-02	5.06E-03	2.34E-01	2.29E-03	2.16E-01

ATP11A	4.78E-03	3.43E-01	1.32E-06	1.01E-03	2.24E-04	5.17E-02
ADAMTS13	2.51E-02	5.24E-01	9.73E-03	3.23E-01	6.63E-01	9.59E-01
EFNA4	NA	NA	NA	NA	NA	NA
RP11-798G7.6	NA	NA	NA	NA	NA	NA
PPP1R15A	2.36E-01	8.31E-01	1.69E-01	7.70E-01	1.87E-01	8.03E-01
GON4L	NA	NA	NA	NA	NA	NA
FBRSL1	9.72E-03	4.23E-01	1.91E-03	1.44E-01	2.84E-01	8.47E-01
RASIP1	4.63E-09	1.13E-05	6.31E-04	7.64E-02	6.33E-08	7.03E-05
CCHCR1	1.80E-06	1.69E-03	3.58E-07	4.38E-04	8.89E-05	2.78E-02
NUCB1	2.91E-04	8.88E-02	3.30E-01	8.70E-01	8.92E-02	6.97E-01
MAMSTR	2.24E-08	3.42E-05	8.89E-05	2.03E-02	2.49E-08	3.38E-05
KRTCAP2	NA	NA	NA	NA	NA	NA
OAS3	1.36E-05	9.24E-03	2.67E-09	6.53E-06	4.03E-06	2.46E-03
FDX2	3.86E-01	9.05E-01	1.94E-01	7.94E-01	6.50E-01	9.57E-01
PDE4A	9.00E-01	9.90E-01	3.68E-01	8.88E-01	7.94E-01	9.74E-01
PLEKHM1	NA	NA	NA	NA	NA	NA
FUT1	NA	NA	NA	NA	NA	NA
SFTPD	1.82E-01	7.92E-01	1.46E-04	2.85E-02	3.75E-07	2.86E-04
ZGLP1	4.11E-03	3.22E-01	4.38E-05	1.43E-02	6.89E-04	1.09E-01
TCF19	3.08E-06	2.45E-03	2.42E-07	3.70E-04	2.88E-05	1.13E-02
C5orf56	3.21E-03	2.84E-01	9.75E-04	9.54E-02	7.71E-03	3.59E-01
HLA-C	4.11E-06	2.96E-03	2.79E-07	3.79E-04	4.40E-09	6.72E-06
ARHGAP27	1.77E-02	4.97E-01	5.60E-06	3.11E-03	4.52E-02	5.99E-01
SLC35B1	5.67E-01	9.54E-01	8.28E-01	9.84E-01	6.12E-01	9.52E-01
DDX39B	4.67E-01	9.30E-01	2.63E-04	4.40E-02	3.41E-02	5.45E-01
PSORS1C2	NA	NA	NA	NA	NA	NA
IZUMO1	NA	NA	NA	NA	NA	NA
FAM215B	NA	NA	NA	NA	NA	NA
REXO4	4.47E-02	5.82E-01	9.06E-01	9.91E-01	2.78E-01	8.46E-01
ACSF3	6.22E-03	3.70E-01	7.70E-01	9.77E-01	3.88E-01	8.83E-01
RAVER1	1.29E-02	4.48E-01	4.83E-06	2.95E-03	1.61E-05	7.87E-03
ICAM5	3.91E-02	5.63E-01	6.14E-03	2.62E-01	1.25E-01	7.41E-01
OAS1	1.64E-04	6.29E-02	4.43E-05	1.43E-02	2.32E-03	2.16E-01
ICAM3	9.40E-01	9.94E-01	4.90E-01	9.27E-01	3.55E-01	8.74E-01
ZCCHC4	3.17E-04	9.04E-02	1.23E-04	2.53E-02	1.25E-02	4.23E-01
KCNC3	NA	NA	NA	NA	NA	NA
NPNT	1.48E-01	7.56E-01	5.12E-03	2.34E-01	5.71E-03	3.14E-01
WNT3	9.49E-03	4.21E-01	2.89E-10	1.18E-06	1.23E-10	3.01E-07
DCST2	2.36E-02	5.16E-01	5.32E-03	2.36E-01	5.44E-03	3.06E-01
HLA-A	6.92E-01	9.65E-01	2.94E-02	4.85E-01	3.41E-02	5.45E-01
HSD17B14	NA	NA	NA	NA	NA	NA
PI4K2B	2.41E-02	5.19E-01	4.36E-05	1.43E-02	1.29E-02	4.27E-01
CSF3	NA	NA	NA	NA	NA	NA
RPL7A	2.74E-02	5.31E-01	1.04E-01	6.92E-01	7.83E-02	6.81E-01

SEMA6B	1.85E-01	7.96E-01	2.72E-01	8.40E-01	2.10E-01	8.14E-01
SFTPA2	4.57E-01	9.27E-01	2.65E-02	4.70E-01	1.51E-01	7.74E-01
CDH15	NA	NA	NA	NA	NA	NA
RP11-589B3.6	NA	NA	NA	NA	NA	NA
LINC01301	NA	NA	NA	NA	NA	NA
THBS3	4.72E-08	5.77E-05	8.75E-01	9.88E-01	6.63E-04	1.07E-01
RAB2A	3.48E-03	2.99E-01	4.46E-04	6.34E-02	8.38E-03	3.68E-01
SACM1L	2.63E-02	5.29E-01	4.80E-01	9.25E-01	3.62E-01	8.74E-01
IBA57-AS1	NA	NA	NA	NA	NA	NA
MED24	7.21E-05	3.53E-02	1.59E-04	3.03E-02	6.28E-05	2.07E-02
DPF2	2.53E-01	8.44E-01	4.97E-04	6.82E-02	7.27E-01	9.67E-01
GSDMA	8.71E-02	6.76E-01	6.64E-01	9.59E-01	9.18E-01	9.90E-01
PMVK	5.03E-02	5.97E-01	7.15E-01	9.69E-01	1.98E-01	8.06E-01
PSMD3	7.11E-01	9.66E-01	3.84E-01	8.92E-01	8.38E-01	9.79E-01
RP11-506M13.3	2.85E-02	5.31E-01	1.63E-04	3.06E-02	9.15E-04	1.22E-01
NUTM2E	NA	NA	NA	NA	NA	NA
CSK	3.59E-04	9.66E-02	4.67E-05	1.43E-02	1.11E-01	7.19E-01
RP4-535B20.1	NA	NA	NA	NA	NA	NA
MICB	1.08E-01	7.11E-01	2.00E-03	1.47E-01	2.19E-02	5.01E-01
RTP3	NA	NA	NA	NA	NA	NA
RP11-387H17.6	NA	NA	NA	NA	NA	NA
ARL17A	NA	NA	NA	NA	NA	NA
HLA-DPA1	4.54E-02	5.85E-01	3.37E-01	8.73E-01	4.05E-01	8.89E-01
PSORS1C1	NA	NA	NA	NA	NA	NA
RP3-462D8.2	NA	NA	NA	NA	NA	NA
MEF2D	1.80E-02	4.97E-01	2.87E-01	8.44E-01	7.63E-01	9.73E-01
ICAM1	1.58E-02	4.80E-01	1.49E-05	6.75E-03	2.99E-04	6.30E-02
RP11-11C20.3	NA	NA	NA	NA	NA	NA
RPL24	2.55E-07	2.60E-04	1.23E-04	2.53E-02	1.67E-03	1.85E-01
SLC22A4	3.02E-01	8.73E-01	3.17E-01	8.59E-01	4.08E-01	8.90E-01
GBA	NA	NA	NA	NA	NA	NA
MCM7	2.88E-01	8.66E-01	7.75E-01	9.77E-01	8.41E-02	6.92E-01
COQ10B	NA	NA	NA	NA	NA	NA
LTA	3.42E-01	8.86E-01	2.99E-03	1.84E-01	4.83E-02	6.02E-01
IMPG2	3.37E-01	8.83E-01	9.21E-02	6.70E-01	3.16E-02	5.39E-01
LRRC37A2	NA	NA	NA	NA	NA	NA
DBP	NA	NA	NA	NA	NA	NA
CLK3	2.48E-01	8.41E-01	1.15E-02	3.41E-01	1.71E-03	1.88E-01
ZNF3	3.94E-01	9.06E-01	1.59E-03	1.31E-01	1.52E-02	4.49E-01
ZNF778	4.04E-01	9.08E-01	2.10E-01	8.06E-01	9.71E-01	9.96E-01
PPP1R11	1.76E-01	7.83E-01	1.97E-01	7.97E-01	2.65E-02	5.11E-01
SLC5A3	NA	NA	NA	NA	NA	NA

CDSN	NA	NA	NA	NA	NA	NA
ACSL6	2.44E-05	1.32E-02	8.14E-07	7.11E-04	3.40E-06	2.31E-03
PGC	NA	NA	NA	NA	NA	NA
DSP	NA	NA	NA	NA	NA	NA
HLA-DQA2	4.88E-03	3.46E-01	7.29E-05	1.78E-02	1.26E-07	1.18E-04
IFNAR1	1.35E-01	7.43E-01	3.98E-04	5.90E-02	1.28E-04	3.46E-02
FOXA3	NA	NA	NA	NA	NA	NA
HLA-DPB1	1.72E-02	4.93E-01	7.42E-01	9.75E-01	2.75E-01	8.44E-01
DTX1	7.13E-02	6.46E-01	1.28E-01	7.31E-01	4.28E-01	8.99E-01
MYDGF	2.07E-05	1.21E-02	2.64E-06	1.79E-03	2.66E-07	2.32E-04
RP11-182L21.6	3.21E-01	8.77E-01	1.88E-01	7.91E-01	1.93E-04	4.71E-02
EIF4EBP2	6.45E-02	6.30E-01	4.05E-01	9.01E-01	5.36E-01	9.36E-01
ANAPC4	2.04E-03	2.42E-01	1.25E-06	1.01E-03	1.22E-04	3.39E-02
SLC22A31	1.79E-01	7.88E-01	1.77E-05	7.46E-03	2.92E-04	6.25E-02
SPACA4	NA	NA	NA	NA	NA	NA
TFR2	5.27E-02	6.02E-01	7.25E-01	9.71E-01	6.26E-01	9.53E-01
HLA-DRA	NA	NA	NA	NA	NA	NA
ZKSCAN1	4.25E-01	9.15E-01	3.82E-01	8.90E-01	6.71E-02	6.58E-01
SLC25A44	NA	NA	NA	NA	NA	NA
PMF1	3.20E-06	2.45E-03	1.64E-02	3.94E-01	2.01E-04	4.71E-02
MOSPD3	1.90E-07	2.11E-04	9.21E-04	9.54E-02	2.34E-03	2.16E-01
BYSL	5.81E-01	9.57E-01	5.16E-01	9.36E-01	7.31E-01	9.68E-01
CTA-384D8.35	5.36E-01	9.46E-01	3.12E-01	8.57E-01	1.65E-02	4.61E-01
ACTL6B	NA	NA	NA	NA	NA	NA
HIP1	4.40E-02	5.79E-01	1.76E-05	7.46E-03	1.09E-07	1.11E-04
EFNA1	7.36E-01	9.69E-01	1.14E-05	5.36E-03	2.47E-09	4.31E-06
CDC37	NA	NA	NA	NA	NA	NA
CHD1	1.36E-01	7.43E-01	1.60E-01	7.61E-01	1.86E-01	8.03E-01
PCOLCE	7.17E-01	9.66E-01	5.86E-02	5.99E-01	2.25E-01	8.20E-01
AC006007.1	NA	NA	NA	NA	NA	NA
CA11	2.10E-02	5.12E-01	2.77E-01	8.42E-01	1.96E-01	8.06E-01
FUBP1	2.59E-03	2.63E-01	5.16E-07	4.85E-04	2.03E-06	1.46E-03
SH3BP2	1.46E-01	7.54E-01	1.33E-01	7.34E-01	2.17E-01	8.19E-01
EFNA3	7.01E-01	9.65E-01	7.01E-02	6.29E-01	9.39E-01	9.92E-01
C2orf16	NA	NA	NA	NA	NA	NA
MYL5	3.11E-01	8.75E-01	3.13E-02	4.97E-01	2.11E-01	8.14E-01
ADGRE3	1.01E-02	4.24E-01	3.82E-01	8.90E-01	5.60E-01	9.42E-01
CLMP	2.19E-01	8.17E-01	3.44E-01	8.75E-01	9.40E-02	7.03E-01
HLA-F	2.05E-01	8.07E-01	1.92E-01	7.92E-01	5.11E-02	6.13E-01
GATS	6.77E-01	9.61E-01	8.38E-01	9.85E-01	3.45E-02	5.47E-01
FDPS	6.44E-01	9.61E-01	4.87E-01	9.27E-01	1.21E-02	4.21E-01
ZAN	NA	NA	NA	NA	NA	NA
TNFRSF4	2.43E-01	8.38E-01	2.59E-01	8.34E-01	1.75E-01	7.91E-01
BCAT2	2.27E-03	2.46E-01	7.29E-01	9.71E-01	6.74E-01	9.62E-01

CYP1A1	7.15E-01	9.66E-01	1.32E-01	7.34E-01	7.39E-01	9.70E-01
TOMM7	2.37E-01	8.31E-01	9.73E-02	6.76E-01	1.00E-02	4.00E-01
YIPF2	4.93E-01	9.37E-01	6.75E-01	9.62E-01	2.83E-02	5.20E-01
POU5F1	6.84E-01	9.64E-01	5.18E-01	9.36E-01	5.24E-01	9.32E-01
P4HA2	8.23E-01	9.81E-01	4.89E-01	9.27E-01	5.18E-01	9.31E-01
RP11-298J23.8	NA	NA	NA	NA	NA	NA
DNAJB4	NA	NA	NA	NA	NA	NA
NLRP4	NA	NA	NA	NA	NA	NA
FRS3	8.52E-02	6.74E-01	1.50E-03	1.26E-01	7.52E-02	6.79E-01
GPC2	1.35E-03	2.09E-01	4.72E-05	1.43E-02	2.22E-02	5.02E-01
AGRN	2.61E-02	5.29E-01	6.21E-01	9.49E-01	6.18E-01	9.52E-01
COPS6	6.71E-01	9.61E-01	7.72E-01	9.77E-01	4.70E-02	5.99E-01
ATF6B	5.29E-04	1.29E-01	8.98E-05	2.03E-02	5.60E-04	9.63E-02
GUK1	6.04E-01	9.57E-01	4.19E-01	9.09E-01	8.51E-01	9.81E-01
RP11-298J23.10	4.24E-01	9.14E-01	1.48E-01	7.47E-01	1.89E-01	8.03E-01
CLK2	NA	NA	NA	NA	NA	NA
RP13-554M15.8	NA	NA	NA	NA	NA	NA
AZGP1	NA	NA	NA	NA	NA	NA
HCN3	7.96E-01	9.79E-01	4.73E-03	2.28E-01	2.30E-05	1.02E-02
SURF2	2.70E-01	8.54E-01	2.20E-01	8.09E-01	2.33E-01	8.21E-01
JAK1	7.38E-01	9.70E-01	7.52E-01	9.75E-01	7.93E-01	9.74E-01
SCAMP3	NA	NA	NA	NA	NA	NA
LINC00304	4.29E-01	9.16E-01	1.08E-04	2.32E-02	1.96E-05	9.21E-03
ZBTB7B	1.00E-01	6.98E-01	9.07E-01	9.91E-01	5.64E-01	9.42E-01
ACAD8	NA	NA	NA	NA	NA	NA
HLA-DMB	8.87E-01	9.89E-01	6.19E-02	6.09E-01	6.60E-04	1.07E-01
TRIM15	9.76E-01	9.98E-01	2.16E-02	4.48E-01	1.34E-02	4.36E-01
RNF17	NA	NA	NA	NA	NA	NA
KISS1	NA	NA	NA	NA	NA	NA
ABCF1	NA	NA	NA	NA	NA	NA
BTNL2	NA	NA	NA	NA	NA	NA
PKLR	NA	NA	NA	NA	NA	NA
FAM83E	NA	NA	NA	NA	NA	NA
TIMM29	6.18E-01	9.59E-01	8.52E-01	9.87E-01	7.84E-01	9.74E-01
LINC02138	NA	NA	NA	NA	NA	NA
LTB	2.34E-02	5.16E-01	1.07E-03	1.01E-01	5.21E-02	6.18E-01
DDR1	9.33E-01	9.94E-01	3.94E-01	8.94E-01	7.57E-01	9.73E-01
TDGF1	NA	NA	NA	NA	NA	NA
RSPO2	NA	NA	NA	NA	NA	NA
SFTPA1	9.37E-01	9.94E-01	6.86E-01	9.65E-01	2.67E-01	8.41E-01
RIT1	6.38E-01	9.61E-01	6.02E-01	9.48E-01	4.46E-05	1.65E-02
NUTM2B	1.62E-01	7.72E-01	2.26E-02	4.52E-01	2.61E-02	5.11E-01
KIAA0319L	9.89E-03	4.24E-01	2.12E-05	8.36E-03	6.35E-04	1.05E-01
ANHX	NA	NA	NA	NA	NA	NA

C17orf78	NA	NA	NA	NA	NA	NA
C1orf35	4.82E-01	9.34E-01	3.38E-01	8.73E-01	2.14E-01	8.15E-01
RHOF	6.05E-01	9.57E-01	7.30E-02	6.35E-01	5.27E-02	6.19E-01
SYT11	6.74E-01	9.61E-01	1.98E-01	7.97E-01	4.67E-05	1.68E-02
PRR15L	2.66E-01	8.53E-01	4.63E-02	5.69E-01	1.07E-01	7.16E-01
STK19	3.62E-01	8.96E-01	1.40E-01	7.37E-01	2.40E-03	2.16E-01
NECAB3	8.47E-01	9.85E-01	3.36E-01	8.72E-01	2.38E-01	8.21E-01
CNPY4	5.01E-03	3.46E-01	2.62E-04	4.40E-02	7.53E-06	4.00E-03
IFNA6	NA	NA	NA	NA	NA	NA
DST	6.74E-01	9.61E-01	5.80E-01	9.41E-01	9.70E-01	9.96E-01
ABTB2	6.59E-04	1.47E-01	5.10E-07	4.85E-04	3.44E-07	2.80E-04
PRICKLE4	4.12E-01	9.11E-01	1.46E-01	7.44E-01	1.93E-01	8.05E-01
KLC1	9.27E-02	6.84E-01	5.47E-03	2.38E-01	1.96E-02	4.80E-01
SPPL2C	NA	NA	NA	NA	NA	NA
PBXIP1	1.48E-01	7.56E-01	8.13E-01	9.82E-01	4.08E-01	8.90E-01
PPP1R12C	2.49E-05	1.32E-02	2.05E-01	8.03E-01	8.12E-01	9.76E-01
MARS2	NA	NA	NA	NA	NA	NA
UBE2D4	7.68E-01	9.76E-01	3.00E-01	8.49E-01	1.96E-03	2.06E-01
MARCKSL1	NA	NA	NA	NA	NA	NA
APOPT1	2.64E-02	5.29E-01	3.96E-05	1.38E-02	4.90E-06	2.72E-03
DHDDS	5.69E-04	1.31E-01	5.10E-03	2.34E-01	1.58E-02	4.53E-01
NINJ2	3.22E-01	8.77E-01	1.21E-01	7.29E-01	1.26E-03	1.54E-01
WFDC2	2.48E-01	8.41E-01	4.52E-01	9.17E-01	8.35E-01	9.79E-01
VIPR2	NA	NA	NA	NA	NA	NA
GATA6-AS1	NA	NA	NA	NA	NA	NA
BMP1	2.61E-01	8.50E-01	9.57E-02	6.75E-01	3.33E-01	8.71E-01
HLA-B	3.06E-02	5.33E-01	1.91E-04	3.55E-02	1.13E-02	4.12E-01
BGLAP	1.64E-03	2.19E-01	1.33E-01	7.34E-01	2.04E-02	4.89E-01
KANSL1	NA	NA	NA	NA	NA	NA
NELFE	4.07E-01	9.09E-01	7.69E-01	9.77E-01	3.90E-01	8.85E-01
RGS9	2.56E-01	8.46E-01	7.30E-01	9.71E-01	1.99E-01	8.09E-01
SPEF2	NA	NA	NA	NA	NA	NA
CTD-3010D24.3	NA	NA	NA	NA	NA	NA
ZSCAN21	2.23E-03	2.46E-01	1.97E-06	1.42E-03	1.21E-04	3.39E-02
CAMSAP2	6.54E-01	9.61E-01	1.45E-01	7.44E-01	5.39E-02	6.22E-01
UBE2L6	9.25E-01	9.94E-01	4.17E-01	9.07E-01	6.14E-01	9.52E-01
GIGYF1	5.70E-04	1.31E-01	5.15E-01	9.36E-01	4.35E-02	5.93E-01
GOLGA8K	NA	NA	NA	NA	NA	NA
ZCCHC24	3.10E-01	8.75E-01	4.02E-01	8.99E-01	9.45E-01	9.92E-01
TFAP2E	9.01E-02	6.79E-01	4.46E-01	9.17E-01	8.67E-01	9.82E-01
CYP3A43	NA	NA	NA	NA	NA	NA
TCF7L2	1.85E-01	7.96E-01	7.89E-01	9.79E-01	4.31E-01	9.00E-01
SUPT6H	2.31E-02	5.15E-01	1.86E-01	7.87E-01	8.35E-02	6.92E-01
SYMPK	3.87E-03	3.13E-01	1.31E-02	3.58E-01	5.97E-03	3.19E-01

DDAH2	1.32E-01	7.39E-01	3.31E-02	5.05E-01	2.03E-02	4.89E-01
AATK-AS1	NA	NA	NA	NA	NA	NA
CREBRF	NA	NA	NA	NA	NA	NA
C1orf56	7.06E-02	6.45E-01	5.14E-03	2.34E-01	4.26E-05	1.63E-02
MSTO1	NA	NA	NA	NA	NA	NA
ANXA11	2.36E-01	8.31E-01	2.51E-01	8.27E-01	5.43E-01	9.39E-01
TNFSF8	7.70E-01	9.76E-01	8.46E-01	9.86E-01	3.34E-01	8.72E-01
GAL3ST4	9.07E-01	9.91E-01	7.32E-01	9.72E-01	2.35E-01	8.21E-01
COL11A2	6.12E-01	9.58E-01	1.66E-01	7.68E-01	1.17E-01	7.26E-01
SPOP	5.84E-01	9.57E-01	4.33E-01	9.13E-01	5.05E-01	9.27E-01
PLSCR2	1.66E-01	7.77E-01	7.75E-04	8.61E-02	1.88E-04	4.71E-02
KLF1	2.28E-01	8.24E-01	3.13E-02	4.97E-01	1.67E-01	7.83E-01
ARL17B	9.11E-01	9.91E-01	NA	NA	NA	NA
C6orf48	3.57E-01	8.92E-01	2.76E-01	8.42E-01	7.73E-01	9.73E-01
RPP21	1.39E-01	7.47E-01	4.74E-02	5.74E-01	2.63E-02	5.11E-01
RP11-57G10.8	1.34E-01	7.40E-01	7.86E-01	9.78E-01	8.32E-01	9.78E-01
YLPM1	NA	NA	NA	NA	NA	NA
GATAD2A	3.71E-01	9.00E-01	7.38E-01	9.73E-01	5.23E-01	9.31E-01
OAS2	NA	NA	NA	NA	NA	NA
HEY1	1.72E-02	4.93E-01	5.61E-01	9.39E-01	2.41E-01	8.23E-01
YY1AP1	1.34E-02	4.52E-01	5.27E-01	9.37E-01	1.34E-01	7.55E-01
FCHSD2	1.91E-01	8.02E-01	9.87E-01	9.99E-01	5.11E-01	9.30E-01
MAPT	NA	NA	NA	NA	NA	NA
GABARAPL2	5.69E-01	9.55E-01	2.75E-01	8.42E-01	9.19E-01	9.90E-01
CYP4B1	NA	NA	NA	NA	NA	NA
RP11-91I20.3	NA	NA	NA	NA	NA	NA
THRA	9.73E-01	9.98E-01	8.77E-01	9.88E-01	6.19E-01	9.52E-01
EDC3	NA	NA	NA	NA	NA	NA
SPARC	NA	NA	NA	NA	NA	NA
NPRL3	1.74E-01	7.80E-01	6.88E-01	9.65E-01	2.33E-01	8.21E-01
EPS8L1	1.46E-03	2.17E-01	7.04E-02	6.29E-01	6.71E-01	9.61E-01
CFB	NA	NA	NA	NA	NA	NA
AC114752.1	NA	NA	NA	NA	NA	NA
CCDC18	2.11E-01	8.12E-01	8.02E-01	9.81E-01	1.41E-01	7.63E-01
LINC01315	NA	NA	NA	NA	NA	NA
NCOR1	1.10E-03	1.92E-01	1.03E-05	5.25E-03	9.81E-05	2.85E-02
SNX1	6.54E-01	9.61E-01	8.65E-02	6.60E-01	9.28E-02	7.03E-01
DNAH8	NA	NA	NA	NA	NA	NA
RAVER2	4.49E-01	9.24E-01	3.07E-01	8.55E-01	5.07E-01	9.29E-01
HIST1H2AB	NA	NA	NA	NA	NA	NA
SQRDL	1.47E-01	7.56E-01	5.49E-04	7.18E-02	1.66E-01	7.83E-01
FNIP1	4.56E-02	5.85E-01	2.84E-02	4.78E-01	3.80E-01	8.79E-01
TCF12	1.43E-04	5.81E-02	1.84E-01	7.85E-01	2.51E-01	8.26E-01

EPO	NA	NA	NA	NA	NA	NA
CTC-296K1.3	NA	NA	NA	NA	NA	NA
LAMTOR4	7.71E-01	9.76E-01	5.65E-01	9.39E-01	3.11E-02	5.37E-01
CTB-50L17.16	NA	NA	NA	NA	NA	NA
DONSON	2.16E-01	8.13E-01	2.26E-04	4.00E-02	8.00E-03	3.65E-01
ZPBP2	NA	NA	NA	NA	NA	NA
NFAM1	NA	NA	NA	NA	NA	NA
NCDN	4.61E-02	5.87E-01	9.18E-01	9.91E-01	9.74E-01	9.97E-01
ORMDL3	1.65E-04	6.29E-02	4.90E-05	1.43E-02	1.04E-02	4.05E-01
ZNF655	6.39E-01	9.61E-01	3.99E-01	8.97E-01	8.22E-01	9.78E-01
IBA57	6.78E-02	6.39E-01	3.36E-05	1.27E-02	8.09E-02	6.87E-01
CALR	4.46E-01	9.22E-01	9.16E-01	9.91E-01	8.44E-01	9.79E-01
CPOX	8.36E-01	9.84E-01	2.32E-01	8.17E-01	5.69E-01	9.44E-01
FAT2	5.02E-02	5.97E-01	9.14E-01	9.91E-01	7.61E-01	9.73E-01
ST3GAL3	9.39E-01	9.94E-01	1.02E-02	3.28E-01	1.02E-02	4.02E-01
DNAJB14	6.24E-01	9.59E-01	2.78E-01	8.42E-01	7.42E-01	9.70E-01
LINC01565	NA	NA	NA	NA	NA	NA
SNRPD2	4.05E-03	3.22E-01	1.09E-05	5.33E-03	2.33E-05	1.02E-02
GNB2	4.22E-02	5.78E-01	1.05E-01	6.93E-01	8.94E-02	6.97E-01
TSACC	2.30E-01	8.26E-01	4.80E-02	5.79E-01	1.79E-01	7.97E-01
DCST1	2.36E-02	5.16E-01	5.32E-03	2.36E-01	5.44E-03	3.06E-01
6-Mar	2.60E-01	8.50E-01	8.01E-01	9.81E-01	3.67E-01	8.74E-01
TRAF3	4.29E-03	3.22E-01	1.07E-01	6.96E-01	4.10E-02	5.80E-01
ZMYM4	2.15E-04	7.31E-02	1.27E-03	1.13E-01	4.02E-03	2.72E-01
AQP4	NA	NA	NA	NA	NA	NA
ARID3B	9.22E-02	6.84E-01	2.29E-03	1.54E-01	3.75E-06	2.41E-03
AP1M2	4.62E-01	9.30E-01	5.08E-01	9.34E-01	4.23E-01	8.97E-01
FAM189B	3.70E-01	8.99E-01	1.32E-01	7.34E-01	6.49E-03	3.32E-01
ADAMTS8	NA	NA	NA	NA	NA	NA
MICA	9.35E-02	6.84E-01	6.89E-05	1.75E-02	9.76E-05	2.85E-02
PRSS33	NA	NA	NA	NA	NA	NA
DAP3	8.78E-01	9.87E-01	1.30E-01	7.34E-01	2.69E-03	2.30E-01
LINC01149	NA	NA	NA	NA	NA	NA
TAF6	5.81E-02	6.12E-01	6.87E-04	8.07E-02	5.37E-02	6.22E-01
GPANK1	2.91E-02	5.31E-01	8.68E-02	6.61E-01	6.72E-03	3.39E-01
LINC00310	6.18E-01	9.59E-01	3.93E-01	8.94E-01	6.08E-01	9.52E-01
RP11-503G7.2	NA	NA	NA	NA	NA	NA
FRYL	NA	NA	NA	NA	NA	NA
E2F1	NA	NA	NA	NA	NA	NA
PRRT1	2.66E-01	8.53E-01	2.61E-02	4.68E-01	2.68E-02	5.11E-01
IRF7	1.68E-01	7.77E-01	3.86E-01	8.93E-01	5.00E-01	9.26E-01
LINC00857	NA	NA	NA	NA	NA	NA
CERCAM	NA	NA	NA	NA	NA	NA
ZSWIM7	NA	NA	NA	NA	NA	NA

ADRA1A	NA	NA	NA	NA	NA	NA
NPY4R	NA	NA	NA	NA	NA	NA
TRMT61B	3.04E-03	2.80E-01	7.86E-05	1.85E-02	9.96E-02	7.07E-01
GJC2	NA	NA	NA	NA	NA	NA
PSMB2	3.26E-01	8.79E-01	4.12E-02	5.38E-01	2.60E-01	8.34E-01
PTGFR	6.98E-01	9.65E-01	2.56E-01	8.31E-01	4.30E-01	8.99E-01
VPS29	5.58E-01	9.52E-01	9.24E-02	6.71E-01	1.07E-01	7.16E-01
LINC00348	NA	NA	NA	NA	NA	NA
BRD2	3.05E-03	2.80E-01	1.04E-02	3.29E-01	8.73E-01	9.83E-01
UPF2	NA	NA	NA	NA	NA	NA
CHCHD7	1.30E-01	7.35E-01	1.61E-02	3.91E-01	1.47E-02	4.44E-01
HIST1H2BB	NA	NA	NA	NA	NA	NA
HSPE1	9.47E-01	9.96E-01	1.31E-01	7.34E-01	3.52E-03	2.57E-01
RP11-499E18.1	NA	NA	NA	NA	NA	NA
GCDH	1.41E-01	7.50E-01	8.09E-01	9.82E-01	9.38E-01	9.92E-01
ZNF146	4.20E-01	9.14E-01	2.51E-01	8.27E-01	6.14E-01	9.52E-01
CYP2C19	NA	NA	NA	NA	NA	NA
BNIP1	4.88E-01	9.37E-01	7.15E-02	6.33E-01	6.54E-01	9.57E-01
DR1	7.12E-02	6.46E-01	2.25E-01	8.11E-01	9.76E-01	9.97E-01
SUCLG2	8.37E-01	9.84E-01	9.91E-01	9.99E-01	3.57E-01	8.74E-01
TADA1	8.16E-01	9.81E-01	6.93E-01	9.67E-01	5.08E-01	9.29E-01
PRM3	NA	NA	NA	NA	NA	NA
PREX1	9.92E-01	9.99E-01	7.54E-01	9.75E-01	7.30E-01	9.67E-01
FAM96A	NA	NA	NA	NA	NA	NA
WFDC10A	NA	NA	NA	NA	NA	NA
AIP	NA	NA	NA	NA	NA	NA
PNRC1	2.89E-01	8.66E-01	5.26E-02	5.93E-01	2.52E-03	2.20E-01
XRCC3	1.92E-02	4.98E-01	4.38E-04	6.30E-02	7.98E-04	1.14E-01
LPCAT2	5.90E-01	9.57E-01	5.74E-01	9.40E-01	4.30E-01	8.99E-01
ATG16L2	NA	NA	NA	NA	NA	NA
ACSM4	NA	NA	NA	NA	NA	NA
ZNF311	NA	NA	NA	NA	NA	NA
HNMT	4.25E-02	5.78E-01	2.20E-02	4.51E-01	1.57E-01	7.81E-01
PDLIM4	7.95E-01	9.79E-01	9.35E-01	9.92E-01	7.51E-01	9.72E-01
ILF3-AS1	7.35E-03	3.89E-01	9.33E-01	9.92E-01	9.53E-01	9.94E-01
PPP1R2	1.97E-01	8.05E-01	5.24E-01	9.36E-01	4.11E-01	8.92E-01
REEP4	1.94E-01	8.03E-01	5.65E-01	9.39E-01	7.88E-01	9.74E-01
RPN1	9.30E-01	9.94E-01	6.12E-01	9.48E-01	1.69E-01	7.85E-01
LPIN3	NA	NA	NA	NA	NA	NA
ARHGEF38	NA	NA	NA	NA	NA	NA
AP4M1	1.94E-02	5.02E-01	5.22E-04	6.97E-02	2.19E-02	5.01E-01
ZSCAN23	NA	NA	NA	NA	NA	NA
ZNF84	3.52E-01	8.92E-01	2.27E-02	4.52E-01	2.93E-02	5.27E-01
LMAN1L	2.27E-01	8.24E-01	5.79E-01	9.41E-01	6.76E-01	9.62E-01
PROB1	7.17E-04	1.53E-01	6.90E-02	6.26E-01	1.82E-02	4.72E-01

RP11-16E12.2	3.40E-01	8.86E-01	5.69E-02	5.95E-01	6.58E-02	6.56E-01
BMP8A	4.17E-02	5.78E-01	2.51E-01	8.27E-01	1.40E-02	4.42E-01
GSDMB	1.38E-03	2.11E-01	3.43E-05	1.27E-02	6.14E-03	3.25E-01
UNC5C	NA	NA	NA	NA	NA	NA
RASSF3	2.73E-01	8.56E-01	7.73E-04	8.61E-02	3.66E-01	8.74E-01
IMPG1	NA	NA	NA	NA	NA	NA
NDUFC1	6.95E-01	9.65E-01	7.10E-01	9.68E-01	8.85E-01	9.84E-01
DKFZP434 L187	NA	NA	NA	NA	NA	NA
FAM126A	7.44E-01	9.71E-01	1.91E-01	7.92E-01	3.41E-01	8.73E-01
CPLX3	7.64E-02	6.54E-01	1.00E-01	6.83E-01	9.50E-01	9.93E-01
IKZF3	6.26E-04	1.42E-01	1.24E-04	2.53E-02	1.99E-03	2.06E-01
KIAA0907	3.10E-01	8.75E-01	7.86E-01	9.78E-01	2.03E-03	2.07E-01
ZKSCAN4	3.18E-01	8.76E-01	5.80E-02	5.99E-01	3.18E-01	8.65E-01
LKAAEAR1	1.29E-01	7.35E-01	4.84E-04	6.72E-02	1.83E-01	8.01E-01
DYNC1H1	NA	NA	NA	NA	NA	NA
GNGT2	2.93E-02	5.31E-01	9.79E-02	6.76E-01	7.03E-01	9.65E-01
CDC42EP3	3.86E-01	9.05E-01	4.54E-01	9.18E-01	1.90E-01	8.03E-01
NAA20	2.71E-01	8.55E-01	3.27E-01	8.68E-01	3.10E-01	8.62E-01
MS4A4A	3.44E-01	8.86E-01	4.55E-01	9.18E-01	1.71E-01	7.87E-01
OBSCN	NA	NA	NA	NA	NA	NA
C1orf145	3.32E-02	5.46E-01	7.48E-05	1.79E-02	1.83E-02	4.73E-01
CDH16	NA	NA	NA	NA	NA	NA
USP49	4.36E-02	5.78E-01	6.65E-03	2.70E-01	7.65E-01	9.73E-01
SNX19	1.70E-01	7.77E-01	1.96E-02	4.24E-01	2.09E-01	8.14E-01
FBXL18	3.42E-01	8.86E-01	5.04E-01	9.32E-01	9.97E-01	9.99E-01
VWA7	1.87E-01	7.99E-01	7.73E-02	6.46E-01	6.65E-02	6.57E-01
NAA15	7.16E-01	9.66E-01	2.46E-03	1.62E-01	6.69E-02	6.57E-01
DHX30	NA	NA	NA	NA	NA	NA
PCMTD1	8.23E-01	9.81E-01	1.01E-01	6.85E-01	8.62E-02	6.94E-01
HLA-DOA	1.62E-01	7.71E-01	3.66E-03	2.05E-01	7.66E-02	6.79E-01
RFTN2	5.73E-01	9.56E-01	7.51E-01	9.75E-01	6.76E-02	6.60E-01
ALKBH1	NA	NA	NA	NA	NA	NA
LDLR	3.46E-01	8.88E-01	6.90E-01	9.66E-01	8.61E-01	9.82E-01
DSCAML1	NA	NA	NA	NA	NA	NA
LCTL	NA	NA	NA	NA	NA	NA
HINT1	9.51E-01	9.96E-01	2.80E-01	8.43E-01	3.00E-02	5.30E-01
MAP2	8.91E-03	4.15E-01	7.15E-02	6.33E-01	7.06E-01	9.65E-01
STAG3	2.81E-03	2.70E-01	9.35E-05	2.08E-02	2.42E-03	2.16E-01
IRF4	3.56E-02	5.55E-01	3.15E-04	5.00E-02	5.89E-02	6.37E-01
PKP2	1.83E-01	7.93E-01	6.11E-01	9.48E-01	5.52E-01	9.39E-01
EPB41L2	8.49E-01	9.85E-01	3.38E-03	1.96E-01	3.18E-01	8.65E-01
MSH5	7.33E-01	9.69E-01	4.22E-02	5.43E-01	8.36E-01	9.79E-01

Note: NA represents not applicable.

73 **Supplementary Table S10. The S-MultiXcan-identified 438 risk genes validated by**
74 **MAGMA-based gene-level association analysis**

Gene Name	Susceptible COVID-19 (P-value)	Susceptible COVID-19 (FDR)	Hospitalized COVID-19 (P-value)	Hospitalized COVID-19 (FDR)	Very severe COVID-19 (P-value)	Very severe COVID-19 (FDR)
CCR9	2.18E-35	7.18E-32	8.56E-57	5.63E-53	3.60E-60	1.78E-56
SLC6A20	1.10E-96	2.17E-92	1.03E-74	1.02E-70	1.09E-75	1.08E-71
CXCR6	1.84E-30	3.64E-27	4.00E-44	1.13E-40	5.71E-46	1.41E-42
XCR1	3.14E-26	4.78E-23	3.68E-54	1.45E-50	1.60E-55	6.33E-52
LZTFL1	8.49E-64	8.39E-60	2.68E-75	5.29E-71	7.98E-78	1.58E-73
ABO	4.14E-47	2.73E-43	2.33E-13	1.92E-10	3.91E-09	1.00E-06
CCR3	1.66E-18	1.93E-15	2.19E-43	5.40E-40	2.12E-49	5.99E-46
FYCO1	7.75E-37	3.06E-33	2.90E-56	1.43E-52	1.38E-61	9.09E-58
DPP9	5.92E-16	6.50E-13	2.33E-23	3.54E-20	2.16E-25	3.28E-22
IFNAR2	5.74E-19	7.09E-16	1.28E-51	4.21E-48	6.94E-51	2.29E-47
CCR1	2.91E-13	1.69E-10	5.03E-39	1.10E-35	7.10E-40	1.56E-36
CCR5	4.41E-11	1.82E-08	3.41E-27	5.61E-24	1.55E-30	2.79E-27
CCR2	5.88E-13	3.19E-10	7.70E-32	1.52E-28	6.48E-36	1.28E-32
IL10RB	4.85E-10	1.59E-07	1.01E-27	1.81E-24	2.13E-28	3.51E-25
CCRL2	1.19E-05	1.67E-03	4.53E-16	5.59E-13	8.40E-18	1.11E-14
NFKBIZ	2.03E-22	2.87E-19	1.86E-07	3.06E-05	5.98E-03	1.77E-01
NXPE3	7.59E-40	3.75E-36	7.51E-14	6.74E-11	2.17E-06	3.02E-04
MTX1	7.16E-12	3.45E-09	7.38E-10	1.80E-07	1.70E-08	3.82E-06
CEP97	5.77E-33	1.63E-29	1.19E-12	7.12E-10	2.66E-05	2.68E-03
FOXP4	1.02E-03	5.84E-02	2.71E-06	3.30E-04	4.95E-05	4.44E-03
MUC1	7.93E-14	5.60E-11	6.84E-14	6.43E-11	5.17E-14	6.01E-11
ZBTB11	6.69E-30	1.20E-26	1.97E-12	1.11E-09	1.41E-05	1.53E-03
ASH1L	2.50E-04	2.10E-02	4.04E-07	6.13E-05	1.41E-08	3.20E-06
AP000569.9	NA	NA	NA	NA	NA	NA
CACFD1	1.90E-19	2.50E-16	4.23E-03	1.06E-01	1.91E-02	3.49E-01
TRIM46	1.43E-13	9.75E-11	4.30E-12	2.02E-09	3.30E-12	2.17E-09
ATP5O	7.96E-07	1.64E-04	3.65E-14	3.60E-11	3.14E-12	2.17E-09
NAPSA	3.18E-09	9.53E-07	2.99E-14	3.11E-11	2.69E-10	8.78E-08
TULP2	3.05E-14	2.41E-11	1.34E-04	8.16E-03	1.92E-04	1.35E-02
PCNP	2.40E-14	1.98E-11	1.11E-06	1.55E-04	2.41E-02	3.92E-01
MRPS6	7.48E-02	5.62E-01	7.90E-04	3.26E-02	5.88E-02	5.71E-01
SENP7	1.41E-10	5.36E-08	1.86E-03	5.98E-02	3.96E-01	8.85E-01
LINC02009	NA	NA	NA	NA	NA	NA
TYK2	3.12E-03	1.24E-01	7.69E-10	1.85E-07	1.53E-10	5.60E-08
FUT2	2.73E-13	1.64E-10	1.35E-08	2.67E-06	1.02E-09	2.84E-07
MED22	4.91E-10	1.59E-07	5.35E-02	4.52E-01	8.22E-02	6.35E-01
SLC50A1	7.57E-12	3.52E-09	1.44E-06	1.95E-04	2.24E-07	4.10E-05
ELF5	2.16E-08	5.93E-06	5.95E-17	7.83E-14	3.86E-15	4.77E-12
CAT	1.15E-06	2.25E-04	3.24E-12	1.64E-09	9.74E-11	3.85E-08
LTF	1.75E-06	3.30E-04	1.69E-22	2.38E-19	1.90E-22	2.68E-19
C6orf15	1.47E-04	1.41E-02	1.16E-07	1.99E-05	1.72E-08	3.82E-06

SLC2A6	2.24E-14	1.98E-11	3.17E-02	3.54E-01	9.70E-02	6.72E-01
ADAM15	8.30E-08	2.03E-05	1.26E-12	7.32E-10	3.20E-12	2.17E-09
PLEKHA4	6.52E-16	6.78E-13	1.09E-05	1.09E-03	1.25E-04	9.41E-03
TNFAIP8L1	7.76E-08	1.94E-05	5.52E-09	1.15E-06	9.58E-10	2.74E-07
NTN5	5.52E-06	8.40E-04	7.42E-03	1.54E-01	2.74E-03	1.03E-01
ATP11A	1.44E-01	6.73E-01	9.50E-07	1.37E-04	3.51E-07	6.09E-05
ADAMTS13	4.44E-15	4.18E-12	4.68E-02	4.28E-01	3.47E-02	4.60E-01
EFNA4	1.98E-08	5.51E-06	6.07E-13	4.13E-10	5.70E-13	4.90E-10
RP11-798G7.6	NA	NA	NA	NA	NA	NA
PPP1R15A	6.74E-14	4.94E-11	6.68E-05	4.74E-03	9.11E-05	7.26E-03
GON4L	6.47E-06	9.62E-04	9.23E-03	1.77E-01	2.56E-05	2.62E-03
FBRSL1	7.87E-08	1.94E-05	4.32E-13	3.16E-10	8.88E-12	5.16E-09
RASIP1	1.15E-15	1.14E-12	3.88E-09	8.61E-07	2.10E-11	1.05E-08
CCHCR1	4.48E-08	1.18E-05	1.52E-11	6.25E-09	2.28E-11	1.05E-08
NUCB1	2.40E-11	1.03E-08	2.93E-03	8.14E-02	8.39E-03	2.17E-01
MAMSTR	2.39E-14	1.98E-11	5.04E-10	1.29E-07	2.26E-11	1.05E-08
KRTCAP2	5.97E-13	3.19E-10	2.82E-09	6.33E-07	1.06E-09	2.91E-07
OAS3	7.66E-12	3.52E-09	1.93E-14	2.13E-11	4.72E-10	1.48E-07
FDX2	NA	NA	NA	NA	NA	NA
PDE4A	1.56E-02	3.10E-01	9.30E-06	9.41E-04	1.34E-07	2.55E-05
PLEKHM1	6.15E-03	1.87E-01	2.40E-10	6.77E-08	2.71E-10	8.78E-08
FUT1	2.01E-13	1.24E-10	4.38E-06	4.91E-04	8.08E-10	2.46E-07
SFTPD	1.29E-07	3.04E-05	4.75E-10	1.25E-07	9.74E-10	2.75E-07
ZGLP1	5.35E-04	3.75E-02	1.04E-10	3.31E-08	2.07E-12	1.57E-09
TCF19	2.34E-08	6.34E-06	4.04E-11	1.48E-08	7.47E-12	4.47E-09
C5orf56	NA	NA	NA	NA	NA	NA
HLA-C	7.26E-04	4.61E-02	1.44E-05	1.34E-03	2.48E-07	4.47E-05
ARHGAP27	4.70E-04	3.39E-02	9.96E-13	6.14E-10	8.86E-11	3.57E-08
SLC35B1	1.78E-02	3.25E-01	1.69E-07	2.80E-05	2.93E-05	2.91E-03
DDX39B	1.54E-03	7.54E-02	6.30E-11	2.22E-08	2.41E-09	6.44E-07
PSORS1C2	1.12E-08	3.16E-06	6.34E-13	4.17E-10	3.42E-12	2.18E-09
IZUMO1	1.85E-13	1.18E-10	1.45E-06	1.95E-04	2.12E-10	7.17E-08
FAM215B	NA	NA	NA	NA	NA	NA
REXO4	1.77E-07	4.02E-05	3.08E-01	8.12E-01	1.51E-01	7.58E-01
ACSF3	3.74E-02	4.39E-01	3.11E-08	5.74E-06	1.33E-10	4.96E-08
RAVER1	1.42E-04	1.38E-02	2.46E-11	9.34E-09	1.28E-11	7.23E-09
ICAM5	1.08E-04	1.09E-02	1.06E-11	4.55E-09	1.22E-13	1.27E-10
OAS1	1.31E-11	5.89E-09	1.94E-14	2.13E-11	1.72E-10	5.96E-08
ICAM3	5.26E-04	3.70E-02	1.09E-10	3.42E-08	1.71E-10	5.96E-08
ZCCHC4	5.26E-04	3.70E-02	5.40E-10	1.37E-07	3.44E-05	3.33E-03
KCNC3	4.81E-08	1.25E-05	3.13E-07	4.94E-05	1.07E-04	8.35E-03
NPNT	4.57E-01	8.58E-01	9.09E-03	1.77E-01	1.59E-03	7.23E-02
WNT3	9.06E-02	5.97E-01	3.32E-13	2.62E-10	2.42E-13	2.28E-10
DCST2	1.39E-03	7.03E-02	2.13E-08	4.00E-06	1.30E-08	3.02E-06
HLA-A	3.08E-01	8.07E-01	5.87E-06	6.37E-04	4.10E-08	8.53E-06
HSD17B14	1.89E-11	8.30E-09	3.68E-04	1.79E-02	3.75E-03	1.27E-01

PI4K2B	2.73E-04	2.22E-02	1.94E-07	3.17E-05	5.34E-04	3.15E-02
CSF3	2.43E-10	8.74E-08	1.30E-05	1.25E-03	2.35E-06	3.25E-04
RPL7A	5.54E-10	1.74E-07	4.28E-02	4.11E-01	7.70E-02	6.20E-01
SEMA6B	1.41E-01	6.69E-01	3.70E-02	3.84E-01	3.39E-05	3.32E-03
SFTPA2	1.21E-06	2.35E-04	6.76E-10	1.67E-07	8.20E-10	2.46E-07
CDH15	2.52E-02	3.78E-01	2.45E-10	6.81E-08	4.24E-11	1.75E-08
RP11-589B3.6	NA	NA	NA	NA	NA	NA
LINC01301	NA	NA	NA	NA	NA	NA
THBS3	5.04E-13	2.85E-10	4.14E-12	1.99E-09	1.45E-12	1.15E-09
RAB2A	1.34E-05	1.80E-03	2.40E-11	9.29E-09	2.15E-08	4.72E-06
SACM1L	3.15E-02	4.14E-01	4.87E-10	1.26E-07	1.05E-08	2.50E-06
IBA57-AS1	NA	NA	NA	NA	NA	NA
MED24	1.23E-10	4.77E-08	3.31E-05	2.69E-03	4.26E-06	5.50E-04
DPF2	3.30E-01	8.18E-01	4.02E-02	3.98E-01	4.45E-01	8.94E-01
GSDMA	2.31E-10	8.46E-08	1.18E-06	1.63E-04	9.75E-07	1.48E-04
PMVK	2.39E-04	2.03E-02	5.77E-05	4.30E-03	4.96E-05	4.44E-03
PSMD3	2.83E-10	9.65E-08	1.46E-06	1.95E-04	8.15E-07	1.27E-04
RP11-506M13.3	NA	NA	NA	NA	NA	NA
NUTM2E	NA	NA	NA	NA	NA	NA
CSK	9.65E-03	2.40E-01	1.36E-05	1.28E-03	2.30E-02	3.84E-01
RP4-535B20.1	NA	NA	NA	NA	NA	NA
MICB	3.44E-03	1.31E-01	4.34E-09	9.31E-07	1.97E-07	3.64E-05
RTP3	5.37E-03	1.71E-01	1.42E-11	5.96E-09	1.43E-11	7.85E-09
RP11-387H17.6	NA	NA	NA	NA	NA	NA
ARL17A	3.11E-01	8.09E-01	2.73E-04	1.43E-02	9.11E-02	6.55E-01
HLA-DPA1	8.36E-09	2.40E-06	4.70E-06	5.21E-04	8.94E-03	2.24E-01
PSORS1C1	2.67E-07	5.87E-05	3.96E-12	1.95E-09	8.03E-13	6.61E-10
RP3-462D8.2	NA	NA	NA	NA	NA	NA
MEF2D	6.70E-04	4.36E-02	4.39E-01	8.57E-01	8.53E-01	9.12E-01
ICAM1	7.39E-04	4.65E-02	4.17E-10	1.13E-07	5.46E-13	4.90E-10
RP11-11C20.3	NA	NA	NA	NA	NA	NA
RPL24	7.03E-29	1.16E-25	8.99E-12	3.94E-09	9.04E-05	7.23E-03
SLC22A4	1.67E-02	3.16E-01	2.29E-06	2.84E-04	4.82E-04	2.96E-02
GBA	7.16E-04	4.60E-02	5.20E-03	1.21E-01	1.10E-04	8.49E-03
MCM7	7.51E-03	2.10E-01	3.40E-05	2.75E-03	4.54E-05	4.19E-03
COQ10B	4.98E-01	8.63E-01	2.01E-06	2.59E-04	1.53E-04	1.12E-02
LTA	4.65E-03	1.59E-01	1.74E-09	3.95E-07	4.59E-07	7.75E-05
IMPG2	1.16E-09	3.58E-07	3.27E-03	8.88E-02	3.83E-01	8.81E-01
LRRC37A2	2.40E-01	7.71E-01	2.73E-04	1.43E-02	8.56E-02	6.46E-01
DBP	3.98E-06	6.56E-04	2.41E-02	3.09E-01	1.42E-02	2.92E-01
CLK3	2.94E-01	7.99E-01	4.96E-03	1.18E-01	2.31E-05	2.43E-03
ZNF3	1.98E-03	8.92E-02	9.78E-07	1.40E-04	2.96E-06	3.95E-04
ZNF778	1.20E-01	6.42E-01	2.45E-05	2.11E-03	5.11E-05	4.55E-03

PPP1R11	1.89E-01	7.27E-01	4.87E-02	4.33E-01	1.32E-02	2.79E-01
SLC5A3	4.31E-02	4.63E-01	5.07E-04	2.33E-02	2.84E-02	4.21E-01
CDSN	1.32E-05	1.79E-03	3.98E-09	8.63E-07	4.88E-10	1.51E-07
ACSL6	6.74E-05	7.44E-03	1.09E-06	1.55E-04	6.27E-05	5.48E-03
PGC	2.52E-02	3.78E-01	1.52E-03	5.14E-02	1.51E-01	7.58E-01
DSP	5.70E-01	8.73E-01	4.34E-01	8.56E-01	5.44E-03	1.65E-01
HLA-DQA2	7.06E-01	8.84E-01	6.52E-01	8.88E-01	1.66E-01	7.78E-01
IFNAR1	5.15E-02	4.96E-01	1.10E-04	7.05E-03	4.03E-06	5.28E-04
FOXA3	4.18E-01	8.50E-01	6.64E-04	2.87E-02	7.14E-03	1.98E-01
HLA-DPB1	7.57E-09	2.20E-06	3.88E-06	4.45E-04	3.53E-03	1.23E-01
DTX1	4.79E-02	4.83E-01	4.29E-02	4.12E-01	1.62E-02	3.18E-01
MYDGF	9.92E-07	2.00E-04	1.48E-08	2.88E-06	9.04E-09	2.18E-06
RP11-182L21.6	NA	NA	NA	NA	NA	NA
EIF4EBP2	3.15E-01	8.11E-01	3.34E-01	8.21E-01	9.31E-02	6.59E-01
ANAPC4	7.74E-04	4.84E-02	3.93E-09	8.62E-07	1.48E-05	1.60E-03
SLC22A31	3.24E-02	4.17E-01	1.64E-09	3.76E-07	2.89E-08	6.14E-06
SPACA4	2.63E-05	3.27E-03	7.44E-02	5.22E-01	5.47E-02	5.56E-01
TFR2	5.16E-10	1.65E-07	5.57E-04	2.50E-02	7.09E-04	3.93E-02
HLA-DRA	1.51E-01	6.81E-01	6.68E-03	1.44E-01	8.15E-03	2.12E-01
ZKSCAN1	1.83E-04	1.66E-02	3.29E-07	5.15E-05	2.29E-08	4.92E-06
SLC25A44	2.93E-05	3.62E-03	2.93E-03	8.14E-02	4.37E-04	2.72E-02
PMF1	3.57E-05	4.25E-03	5.14E-03	1.20E-01	4.33E-04	2.71E-02
MOSPD3	2.17E-10	8.09E-08	2.54E-04	1.35E-02	7.98E-04	4.24E-02
BYSL	1.14E-02	2.63E-01	7.36E-06	7.73E-04	1.08E-01	6.92E-01
CTA-384D8.35	NA	NA	NA	NA	NA	NA
ACTL6B	2.26E-09	6.87E-07	3.78E-03	9.82E-02	2.58E-03	1.00E-01
HIP1	4.06E-01	8.46E-01	3.92E-02	3.94E-01	1.31E-04	9.83E-03
EFNA1	4.82E-11	1.94E-08	4.84E-08	8.53E-06	1.32E-08	3.03E-06
CDC37	3.26E-02	4.17E-01	1.46E-05	1.35E-03	5.03E-06	6.25E-04
CHD1	6.01E-02	5.24E-01	1.38E-01	6.46E-01	2.42E-01	8.36E-01
PCOLCE	2.49E-10	8.79E-08	2.39E-04	1.29E-02	8.42E-04	4.43E-02
AC006007.1	NA	NA	NA	NA	NA	NA
CA11	1.03E-06	2.06E-04	8.54E-03	1.68E-01	5.09E-03	1.58E-01
FUBP1	2.01E-01	7.39E-01	3.04E-01	8.10E-01	2.27E-01	8.28E-01
SH3BP2	2.47E-02	3.74E-01	1.12E-02	2.02E-01	1.02E-01	6.80E-01
EFNA3	2.84E-11	1.19E-08	1.68E-11	6.75E-09	1.29E-10	4.90E-08
C2orf16	6.97E-01	8.83E-01	3.16E-01	8.16E-01	7.46E-01	8.99E-01
MYL5	1.66E-01	7.01E-01	2.13E-01	7.42E-01	5.87E-01	8.95E-01
ADGRE3	1.07E-01	6.29E-01	1.05E-01	5.90E-01	4.30E-02	5.05E-01
CLMP	2.51E-01	7.79E-01	2.88E-01	8.00E-01	2.56E-02	4.01E-01
HLA-F	1.96E-02	3.40E-01	1.50E-02	2.37E-01	3.05E-04	1.99E-02
GATS	8.34E-04	5.08E-02	1.66E-06	2.18E-04	1.52E-03	7.01E-02
FDPS	1.85E-01	7.22E-01	1.07E-03	3.97E-02	4.58E-06	5.80E-04
ZAN	5.40E-06	8.28E-04	7.04E-03	1.49E-01	1.83E-01	7.96E-01
TNFRSF4	3.83E-01	8.41E-01	2.41E-01	7.67E-01	2.62E-01	8.48E-01

BCAT2	1.46E-04	1.41E-02	4.82E-02	4.33E-01	8.33E-02	6.39E-01
CYP1A1	1.42E-01	6.69E-01	1.64E-03	5.44E-02	3.19E-04	2.05E-02
TOMM7	3.87E-01	8.42E-01	3.54E-06	4.20E-04	1.32E-07	2.53E-05
YIPF2	2.18E-01	7.53E-01	1.43E-04	8.55E-03	5.83E-08	1.18E-05
POU5F1	3.84E-08	1.03E-05	3.08E-11	1.15E-08	1.26E-10	4.88E-08
P4HA2	3.96E-03	1.43E-01	3.86E-06	4.45E-04	1.20E-02	2.65E-01
RP11-298J23.8	NA	NA	NA	NA	NA	NA
DNAJB4	2.08E-01	7.45E-01	2.66E-01	7.88E-01	1.73E-01	7.89E-01
NLRP4	2.82E-01	7.94E-01	6.27E-01	8.84E-01	4.37E-01	8.92E-01
FRS3	9.09E-04	5.35E-02	1.10E-05	1.10E-03	3.62E-02	4.69E-01
GPC2	1.22E-02	2.72E-01	9.09E-05	6.17E-03	3.84E-03	1.29E-01
AGRN	6.68E-02	5.39E-01	5.13E-02	4.42E-01	2.02E-02	3.61E-01
COPS6	2.86E-03	1.16E-01	3.80E-06	4.41E-04	8.52E-06	9.91E-04
ATF6B	2.66E-01	7.88E-01	4.37E-05	3.41E-03	5.17E-04	3.11E-02
GUK1	1.39E-01	6.66E-01	6.49E-05	4.68E-03	3.44E-04	2.19E-02
RP11-298J23.10	NA	NA	NA	NA	NA	NA
CLK2	1.82E-01	7.21E-01	1.23E-02	2.11E-01	8.74E-06	1.01E-03
RP13-554M15.8	NA	NA	NA	NA	NA	NA
AZGP1	1.58E-03	7.65E-02	2.92E-03	8.14E-02	6.82E-07	1.11E-04
HCN3	1.45E-01	6.76E-01	5.62E-03	1.28E-01	9.42E-06	1.08E-03
SURF2	2.81E-10	9.65E-08	2.68E-02	3.30E-01	4.89E-02	5.31E-01
JAK1	5.62E-02	5.09E-01	1.26E-07	2.13E-05	4.91E-09	1.23E-06
SCAMP3	1.06E-01	6.28E-01	7.95E-03	1.61E-01	1.93E-05	2.06E-03
LINC00304	NA	NA	NA	NA	NA	NA
ZBTB7B	1.79E-03	8.25E-02	4.49E-06	5.01E-04	7.25E-06	8.69E-04
ACAD8	5.92E-01	8.75E-01	2.98E-01	8.06E-01	3.61E-02	4.68E-01
HLA-DMB	2.07E-01	7.44E-01	6.94E-03	1.47E-01	6.82E-05	5.84E-03
TRIM15	4.43E-01	8.57E-01	8.22E-02	5.43E-01	6.26E-03	1.80E-01
RNF17	3.42E-01	8.24E-01	5.46E-01	8.76E-01	4.26E-02	5.03E-01
KISS1	3.07E-01	8.07E-01	3.13E-02	3.53E-01	3.04E-01	8.63E-01
ABCF1	7.28E-01	8.86E-01	8.28E-01	9.07E-01	7.08E-01	8.96E-01
BTNL2	3.50E-01	8.28E-01	2.82E-03	7.98E-02	6.48E-03	1.85E-01
PKLR	3.10E-01	8.09E-01	3.66E-03	9.63E-02	8.05E-06	9.47E-04
FAM83E	1.04E-04	1.06E-02	9.49E-02	5.71E-01	8.75E-02	6.51E-01
TIMM29	NA	NA	NA	NA	NA	NA
LINC02138	NA	NA	NA	NA	NA	NA
LTB	5.81E-03	1.81E-01	1.62E-08	3.10E-06	3.18E-06	4.22E-04
DDR1	3.75E-01	8.36E-01	5.91E-01	8.81E-01	3.91E-01	8.85E-01
TDGF1	3.14E-03	1.24E-01	1.40E-02	2.28E-01	2.12E-03	8.91E-02
RSPO2	4.94E-02	4.90E-01	9.07E-05	6.17E-03	2.91E-02	4.25E-01
SFTPA1	6.37E-05	7.16E-03	5.20E-08	9.08E-06	3.43E-09	8.92E-07
RIT1	1.13E-01	6.37E-01	9.55E-02	5.72E-01	4.78E-05	4.35E-03
NUTM2B	1.80E-02	3.25E-01	1.11E-05	1.10E-03	6.96E-07	1.13E-04
KIAA0319L	4.85E-02	4.86E-01	6.65E-05	4.74E-03	2.42E-03	9.62E-02

ANHX	5.06E-02	4.94E-01	2.11E-03	6.54E-02	8.25E-02	6.37E-01
C17orf78	5.75E-01	8.73E-01	2.34E-02	3.05E-01	1.57E-01	7.67E-01
C1orf35	2.97E-01	8.01E-01	9.98E-06	1.01E-03	7.59E-07	1.20E-04
RHOF	4.81E-01	8.60E-01	8.09E-02	5.41E-01	1.74E-01	7.91E-01
SYT11	1.14E-05	1.61E-03	4.74E-03	1.15E-01	7.75E-06	9.17E-04
PRR15L	2.11E-02	3.49E-01	6.16E-03	1.38E-01	3.36E-01	8.70E-01
STK19	4.36E-02	4.64E-01	1.92E-04	1.07E-02	4.44E-03	1.42E-01
NECAB3	3.40E-02	4.24E-01	5.13E-05	3.88E-03	3.21E-03	1.16E-01
CNPY4	1.85E-02	3.30E-01	4.03E-05	3.21E-03	2.53E-05	2.60E-03
IFNA6	3.23E-04	2.53E-02	9.59E-05	6.31E-03	1.18E-04	9.02E-03
DST	7.96E-01	8.99E-01	9.23E-02	5.67E-01	1.49E-01	7.54E-01
ABTB2	4.34E-02	4.63E-01	1.05E-04	6.80E-03	4.17E-06	5.42E-04
PRICKLE4	1.53E-03	7.52E-02	1.19E-05	1.17E-03	2.75E-02	4.17E-01
KLC1	2.42E-02	3.71E-01	2.70E-04	1.42E-02	6.94E-03	1.94E-01
SPPL2C	4.70E-04	3.39E-02	3.00E-12	1.56E-09	1.66E-11	8.87E-09
PBXIP1	1.05E-06	2.08E-04	8.91E-07	1.29E-04	1.37E-06	2.04E-04
PPP1R12C	1.23E-05	1.70E-03	1.64E-01	6.80E-01	3.80E-01	8.81E-01
MARS2	3.56E-01	8.30E-01	1.56E-04	9.14E-03	1.19E-04	9.04E-03
UBE2D4	7.48E-01	8.90E-01	7.65E-01	8.98E-01	3.13E-01	8.66E-01
MARCKSL1	6.31E-01	8.79E-01	1.64E-01	6.80E-01	9.31E-01	9.45E-01
APOPT1	2.98E-02	4.04E-01	3.76E-05	3.01E-03	7.52E-05	6.27E-03
DHDDS	9.67E-04	5.58E-02	3.79E-03	9.83E-02	4.51E-04	2.80E-02
NINJ2	1.07E-01	6.29E-01	1.33E-01	6.40E-01	2.37E-02	3.89E-01
WFDC2	2.52E-02	3.78E-01	2.58E-01	7.82E-01	8.08E-01	9.03E-01
VIPR2	4.44E-01	8.57E-01	8.45E-01	9.11E-01	8.39E-01	9.09E-01
GATA6-AS1	NA	NA	NA	NA	NA	NA
BMP1	1.04E-02	2.50E-01	6.11E-07	8.93E-05	2.90E-04	1.92E-02
HLA-B	6.22E-03	1.88E-01	1.11E-06	1.55E-04	6.36E-06	7.71E-04
BGLAP	6.42E-05	7.17E-03	3.42E-03	9.17E-02	6.21E-04	3.59E-02
KANSL1	4.53E-03	1.56E-01	1.59E-10	4.68E-08	3.96E-09	1.00E-06
NELFE	9.98E-02	6.16E-01	8.56E-04	3.44E-02	8.19E-03	2.13E-01
RGS9	1.19E-01	6.42E-01	5.35E-01	8.75E-01	7.84E-01	9.00E-01
SPEF2	3.46E-02	4.25E-01	1.95E-03	6.19E-02	1.60E-01	7.73E-01
CTD-3010D24.3	NA	NA	NA	NA	NA	NA
ZSCAN21	5.01E-04	3.56E-02	3.63E-08	6.64E-06	4.03E-07	6.87E-05
CAMSAP2	1.80E-01	7.17E-01	4.91E-02	4.34E-01	2.95E-03	1.08E-01
UBE2L6	3.03E-02	4.06E-01	1.75E-01	6.97E-01	5.67E-01	8.95E-01
GIGYF1	2.10E-06	3.77E-04	3.09E-02	3.52E-01	6.06E-02	5.76E-01
GOLGA8K	NA	NA	NA	NA	NA	NA
ZCCHC24	7.11E-02	5.51E-01	3.97E-02	3.95E-01	2.68E-03	1.02E-01
TFAP2E	3.91E-02	4.46E-01	4.84E-03	1.16E-01	7.54E-02	6.18E-01
CYP3A43	4.77E-01	8.59E-01	3.27E-01	8.19E-01	1.11E-01	6.97E-01
TCF7L2	3.13E-01	8.10E-01	2.84E-02	3.40E-01	5.28E-01	8.95E-01
SUPT6H	3.78E-01	8.38E-01	5.46E-01	8.76E-01	7.70E-01	8.99E-01
SYMPK	4.97E-01	8.63E-01	1.32E-03	4.66E-02	1.27E-02	2.73E-01
DDAH2	3.64E-01	8.33E-01	5.91E-02	4.73E-01	3.47E-03	1.22E-01

AATK-AS1	NA	NA	NA	NA	NA	NA
CREBRF	1.94E-01	7.31E-01	2.83E-03	7.99E-02	6.47E-01	8.95E-01
C1orf56	2.22E-01	7.56E-01	2.19E-01	7.48E-01	1.41E-01	7.44E-01
MSTO1	2.15E-06	3.83E-04	3.12E-07	4.94E-05	7.31E-07	1.17E-04
ANXA11	2.64E-02	3.84E-01	1.71E-03	5.62E-02	5.36E-02	5.52E-01
TNFSF8	2.02E-03	9.01E-02	1.34E-02	2.22E-01	2.16E-02	3.75E-01
GAL3ST4	1.08E-03	6.04E-02	2.09E-06	2.64E-04	8.41E-05	6.79E-03
COL11A2	1.20E-03	6.49E-02	1.74E-02	2.58E-01	4.37E-02	5.08E-01
SPOP	5.00E-02	4.91E-01	1.57E-04	9.16E-03	1.58E-01	7.70E-01
PLSCR2	2.39E-01	7.70E-01	3.83E-03	9.87E-02	2.62E-05	2.67E-03
KLF1	3.57E-04	2.72E-02	1.54E-01	6.66E-01	5.98E-01	8.95E-01
ARL17B	7.27E-02	5.55E-01	2.18E-08	4.06E-06	2.17E-06	3.02E-04
C6orf48	7.71E-02	5.68E-01	4.05E-03	1.02E-01	1.94E-03	8.31E-02
RPP21	6.28E-01	8.79E-01	4.40E-04	2.08E-02	2.02E-03	8.60E-02
RP11-57G10.8	NA	NA	NA	NA	NA	NA
YLPM1	2.03E-01	7.43E-01	1.48E-02	2.35E-01	4.78E-05	4.35E-03
GATAD2A	3.33E-01	8.19E-01	3.95E-01	8.44E-01	9.65E-01	9.68E-01
OAS2	1.06E-07	2.56E-05	6.72E-11	2.33E-08	3.18E-07	5.58E-05
HEY1	5.47E-02	5.08E-01	6.58E-01	8.88E-01	7.05E-01	8.96E-01
YY1AP1	7.24E-04	4.61E-02	1.22E-05	1.19E-03	2.49E-07	4.47E-05
FCHSD2	6.48E-01	8.79E-01	5.49E-02	4.57E-01	9.72E-03	2.35E-01
MAPT	2.26E-04	1.95E-02	5.83E-12	2.62E-09	2.19E-11	1.05E-08
GABARAPL2	2.46E-02	3.74E-01	6.27E-05	4.58E-03	1.06E-02	2.46E-01
CYP4B1	1.80E-02	3.25E-01	7.27E-06	7.72E-04	6.76E-02	5.99E-01
RP11-91I20.3	NA	NA	NA	NA	NA	NA
THRA	1.81E-06	3.38E-04	4.82E-03	1.16E-01	7.48E-03	2.02E-01
EDC3	2.15E-01	7.51E-01	2.62E-03	7.54E-02	6.86E-05	5.84E-03
SPARC	6.92E-02	5.46E-01	3.35E-01	8.21E-01	1.38E-01	7.41E-01
NPRL3	6.13E-01	8.78E-01	9.46E-02	5.71E-01	1.44E-01	7.48E-01
EPS8L1	6.28E-06	9.41E-04	3.11E-02	3.53E-01	5.62E-01	8.95E-01
CFB	1.06E-01	6.27E-01	5.74E-04	2.55E-02	3.74E-03	1.27E-01
AC114752.1	NA	NA	NA	NA	NA	NA
CCDC18	3.67E-03	1.37E-01	4.70E-03	1.15E-01	2.48E-06	3.38E-04
LINC01315	NA	NA	NA	NA	NA	NA
NCOR1	2.22E-02	3.57E-01	1.63E-05	1.49E-03	4.10E-05	3.88E-03
SNX1	4.22E-01	8.51E-01	4.46E-05	3.44E-03	1.06E-02	2.46E-01
DNAH8	3.71E-01	8.36E-01	4.94E-04	2.29E-02	3.73E-01	8.80E-01
RAVER2	7.09E-01	8.84E-01	2.50E-02	3.17E-01	2.36E-03	9.50E-02
HIST1H2AB	2.00E-04	1.78E-02	1.39E-02	2.27E-01	9.24E-01	9.42E-01
SQRDL	2.13E-01	7.49E-01	1.98E-03	6.28E-02	7.03E-02	6.10E-01
FNIP1	1.76E-03	8.22E-02	1.03E-04	6.66E-03	1.12E-03	5.47E-02
TCF12	1.95E-05	2.49E-03	7.15E-01	8.93E-01	4.99E-01	8.95E-01
EPO	1.64E-04	1.51E-02	5.41E-02	4.55E-01	1.87E-01	7.98E-01
CTC-296K1.3	NA	NA	NA	NA	NA	NA

LAMTOR4	2.34E-03	9.93E-02	8.17E-06	8.41E-04	2.96E-05	2.93E-03
CTB-50L17.16	NA	NA	NA	NA	NA	NA
DONSON	1.89E-01	7.27E-01	7.54E-06	7.88E-04	1.53E-02	3.06E-01
ZPBP2	1.68E-03	7.96E-02	3.09E-04	1.56E-02	5.80E-03	1.73E-01
NFAM1	3.58E-07	7.61E-05	9.12E-03	1.77E-01	1.83E-03	8.01E-02
NCDN	1.86E-02	3.32E-01	5.45E-05	4.08E-03	5.59E-03	1.68E-01
ORMDL3	2.28E-07	5.12E-05	1.22E-07	2.08E-05	3.85E-05	3.69E-03
ZNF655	1.79E-01	7.17E-01	9.98E-02	5.82E-01	4.84E-01	8.95E-01
IBA57	3.22E-02	4.16E-01	3.75E-07	5.78E-05	6.84E-04	3.84E-02
CALR	1.27E-01	6.50E-01	6.42E-02	4.89E-01	3.92E-01	8.85E-01
CPOX	3.07E-02	4.09E-01	2.73E-01	7.92E-01	4.63E-02	5.20E-01
FAT2	1.36E-01	6.61E-01	1.05E-01	5.90E-01	6.81E-01	8.96E-01
ST3GAL3	9.24E-01	9.41E-01	2.16E-02	2.91E-01	8.83E-03	2.23E-01
DNAJB14	7.46E-01	8.90E-01	6.16E-02	4.81E-01	1.17E-01	7.08E-01
LINC01565	NA	NA	NA	NA	NA	NA
SNRPD2	3.48E-02	4.27E-01	7.60E-06	7.90E-04	2.55E-03	9.95E-02
GNB2	2.46E-07	5.46E-05	2.45E-02	3.13E-01	1.83E-02	3.42E-01
TSACC	5.98E-03	1.85E-01	2.08E-03	6.47E-02	3.74E-03	1.27E-01
DCST1	4.27E-06	6.92E-04	4.91E-11	1.76E-08	3.05E-10	9.72E-08
6-Mar	5.08E-01	8.63E-01	4.78E-01	8.67E-01	2.65E-01	8.48E-01
TRAF3	1.85E-06	3.42E-04	4.74E-02	4.30E-01	8.87E-03	2.23E-01
ZMYM4	1.30E-01	6.53E-01	2.06E-03	6.45E-02	7.54E-03	2.03E-01
AQP4	1.89E-01	7.27E-01	2.52E-01	7.78E-01	2.86E-01	8.58E-01
ARID3B	3.09E-01	8.07E-01	1.01E-02	1.88E-01	1.07E-05	1.20E-03
AP1M2	3.72E-01	8.36E-01	8.62E-02	5.52E-01	6.02E-02	5.75E-01
FAM189B	2.01E-02	3.45E-01	9.96E-03	1.86E-01	1.44E-04	1.07E-02
ADAMTS8	7.77E-01	8.94E-01	7.61E-01	8.98E-01	9.26E-01	9.43E-01
MICA	8.58E-02	5.86E-01	7.32E-06	7.73E-04	4.43E-05	4.11E-03
PRSS33	8.54E-02	5.86E-01	5.50E-01	8.76E-01	2.61E-01	8.46E-01
DAP3	1.24E-05	1.70E-03	2.91E-04	1.49E-02	8.05E-07	1.26E-04
LINC01149	NA	NA	NA	NA	NA	NA
TAF6	3.23E-02	4.17E-01	4.64E-04	2.18E-02	1.09E-04	8.42E-03
GPANK1	6.27E-01	8.79E-01	4.74E-03	1.15E-01	1.73E-04	1.26E-02
LINC00310	NA	NA	NA	NA	NA	NA
RP11-503G7.2	NA	NA	NA	NA	NA	NA
FRYL	4.86E-02	4.86E-01	4.98E-04	2.30E-02	5.77E-01	8.95E-01
E2F1	3.85E-02	4.43E-01	1.45E-05	1.34E-03	1.38E-03	6.50E-02
PRRT1	2.61E-01	7.87E-01	4.30E-05	3.40E-03	7.33E-04	4.00E-02
IRF7	1.96E-02	3.40E-01	4.29E-01	8.55E-01	8.88E-03	2.23E-01
LINC00857	NA	NA	NA	NA	NA	NA
CERCAM	4.90E-03	1.62E-01	6.03E-02	4.77E-01	3.65E-01	8.77E-01
ZSWIM7	7.55E-02	5.64E-01	6.14E-05	4.51E-03	6.91E-04	3.86E-02
ADRA1A	1.93E-01	7.30E-01	2.05E-01	7.35E-01	5.36E-03	1.63E-01
NPY4R	NA	NA	NA	NA	3.19E-01	8.66E-01
TRMT61B	8.03E-02	5.73E-01	5.18E-04	2.36E-02	2.55E-01	8.43E-01

GJC2	1.42E-01	6.69E-01	9.35E-05	6.28E-03	8.93E-04	4.60E-02
PSMB2	8.61E-02	5.86E-01	2.61E-02	3.24E-01	2.28E-01	8.29E-01
PTGFR	5.96E-01	8.76E-01	7.00E-02	5.10E-01	7.14E-01	8.96E-01
VPS29	1.02E-01	6.22E-01	5.42E-04	2.46E-02	3.67E-01	8.78E-01
LINC00348	NA	NA	NA	NA	NA	NA
BRD2	6.66E-02	5.38E-01	1.03E-03	3.85E-02	2.37E-04	1.63E-02
UPF2	5.99E-02	5.23E-01	8.15E-01	9.04E-01	7.06E-01	8.96E-01
CHCHD7	6.42E-01	8.79E-01	3.45E-01	8.28E-01	2.31E-01	8.30E-01
HIST1H2BB	2.70E-04	2.21E-02	2.48E-02	3.15E-01	9.61E-01	9.65E-01
HSPE1	3.07E-01	8.06E-01	6.40E-05	4.63E-03	5.36E-04	3.15E-02
RP11-499E18.1	NA	NA	NA	NA	NA	NA
GCDH	7.77E-03	2.14E-01	5.99E-02	4.76E-01	4.58E-01	8.94E-01
ZNF146	4.40E-01	8.57E-01	3.90E-04	1.88E-02	1.83E-02	3.42E-01
CYP2C19	5.89E-01	8.75E-01	4.85E-01	8.69E-01	6.32E-02	5.84E-01
BNIP1	9.47E-02	6.05E-01	7.57E-04	3.16E-02	7.15E-01	8.97E-01
DR1	6.37E-04	4.21E-02	3.23E-03	8.79E-02	2.07E-05	2.20E-03
SUCLG2	2.68E-01	7.89E-01	2.35E-01	7.63E-01	2.77E-01	8.52E-01
TADA1	4.76E-01	8.59E-01	1.00E-01	5.82E-01	1.17E-01	7.08E-01
PRM3	7.52E-02	5.63E-01	2.09E-03	6.48E-02	2.59E-02	4.03E-01
PREX1	1.53E-06	2.94E-04	5.79E-03	1.30E-01	5.92E-01	8.95E-01
FAM96A	4.54E-01	8.58E-01	9.60E-05	6.31E-03	5.69E-02	5.65E-01
WFDC10A	1.14E-03	6.25E-02	2.90E-01	8.02E-01	7.55E-01	8.99E-01
AIP	3.62E-02	4.34E-01	8.15E-02	5.42E-01	4.53E-02	5.17E-01
PNRC1	7.10E-01	8.84E-01	4.91E-01	8.72E-01	1.61E-02	3.16E-01
XRCC3	3.65E-03	1.37E-01	2.92E-05	2.43E-03	1.87E-03	8.16E-02
LPCAT2	2.67E-01	7.89E-01	2.85E-01	8.00E-01	8.91E-02	6.55E-01
ATG16L2	1.27E-01	6.50E-01	2.60E-03	7.54E-02	7.13E-05	6.05E-03
ACSM4	4.30E-01	8.53E-01	1.17E-02	2.06E-01	3.12E-01	8.65E-01
ZNF311	6.34E-02	5.30E-01	5.01E-02	4.38E-01	2.26E-01	8.28E-01
HNMT	7.56E-02	5.65E-01	4.24E-01	8.54E-01	1.61E-01	7.73E-01
PDLIM4	2.94E-02	4.02E-01	1.04E-04	6.72E-03	2.28E-03	9.26E-02
ILF3-AS1	NA	NA	NA	NA	NA	NA
PPP1R2	1.08E-01	6.31E-01	4.27E-01	8.55E-01	1.73E-01	7.89E-01
REEP4	1.85E-01	7.22E-01	8.38E-03	1.67E-01	6.57E-01	8.96E-01
RPN1	6.30E-01	8.79E-01	1.26E-01	6.27E-01	1.46E-01	7.51E-01
LPIN3	9.06E-03	2.34E-01	8.05E-02	5.40E-01	4.03E-03	1.32E-01
ARHGEF38	1.54E-01	6.86E-01	1.44E-06	1.95E-04	1.49E-07	2.81E-05
AP4M1	1.25E-02	2.74E-01	1.40E-04	8.47E-03	6.60E-05	5.71E-03
ZSCAN23	1.59E-02	3.11E-01	3.68E-03	9.66E-02	2.20E-02	3.77E-01
ZNF84	NA	NA	NA	NA	NA	NA
LMAN1L	2.12E-02	3.50E-01	1.17E-04	7.33E-03	9.87E-02	6.75E-01
PROB1	8.66E-04	5.19E-02	1.80E-01	7.04E-01	3.99E-03	1.32E-01
RP11-16E12.2	NA	NA	NA	NA	NA	NA
BMP8A	8.91E-03	2.33E-01	1.92E-04	1.07E-02	5.35E-04	3.15E-02
GSDMB	1.92E-06	3.48E-04	4.81E-07	7.14E-05	8.21E-05	6.66E-03

UNC5C	6.69E-01	8.81E-01	8.16E-01	9.04E-01	8.38E-01	9.09E-01
RASSF3	4.85E-01	8.61E-01	8.45E-03	1.67E-01	1.13E-01	7.01E-01
IMPG1	4.21E-01	8.50E-01	6.78E-01	8.88E-01	7.18E-01	8.97E-01
NDUFC1	8.79E-01	9.21E-01	3.28E-03	8.89E-02	1.76E-01	7.92E-01
DKFZP434L187	NA	NA	NA	NA	NA	NA
FAM126A	9.12E-03	2.34E-01	1.35E-02	2.23E-01	2.74E-02	4.17E-01
CPLX3	2.14E-02	3.52E-01	3.17E-04	1.59E-02	1.28E-01	7.26E-01
IKZF3	7.13E-02	5.52E-01	3.05E-02	3.50E-01	8.42E-02	6.43E-01
KIAA0907	2.08E-01	7.45E-01	1.09E-01	5.99E-01	6.86E-05	5.84E-03
ZKSCAN4	5.41E-04	3.76E-02	5.63E-04	2.51E-02	1.20E-02	2.65E-01
LKAAEAR1	3.26E-01	8.17E-01	2.60E-03	7.54E-02	5.10E-01	8.95E-01
DYNC1H1	7.36E-04	4.65E-02	3.28E-02	3.61E-01	1.49E-01	7.55E-01
GNGT2	6.45E-01	8.79E-01	1.14E-01	6.09E-01	3.74E-01	8.80E-01
CDC42EP3	5.84E-01	8.74E-01	4.61E-01	8.64E-01	5.92E-01	8.95E-01
NAA20	1.67E-01	7.03E-01	7.20E-02	5.16E-01	5.52E-01	8.95E-01
MS4A4A	5.24E-01	8.67E-01	6.26E-01	8.84E-01	4.16E-01	8.89E-01
OBSCN	2.72E-02	3.87E-01	1.33E-05	1.27E-03	6.37E-03	1.82E-01
C1orf145	NA	NA	NA	NA	NA	NA
CDH16	3.44E-01	8.25E-01	1.24E-01	6.22E-01	1.26E-03	6.02E-02
USP49	1.36E-02	2.87E-01	9.58E-05	6.31E-03	6.20E-02	5.82E-01
SNX19	1.86E-04	1.68E-02	1.33E-04	8.14E-03	8.77E-03	2.23E-01
FBXL18	9.07E-01	9.34E-01	3.28E-04	1.63E-02	1.80E-01	7.94E-01
VWA7	2.84E-01	7.95E-01	2.88E-02	3.42E-01	7.36E-03	2.01E-01
NAA15	8.58E-01	9.14E-01	2.45E-03	7.23E-02	1.43E-01	7.46E-01
DHX30	1.53E-02	3.05E-01	1.37E-04	8.33E-03	2.81E-01	8.56E-01
PCMTD1	5.07E-01	8.63E-01	8.78E-04	3.50E-02	6.73E-04	3.82E-02
HLA-DOA	7.68E-03	2.13E-01	6.61E-04	2.86E-02	2.56E-04	1.73E-02
RFTN2	4.43E-01	8.57E-01	1.89E-05	1.67E-03	2.34E-05	2.43E-03
ALKBH1	5.49E-01	8.69E-01	8.86E-03	1.73E-01	1.64E-01	7.77E-01
LDLR	7.73E-01	8.94E-01	1.55E-01	6.68E-01	7.17E-02	6.14E-01
DSCAML1	3.76E-01	8.37E-01	5.52E-02	4.58E-01	3.57E-01	8.77E-01
LCTL	6.90E-01	8.82E-01	1.64E-01	6.80E-01	1.05E-02	2.46E-01
HINT1	7.14E-01	8.84E-01	4.08E-02	4.01E-01	2.17E-04	1.50E-02
MAP2	7.17E-02	5.53E-01	5.75E-03	1.30E-01	4.84E-02	5.28E-01
STAG3	3.27E-02	4.17E-01	9.97E-04	3.78E-02	1.61E-02	3.16E-01
IRF4	5.48E-02	5.08E-01	2.69E-01	7.88E-01	3.97E-01	8.86E-01
PKP2	1.86E-01	7.25E-01	4.58E-01	8.63E-01	8.73E-01	9.19E-01
EPB41L2	3.86E-02	4.44E-01	8.54E-04	3.44E-02	1.37E-02	2.85E-01
MSH5	3.53E-01	8.28E-01	2.86E-02	3.41E-01	2.58E-03	1.00E-01

Note: NA represents not applicable.

Supplementary Table S11. The 23 common pathways significantly enriched by three S-MultiXcan gene sets

Pathway ID	Pathway name	Susceptible COVID-	Susceptible	Hospitalized COVID-	Hospitalized	Very severe	Very severe

		19 (P-value)	COVID-19 (FDR)	19 (P-value)	COVID-19 (FDR)	COVID-19 (P-value)	COVID-19 (FDR)
hsa05169	Epstein-Barr virus infection	3.48E-04	2.83E-02	8.00E-11	2.61E-08	1.45E-09	4.71E-07
hsa05168	Herpes simplex infection	1.12E-03	7.33E-02	1.37E-09	1.49E-07	3.35E-09	5.46E-07
hsa05164	Influenza A	1.69E-02	2.76E-01	1.90E-07	1.24E-05	8.98E-09	9.76E-07
hsa04940	Type I diabetes mellitus	6.25E-03	2.04E-01	1.72E-07	1.24E-05	9.41E-08	7.67E-06
hsa05167	Kaposi sarcoma-associated herpesvirus infection	3.16E-05	3.90E-03	1.81E-10	2.94E-08	2.03E-07	1.32E-05
hsa05320	Autoimmune thyroid disease	1.11E-02	2.58E-01	9.29E-07	5.05E-05	5.14E-07	2.79E-05
hsa05330	Allograft rejection	4.41E-03	1.80E-01	1.13E-06	5.28E-05	6.70E-07	3.12E-05
hsa05332	Graft-versus-host disease	5.46E-03	1.98E-01	1.95E-06	7.82E-05	1.15E-06	4.35E-05
hsa05416	Viral myocarditis	1.49E-02	2.76E-01	2.16E-06	7.82E-05	1.20E-06	4.35E-05
hsa04060	Cytokine-cytokine receptor interaction	7.59E-06	2.48E-03	1.04E-04	2.11E-03	2.11E-06	6.89E-05
hsa05145	Toxoplasmosis	1.67E-02	2.76E-01	1.32E-03	2.14E-02	2.09E-05	6.18E-04
hsa04514	Cell adhesion molecules (CAMs)	3.75E-02	4.71E-01	5.02E-05	1.09E-03	2.57E-05	6.97E-04
hsa04145	Phagosome	2.09E-03	9.72E-02	1.40E-05	3.81E-04	4.09E-05	9.72E-04
hsa05160	Hepatitis C	2.78E-02	4.09E-01	6.97E-04	1.33E-02	7.24E-05	1.57E-03
hsa04612	Antigen processing and presentation	3.00E-02	4.09E-01	1.64E-05	4.12E-04	8.25E-05	1.68E-03
hsa04672	Intestinal immune network for IgA production	8.98E-03	2.44E-01	5.84E-03	7.61E-02	5.25E-04	8.56E-03
hsa05310	Asthma	3.01E-02	4.09E-01	1.06E-02	1.12E-01	7.97E-04	1.24E-02
hsa05163	Human cytomegalovirus infection	1.38E-02	2.76E-01	5.21E-06	1.55E-04	9.97E-04	1.48E-02
hsa04621	NOD-like receptor signaling pathway	1.58E-02	2.76E-01	8.35E-04	1.43E-02	2.10E-03	2.74E-02
hsa00601	Glycosphingolipid biosynthesis	1.64E-03	8.89E-02	7.19E-03	8.69E-02	5.81E-03	6.53E-02
hsa05203	Viral carcinogenesis	3.14E-02	4.09E-01	9.85E-03	1.12E-01	6.28E-03	6.82E-02
hsa04062	Chemokine signaling pathway	3.59E-05	3.90E-03	6.90E-03	8.65E-02	1.50E-02	1.40E-01
hsa04151	PI3K-Akt signaling pathway	1.19E-02	2.58E-01	1.56E-02	1.50E-01	2.32E-02	2.04E-01

Supplementary Table S12. The 30 significant pathways enriched by the S-MultiXcan-identified 438 genes associated with one of three COVID-19 outcomes

Gene Set	Description	Enrichment ratio	P Value	FDR
hsa05169	Epstein-Barr virus infection	5.20	2.04E-11	6.64E-09
hsa05168	Herpes simplex infection	5.18	1.64E-10	1.98E-08
hsa05167	Kaposi sarcoma-associated herpesvirus infection	5.15	1.82E-10	1.98E-08
hsa04940	Type I diabetes mellitus	11.14	2.06E-09	1.68E-07
hsa05330	Allograft rejection	11.46	8.47E-09	5.52E-07
hsa05332	Graft-versus-host disease	10.62	1.89E-08	1.03E-06
hsa05320	Autoimmune thyroid disease	9.04	2.23E-08	1.04E-06
hsa05164	Influenza A	4.58	5.87E-08	2.39E-06
hsa05416	Viral myocarditis	8.12	7.28E-08	2.64E-06

hsa04145	Phagosome	4.58	3.34E-07	1.09E-05
hsa04612	Antigen processing and presentation	6.22	1.22E-06	3.60E-05
hsa05166	Human T-cell leukemia virus 1 infection	3.07	2.01E-05	5.45E-04
hsa04514	Cell adhesion molecules (CAMs)	3.93	2.40E-05	6.02E-04
hsa05150	Staphylococcus aureus infection	6.22	3.64E-05	8.47E-04
hsa05145	Toxoplasmosis	4.28	4.77E-05	1.04E-03
hsa05310	Asthma	8.43	6.13E-05	1.25E-03
hsa04672	Intestinal immune network for IgA production	6.22	1.13E-04	2.17E-03
hsa04060	Cytokine-cytokine receptor interaction	2.67	1.30E-04	2.35E-03
hsa05163	Human cytomegalovirus infection	2.90	1.93E-04	3.22E-03
hsa05160	Hepatitis C	3.66	1.98E-04	3.22E-03
hsa05140	Leishmaniasis	4.71	2.72E-04	4.22E-03
hsa04659	Th17 cell differentiation	3.66	7.48E-04	1.11E-02
hsa04650	Natural killer cell mediated cytotoxicity	3.32	8.23E-04	1.17E-02
hsa05323	Rheumatoid arthritis	3.87	1.02E-03	1.39E-02
hsa04658	Th1 and Th2 cell differentiation	3.79	1.18E-03	1.54E-02
hsa04621	NOD-like receptor signaling pathway	2.85	1.62E-03	2.01E-02
hsa04640	Hematopoietic cell lineage	3.59	1.67E-03	2.01E-02
hsa00601	Glycosphingolipid biosynthesis	6.45	3.12E-03	3.63E-02
hsa05162	Measles	2.97	3.25E-03	3.65E-02
hsa05321	Inflammatory bowel disease (IBD)	4.02	3.62E-03	3.93E-02
hsa05203	Viral carcinogenesis	2.38	6.43E-03	6.76E-02
hsa04062	Chemokine signaling pathway	2.30	1.15E-02	1.11E-01
hsa04151	PI3K-Akt signaling pathway	1.72	3.27E-02	2.74E-01

Supplementary Table S13. LDSC analysis identifies the genetic correlations between three COVID-19 phenotypes and 66 complex diseases or traits

Phenotypes	Susceptible COVID-19		Hospitalized COVID-19		Very severe COVID-19	
	r_g	P value	r_g	P value	r_g	P value
Anorexia nervosa	0.010	8.97E-01	-0.129	4.29E-02	-0.131	4.05E-02
Autism spectrum disorder	-0.063	2.32E-01	-0.062	1.81E-01	-0.062	1.94E-01
Bipolar disorder	-0.063	1.40E-01	-0.039	2.27E-01	-0.060	9.97E-02
Attention deficit hyperactivity disorder	0.224	3.12E-07	0.248	3.12E-08	0.181	1.11E-05
Major Depression Disease	0.088	1.07E-01	0.097	5.05E-02	0.070	1.46E-01
Schizophrenia	0.043	1.32E-01	-0.012	6.17E-01	0.004	8.86E-01
Insomnia	-0.185	5.93E-06	-0.181	7.12E-05	-0.102	2.75E-02
Multiple sclerosis	0.074	1.35E-01	0.136	8.84E-03	0.106	5.73E-02
Migraine	-0.049	3.74E-01	0.019	7.03E-01	0.016	7.56E-01
Ischemic stroke	0.082	1.82E-01	0.206	9.73E-05	0.189	1.23E-03
Epilepsy	-0.059	5.01E-01	-0.046	5.24E-01	-0.108	1.39E-01
Focal epilepsy	-0.092	5.56E-01	-0.044	7.29E-01	-0.104	4.14E-01
Generalized epilepsy	-0.040	5.75E-01	-0.054	3.48E-01	-0.120	4.01E-02

Juvenile myoclonic epilepsy	-0.079	3.32E-01	-0.081	2.56E-01	-0.123	9.90E-02
Alzheimer’s disease	0.219	4.90E-02	0.067	3.96E-01	0.052	5.47E-01
Parkinson’s disease	-0.022	7.06E-01	-0.031	5.97E-01	-0.032	5.94E-01
Snoring	0.110	1.85E-03	0.131	5.87E-05	0.140	6.08E-05
Daytime Napping	0.132	1.47E-02	0.158	5.38E-03	0.153	1.53E-02
Happiness	-0.094	8.72E-02	-0.005	9.09E-01	0.006	8.98E-01
Cigarettes smoked per day	0.131	1.05E-03	0.200	7.56E-09	0.177	2.31E-06
Extraversion	-0.094	8.72E-02	-0.005	9.09E-01	0.006	8.98E-01
Subjective well-being	-0.014	7.84E-01	0.018	7.19E-01	0.020	7.37E-01
Conscientiousness	0.116	3.46E-01	0.040	7.03E-01	-0.003	9.81E-01
Openness to experience	-0.162	1.27E-01	-0.113	2.23E-01	-0.045	6.20E-01
Neuroticism	0.022	5.06E-01	0.078	1.09E-02	0.084	1.70E-02
Cognitive Performance	-0.298	4.96E-18	-0.216	1.23E-11	-0.138	3.47E-05
Smoking cesstion	-0.104	2.07E-01	-0.016	8.25E-01	-0.050	5.20E-01
Age of smoking initiation	0.074	1.35E-01	0.136	8.84E-03	0.106	5.73E-02
Hypertension	0.153	7.86E-06	0.187	4.74E-09	0.155	1.12E-05
Systolic blood pressure	-0.049	9.17E-02	0.005	8.45E-01	-0.006	8.02E-01
Pulse pressure	0.154	1.33E-02	0.084	1.39E-01	0.086	1.33E-01
High blood pressure	0.148	1.19E-05	0.186	1.56E-08	0.154	1.86E-05
Diastolic blood pressure (East Asian)	0.091	1.40E-01	0.037	4.85E-01	0.087	1.34E-01
Diastolic blood pressure (European)	-0.038	2.22E-01	0.007	7.66E-01	-0.006	8.21E-01
Diastolic blood pressure (Hispanic)	0.047	5.86E-01	0.048	4.91E-01	0.031	6.61E-01
Coronary artery disease	0.152	2.83E-04	0.205	9.12E-07	0.125	4.21E-03
Type II diabetes	0.174	6.20E-06	0.282	3.24E-09	0.241	3.93E-06
Ulcerative Colitis	-0.072	1.72E-01	0.004	9.31E-01	-0.013	7.95E-01
Psoriasis	0.091	3.31E-01	0.045	5.50E-01	-0.047	5.54E-01
Coeliac disease	0.007	9.43E-01	0.015	8.70E-01	-0.054	6.20E-01
Primary biliary cholangitis	0.024	7.44E-01	0.131	7.82E-02	0.121	1.34E-01
Rheumatoid arthritis	0.144	2.68E-03	0.134	1.49E-03	0.088	6.42E-02
Inflammatory bowel disease	-0.071	1.14E-01	-0.002	9.59E-01	-0.035	3.55E-01
Systemic lupus erythematosus	-0.020	7.82E-01	0.079	2.14E-01	0.094	1.44E-01
Waist Circumference	0.260	5.73E-10	0.330	7.64E-10	0.324	5.56E-07
Total cholesterol	-0.106	3.94E-02	-0.107	1.58E-02	-0.083	8.94E-02
Chronic kidney disease	0.096	4.03E-01	-0.004	9.64E-01	-0.022	8.21E-01

Body fat percentage	0.281	1.49E-18	0.362	1.31E-15	0.338	2.77E-09
Triglyceride level	0.014	8.74E-01	0.164	3.27E-02	0.142	9.41E-02
High-density lipoprotein (HDL) cholesterol	-0.159	2.42E-06	-0.196	8.91E-09	-0.178	1.77E-05
Low-density lipoprotein (LDL) cholesterol	-0.039	2.99E-01	-0.019	5.96E-01	-0.021	5.79E-01
Tuberculosis	0.429	3.50E-01	0.013	9.57E-01	0.267	4.13E-01
Adult asthma	0.080	8.54E-02	-0.015	7.05E-01	-0.041	3.20E-01
Small cell lung cancer	0.024	9.12E-01	0.119	4.99E-01	0.051	7.69E-01
Pulmonary embolism	0.248	1.70E-02	0.222	3.16E-02	0.221	2.51E-02
Pulmonary artery	0.218	1.36E-01	0.398	3.16E-02	0.417	7.17E-03
Non-small cell lung cancer, squamous	0.091	6.09E-01	0.306	3.16E-02	0.075	6.04E-01
Non-small cell lung cancer, adenocarcinoma	0.077	5.85E-01	0.255	3.16E-02	0.229	9.65E-02
Non-small cell lung cancer	0.066	4.80E-01	0.191	3.16E-02	0.174	6.32E-02
Lung volume	-0.058	6.04E-02	-0.066	6.22E-05	-0.037	2.43E-01
Lung function (FVC)	0.059	5.77E-02	0.180	6.22E-05	0.211	1.42E-07
Influenza without pneumonia	0.220	2.27E-01	0.173	2.06E-01	0.079	5.43E-01
Influenza with pneumonia	0.180	1.56E-02	0.254	1.08E-02	0.220	6.94E-04
All influenza	0.196	1.32E-01	0.225	1.08E-02	0.106	2.85E-01
Emphysema/chronic bronchitis	0.149	5.38E-02	0.169	1.08E-02	0.164	1.98E-02
Chronic obstructive pulmonary disease	0.229	1.00E-02	0.193	1.11E-02	0.146	8.15E-02

Supplementary Table S14. LDSC analysis identifies the genetic correlations between three COVID-19 phenotypes and 24 cancers

Cancers	Susceptible COVID-19		Hospitalized COVID-19		Very severe COVID-19	
	r_g	P value	r_g	P value	r_g	P value
Endometrial Carcinoma	0.09	2.55E-01	0.25	1.31E-02	0.29	2.18E-04
Adnexa	-0.13	4.76E-01	0.48	9.76E-01	-0.06	7.00E-01
Bladder	0.18	2.22E-01	0.22	4.91E-01	0.14	2.61E-01
Brain	-0.11	6.48E-01	0.65	8.76E-01	0.05	8.22E-01
Breast	-0.02	7.38E-01	0.74	9.75E-02	0.05	3.33E-01
Cervix Uteri	0.05	8.12E-01	0.81	5.47E-01	0.05	7.96E-01
Colon	0.15	3.03E-01	0.30	9.70E-02	0.15	2.59E-01
Endocrine	-0.02	8.86E-01	0.89	1.26E-02	-0.21	5.24E-02
Kidney Notrenalpelvis	0.04	8.27E-01	0.83	4.44E-01	0.26	1.04E-01

Liver Intrahepatic Bile Ducts	0.28	1.78E-01	0.18	3.63E-01	0.18	3.77E-01
Lung Non-small cancer	0.07	5.40E-01	0.54	1.14E-01	0.16	7.24E-02
Male Genital	-0.12	1.06E-01	0.11	8.58E-01	0.09	1.28E-01
Ovary	-0.04	7.92E-01	0.79	9.11E-01	-0.06	6.03E-01
Prostate	-0.13	7.52E-02	0.08	8.65E-01	0.09	1.26E-01
Rectum	0.00	9.80E-01	0.98	6.50E-01	-0.14	2.05E-01
Small Intestine	0.05	7.39E-01	0.74	4.70E-01	0.05	7.42E-01
Thyroid Gland	-0.04	7.17E-01	0.72	7.74E-03	-0.21	3.50E-02
Urinary Tract	0.11	3.97E-01	0.40	4.37E-01	0.19	8.57E-02
Colorectal	-0.14	1.32E-01	0.13	6.80E-01	0.01	9.17E-01
Glioma	0.13	4.16E-01	0.42	1.96E-01	0.14	3.59E-01
Head Neck	0.50	5.09E-01	0.51	4.98E-01	0.55	5.03E-01
Lung	0.06	5.82E-01	0.58	5.07E-01	0.04	7.28E-01
Melanoma	-0.08	3.97E-01	0.40	1.39E-01	-0.18	2.55E-02
Kidney	0.47	9.58E-02	0.10	5.23E-01	-0.12	6.21E-01

Supplementary Table S15. Significant pathways enriched by up-regulated genes in MSC positive cells

Description	Gene size	Enrichment ratio	P Value	FDR
Parathyroid hormone synthesis, secretion and action	106	2.13	3.03E-03	2.47E-02
Herpes simplex infection	185	1.83	2.73E-03	2.28E-02
Influenza A	171	1.90	2.07E-03	1.79E-02
IL-17 signaling pathway	93	2.28	2.09E-03	1.79E-02
Relaxin signaling pathway	130	2.06	1.90E-03	1.75E-02
Viral carcinogenesis	201	1.83	1.93E-03	1.75E-02
Fluid shear stress and atherosclerosis	139	2.03	1.77E-03	1.69E-02
Ferroptosis	40	3.18	1.57E-03	1.56E-02
Melanoma	72	2.55	1.43E-03	1.46E-02
Transcriptional misregulation in cancer	186	1.90	1.36E-03	1.43E-02
Th17 cell differentiation	107	2.24	1.29E-03	1.40E-02
Regulation of actin cytoskeleton	213	1.86	1.02E-03	1.15E-02
Renal cell carcinoma	69	2.66	9.50E-04	1.11E-02
Cellular senescence	160	2.03	8.36E-04	1.01E-02
TNF signaling pathway	110	2.31	6.54E-04	8.20E-03
Hippo signaling pathway	154	2.11	4.84E-04	6.31E-03
Prostate cancer	97	2.47	4.05E-04	5.94E-03
MAPK signaling pathway	295	1.77	4.28E-04	5.94E-03
Adherens junction	72	2.75	4.37E-04	5.94E-03
Human T-cell leukemia virus 1 infection	255	1.88	2.33E-04	3.77E-03
Human cytomegalovirus infection	225	1.95	2.43E-04	3.77E-03
EGFR tyrosine kinase inhibitor resistance	79	2.86	1.05E-04	1.97E-03

Amoebiasis	96	2.65	1.14E-04	1.97E-03
Malaria	49	3.46	1.15E-04	1.97E-03
JAK-STAT signaling pathway	162	2.27	6.27E-05	1.32E-03
Hepatitis B	144	2.35	6.47E-05	1.32E-03
FoxO signaling pathway	132	2.46	4.48E-05	1.04E-03
Osteoclast differentiation	128	2.54	2.70E-05	6.76E-04
TGF-beta signaling pathway	84	3.03	1.76E-05	4.77E-04
Signaling pathways regulating pluripotency of stem cells	139	2.64	3.78E-06	1.17E-04
Proteoglycans in cancer	201	2.32	3.96E-06	1.17E-04
ECM-receptor interaction	82	3.27	3.03E-06	1.10E-04
AGE-RAGE signaling pathway in diabetic complications	99	3.14	1.08E-06	4.40E-05
Protein digestion and absorption	90	3.29	8.08E-07	3.76E-05
Kaposi sarcoma-associated herpesvirus infection	186	2.51	6.63E-07	3.60E-05
PI3K-Akt signaling pathway	354	2.15	4.07E-08	2.65E-06
Focal adhesion	199	2.77	3.48E-09	7.87E-07
Human papillomavirus infection	339	2.25	8.77E-09	7.87E-07
Pathways in cancer	526	1.96	9.26E-09	7.87E-07
Protein processing in endoplasmic reticulum	165	2.91	9.66E-09	7.87E-07

Supplementary Table S16. scDrugHunter-identified top-ranked gene-drug interaction pairs in lung MSC positive cells (scDGS > 120)

Gene	Drug	Gene specificity rank	Gene correlation rank	Gene-drug interaction rank	SMultiXcan-based gene P value rank	scDGS
CCR1	CCX354	9.42	9.49	9.98	9.88	187.67
CCR1	CHEMBL2205805	9.42	9.49	9.91	9.88	187.03
CCR1	BMS-817399	9.42	9.49	9.91	9.88	187.03
CCR1	AZD4818	9.42	9.49	9.70	9.88	185.02
CCR1	TERPYRIDINE	9.42	9.49	9.18	9.88	180.10
TNFRSF4	IVUXOLIMAB	9.36	9.54	9.86	8.31	171.71
TNFRSF4	VONLEROLIZUMA B	9.36	9.54	9.86	8.31	171.71
TNFRSF4	TAVOLIMAB	9.36	9.54	9.86	8.31	171.71
PDE4A	DROTAVERINE	9.13	9.38	9.22	9.21	170.59
PDE4A	ENPROFYLLINE	9.13	9.38	9.11	9.21	169.61
PDE4A	APREMILAST	9.13	9.38	8.92	9.21	167.86
PDE4A	ROFLUMILAST	9.13	9.38	8.88	9.21	167.47
PDE4A	DYPHYLLINE	9.13	9.38	8.77	9.21	166.43
PDE4A	AROFYLLINE	9.13	9.38	8.70	9.21	165.75
PDE4A	OGLEMILAST	9.13	9.38	8.70	9.21	165.75
PDE4A	CHEMBL1229585	9.13	9.38	8.70	9.21	165.75
PDE4A	LOTAMILAST	9.13	9.38	8.70	9.21	165.75
PDE4A	CRISABOROLE	9.13	9.38	8.56	9.21	164.49
PDE4A	PICLAMILAST	9.13	9.38	8.53	9.21	164.19
PDE4A	OXTRIPHYLLINE	9.13	9.38	8.49	9.21	163.89
PDE4A	REVAMILAST	9.13	9.38	8.43	9.21	163.28
PDE4A	MK-0873	9.13	9.38	8.43	9.21	163.28
PDE4A	LIRIMILAST	9.13	9.38	8.43	9.21	163.28

PDE4A	CHEMBL74078	9.13	9.38	8.43	9.21	163.28
PDE4A	IBUDILAST	9.13	9.38	8.24	9.21	161.56
PDE4A	TOFISOPAM	9.13	9.38	8.01	9.21	159.37
PDE4A	MILRINONE	9.13	9.38	8.01	9.21	159.37
PDE4A	TOFIMILAST	9.13	9.38	7.86	9.21	157.98
PDE4A	ROLIPRAM	9.13	9.38	7.86	9.21	157.98
PDE4A	DENBUFYLLINE	9.13	9.38	7.86	9.21	157.98
PDE4A	HT-0712	9.13	9.38	7.86	9.21	157.98
PDE4A	GSK-356278	9.13	9.38	7.86	9.21	157.98
PDE4A	PROPENTOFYLLINE	9.13	9.38	7.86	9.21	157.98
PDE4A	ILOPROST	9.13	9.38	7.86	9.21	157.98
PDE4A	THEOPHYLLINE	9.13	9.38	7.64	9.21	156.00
IFNAR2	INTERFERON ALFA-N3	5.81	9.95	9.83	9.93	155.66
PDE4A	CDC-801	9.13	9.38	7.48	9.21	154.47
PDE4A	CDP840	9.13	9.38	7.48	9.21	154.47
PDE4A	TADALAFIL	9.13	9.38	7.48	9.21	154.47
VIPR2	SECRETIN	9.47	8.49	9.65	7.55	154.45
IFNAR2	INTERFERON ALFA-2B	5.81	9.95	9.67	9.93	154.41
VIPR2	VASOACTIVE INTESTINAL PEPTIDE	9.47	8.49	9.54	7.55	153.47
IFNAR2	PEGINTERFERON ALFA-2A	5.81	9.95	9.00	9.93	149.12
IFNAR2	NYLIDRIN	5.81	9.95	9.00	9.93	149.12
IFNAR2	INTERFERON ALFACON-1	5.81	9.95	9.00	9.93	149.12
IFNAR2	PEGINTERFERON BETA-1A	5.81	9.95	9.00	9.93	149.12
IFNAR2	INTERFERON BETA-1B	5.81	9.95	9.00	9.93	149.12
IFNAR2	ANIFROLUMAB	5.81	9.95	9.00	9.93	149.12
IFNAR2	ALBINTERFERON ALFA-2B	5.81	9.95	9.00	9.93	149.12
IFNAR2	PEGINTERFERON ALFA-2B	5.81	9.95	9.00	9.93	149.12
IFNAR2	INTERFERON BETA-1A	5.81	9.95	9.00	9.93	149.12
IFNAR2	INTERFERON ALFA-2A	5.81	9.95	9.00	9.93	149.12
PDE4A	FLAVOXATE HYDROCHLORIDE	9.13	9.38	6.85	9.21	148.67
PDE4A	PENTOXIFYLLINE	9.13	9.38	6.80	9.21	148.18
PDE4A	THEOPHYLLINE SODIUM GLYCINATE	9.13	9.38	6.65	9.21	146.81
PDE4A	INAMRINONE	9.13	9.38	6.65	9.21	146.81
IFNAR2	SIFALIMUMAB	5.81	9.95	8.68	9.93	146.53
P4HA2	PROLINE	8.41	8.15	9.59	8.09	146.42
PDE4A	AMINOPHYLLINE	9.13	9.38	6.60	9.21	146.37
PDE4A	DIPYRIDAMOLE	9.13	9.38	6.60	9.21	146.37

SLC5A3	PHLORIZIN	7.25	8.33	9.94	8.57	144.28
PDE4A	ETAZOLATE	9.13	9.38	6.16	9.21	142.29
PDE4A	AMLEXANOX	9.13	9.38	6.06	9.21	141.39
SENP7	CHEMBL378903	7.69	7.61	8.55	9.68	139.46
LTA	PATECLIZUMAB	6.77	8.17	9.70	8.63	136.90
SENP7	XANTHOANGELO L B	7.69	7.61	8.18	9.68	136.58
SENP7	CHEMBL546865	7.69	7.61	7.99	9.68	135.16
SENP7	CHEMBL584668	7.69	7.61	7.99	9.68	135.16
LTA	BAMINERCEPT	6.77	8.17	9.45	8.63	135.06
SENP7	DIHYDROXANTH OHUMOL	7.69	7.61	7.89	9.68	134.39
SENP7	CHEMBL546170	7.69	7.61	7.89	9.68	134.39
SENP7	CHEMBL412603	7.69	7.61	7.71	9.68	132.99
SENP7	CHEMBL484663	7.69	7.61	7.71	9.68	132.99
TYK2	TOFACITINIB	7.89	6.94	8.39	9.54	132.87
LTA	ETANERCEPT	6.77	8.17	9.13	8.63	132.68
TYK2	BREPOCITINIB	7.89	6.94	8.30	9.54	132.21
SENP7	CHEMBL1493528	7.69	7.61	7.55	9.68	131.79
SENP7	CHEMBL602561	7.69	7.61	7.55	9.68	131.79
SENP7	CHEMBL600313	7.69	7.61	7.55	9.68	131.79
COL11A2	COLLAGENASE CLOSTRIDIUM HISTOLYTICUM	9.44	7.23	8.45	7.30	131.33
HCN3	ZATEBRADINE	8.15	7.02	9.38	7.91	131.06
TYK2	PEFICITINIB	7.89	6.94	8.14	9.54	130.99
TYK2	CHEMBL21156	7.89	6.94	8.14	9.54	130.99
HCN3	CILOBRADINE	8.15	7.02	9.32	7.91	130.63
HCN3	IVABRADINE	8.15	7.02	9.23	7.91	129.94
SENP7	CHEMBL576208	7.69	7.61	7.30	9.68	129.87
PLEKHA4	CHEMBL23552	8.13	5.35	9.89	9.35	129.67
SENP7	IPRIFLAVONE	7.69	7.61	7.23	9.68	129.35
COL11A2	OCRIPLASMIN	9.44	7.23	8.20	7.30	129.26
CLK2	LORECIVIVINT	7.77	7.80	8.54	8.01	128.75
SENP7	CHEMBL1504679	7.69	7.61	7.13	9.68	128.62
SENP7	CHEMBL428789	7.69	7.61	7.03	9.68	127.86
LTA	ABACAVIR	6.77	8.17	8.46	8.63	127.64
TYK2	TOFACITINIB CITRATE	7.89	6.94	7.64	9.54	127.33
TYK2	OCLACITINIB	7.89	6.94	7.64	9.54	127.33
TYK2	SOLCITINIB	7.89	6.94	7.64	9.54	127.33
TYK2	BMS-911543	7.89	6.94	7.64	9.54	127.33
TYK2	DELGOCITINIB	7.89	6.94	7.64	9.54	127.33
TYK2	NS-018	7.89	6.94	7.64	9.54	127.33
TYK2	FILGOTINIB	7.89	6.94	7.64	9.54	127.33
CA11	ZONISAMIDE	7.86	6.21	9.63	8.44	127.12
SENP7	CHEMBL299853	7.69	7.61	6.89	9.68	126.73
TYK2	CERDULATINIB	7.89	6.94	7.52	9.54	126.38
IMPG2	HYALURONATE SODIUM	8.58	5.32	9.57	8.62	126.34

SLC22A4	LEVOCARNITINE	9.12	4.41	9.72	8.84	125.60
CLK2	LEUCETTAMINE B	7.77	7.80	8.06	8.01	125.06
TYK2	FEDRATINIB	7.89	6.94	7.19	9.54	123.97
TYK2	UPADACITINIB	7.89	6.94	7.19	9.54	123.97
LTA	CARBAMAZEPINE	6.77	8.17	7.79	8.63	122.63
TYK2	BARICITINIB	7.89	6.94	6.96	9.54	122.24
PTGFR	TRAVOPROST	9.34	9.14	9.98	3.20	121.78
SACM1L	BUPROPION	8.19	4.97	9.47	8.99	121.51
CLK2	GATIFLOXACIN	7.77	7.80	7.52	8.01	120.81
PTGFR	LATANOPROST	9.34	9.14	9.86	3.20	120.60
TYK2	ZOTIRACICLIB	7.89	6.94	6.73	9.54	120.56
PTGFR	TAFLUPROST	9.34	9.14	9.83	3.20	120.36
PTGFR	BIMATOPROST	9.34	9.14	9.82	3.20	120.25

Supplementary Table S17. Significant pathways enriched by up-regulated genes in intestinal tuft positive cells

Description	Gene size	Enrichment ratio	P Value	FDR
Ribosome	153	6.02	<2.2e-16	2.20E-16
Relaxin signaling pathway	130	3.22	3.04E-06	4.23E-04
TNF signaling pathway	110	3.42	3.89E-06	4.23E-04
Human cytomegalovirus infection	225	2.23	1.74E-04	1.42E-02
Mitophagy	65	3.54	2.23E-04	1.45E-02
Protein digestion and absorption	90	3.02	3.17E-04	1.72E-02
IL-17 signaling pathway	93	2.92	4.39E-04	2.05E-02
Focal adhesion	199	2.21	5.10E-04	2.08E-02
Kaposi sarcoma-associated herpesvirus infection	186	2.14	1.38E-03	5.00E-02
Fluid shear stress and atherosclerosis	139	2.26	2.53E-03	0.076
AGE-RAGE signaling pathway in diabetic complications	99	2.54	2.55E-03	0.076
Legionellosis	55	3.04	4.28E-03	0.111
JAK-STAT signaling pathway	162	2.07	4.50E-03	0.111
cGMP-PKG signaling pathway	163	2.05	4.78E-03	0.111
Prostate cancer	97	2.37	6.33E-03	0.138
Osteoclast differentiation	128	2.12	7.96E-03	0.156
Cocaine addiction	49	2.99	8.15E-03	0.156
Human T-cell leukemia virus 1 infection	255	1.72	1.03E-02	0.180
PI3K-Akt signaling pathway	354	1.60	1.05E-02	0.180
Vascular smooth muscle contraction	121	2.07	1.26E-02	0.206

Supplementary Table S18. scDrugHunter-identified top-ranked gene-drug interaction pairs in intestinal tuft positive cells (scDGS > 120)

Gene	Drug	Gene specificity rank	Gene correlation rank	Gene-drug interaction rank	SMultiXcan-based gene P value rank	scDGS
IL10RB	PEGINTERFERON LAMBDA-1A	8.33	8.53	9.94	9.86	166.83
ICAM1	ALICAFORSEN	7.67	9.20	9.79	8.98	158.25
ICAM1	BI-505	7.67	9.20	9.69	8.98	157.46
ICAM1	ENLIMOMAB PEGOL	7.67	9.20	9.57	8.98	156.42
CCR1	CCX354	8.94	6.78	9.98	9.87	156.03
CCR1	CHEMBL2205805	8.94	6.78	9.91	9.87	155.53
CCR1	BMS-817399	8.94	6.78	9.91	9.87	155.53
ICAM1	LIFITEGRAST	7.67	9.20	9.42	8.98	155.13
PDE4A	DROTAVERINE	8.82	7.86	9.27	9.32	155.01
PDE4A	ENPROFYLLINE	8.82	7.86	9.16	9.32	154.10
CCR1	AZD4818	8.94	6.78	9.71	9.87	153.98
ICAM1	NATALIZUMAB	7.67	9.20	9.13	8.98	152.67
ICAM1	HYALURONAN	7.67	9.20	9.13	8.98	152.67
PDE4A	APREMILAST	8.82	7.86	8.97	9.32	152.50
PDE4A	ROFLUMILAST	8.82	7.86	8.93	9.32	152.15
PDE4A	DYPHYLLINE	8.82	7.86	8.85	9.32	151.45
PDE4A	AROFYLLINE	8.82	7.86	8.78	9.32	150.87
PDE4A	OGLEMILAST	8.82	7.86	8.78	9.32	150.87
PDE4A	CHEMBL1229585	8.82	7.86	8.78	9.32	150.87
PDE4A	LOTAMILAST	8.82	7.86	8.78	9.32	150.87
TNFRSF4	IVUXOLIMAB	8.10	8.39	9.87	8.41	150.69
TNFRSF4	VONLEROLIZUMAB	8.10	8.39	9.87	8.41	150.69
TNFRSF4	TAVOLIMAB	8.10	8.39	9.87	8.41	150.69
CCR1	TERPYRIDINE	8.94	6.78	9.23	9.87	150.15
PDE4A	CRISABOROLE	8.82	7.86	8.64	9.32	149.72
PDE4A	PICLAMILAST	8.82	7.86	8.60	9.32	149.40
PDE4A	OXTRIPHYLLINE	8.82	7.86	8.57	9.32	149.17
PDE4A	REVAMILAST	8.82	7.86	8.51	9.32	148.67
PDE4A	MK-0873	8.82	7.86	8.51	9.32	148.67
PDE4A	LIRIMILAST	8.82	7.86	8.51	9.32	148.67
PDE4A	CHEMBL74078	8.82	7.86	8.51	9.32	148.67
PDE4A	IBUDILAST	8.82	7.86	8.34	9.32	147.24
ICAM1	NAFAMOSTAT	7.67	9.20	8.45	8.98	146.95
PDE4A	TOFISOPAM	8.82	7.86	8.11	9.32	145.34
PDE4A	MILRINONE	8.82	7.86	8.11	9.32	145.34
TYK2	TOFACITINIB	7.41	8.61	8.48	9.63	145.05
SENP7	CHEMBL378903	6.69	9.06	8.63	9.78	145.01
TYK2	BREPOCITINIB	7.41	8.61	8.40	9.63	144.38
PDE4A	TOFIMILAST	8.82	7.86	7.96	9.32	144.04
PDE4A	ROLIPRAM	8.82	7.86	7.96	9.32	144.04
PDE4A	DENBUFYLLINE	8.82	7.86	7.96	9.32	144.04
PDE4A	HT-0712	8.82	7.86	7.96	9.32	144.04
PDE4A	GSK-356278	8.82	7.86	7.96	9.32	144.04

PDE4A	PROPENTOFYLLINE	8.82	7.86	7.96	9.32	144.04
PDE4A	ILOPROST	8.82	7.86	7.96	9.32	144.04
P4HA2	PROLINE	6.16	9.97	9.63	8.19	143.69
DBP	ALBUMIN HUMAN	8.13	7.53	9.60	8.71	143.42
TYK2	PEFICITINIB	7.41	8.61	8.23	9.63	143.08
TYK2	CHEMBL21156	7.41	8.61	8.23	9.63	143.08
CLK3	CHEMBL1236620	6.38	9.09	9.71	8.70	142.35
PDE4A	THEOPHYLLINE	8.82	7.86	7.75	9.32	142.31
BGLAP	PHYTONADIONE	8.25	8.34	9.54	7.62	142.27
ICAM1	HYALURONATE SODIUM	7.67	9.20	7.89	8.98	142.26
SENP7	XANTHOANGELOL B	6.69	9.06	8.27	9.78	142.20
PLEKHA4	CHEMBL23552	8.02	6.60	9.90	9.47	141.46
PDE4A	CDC-801	8.82	7.86	7.58	9.32	140.91
PDE4A	CDP840	8.82	7.86	7.58	9.32	140.91
PDE4A	TADALAFIL	8.82	7.86	7.58	9.32	140.91
SENP7	CHEMBL546865	6.69	9.06	8.09	9.78	140.80
SENP7	CHEMBL584668	6.69	9.06	8.09	9.78	140.80
SENP7	DIHYDROXANTHOHUMOL	6.69	9.06	7.99	9.78	139.97
SENP7	CHEMBL546170	6.69	9.06	7.99	9.78	139.97
CLK3	CHEMBL261720	6.38	9.09	9.39	8.70	139.86
TYK2	TOFACITINIB CITRATE	7.41	8.61	7.75	9.63	139.20
TYK2	OCLACITINIB	7.41	8.61	7.75	9.63	139.20
TYK2	SOLCITINIB	7.41	8.61	7.75	9.63	139.20
TYK2	BMS-911543	7.41	8.61	7.75	9.63	139.20
TYK2	DELGOCITINIB	7.41	8.61	7.75	9.63	139.20
TYK2	NS-018	7.41	8.61	7.75	9.63	139.20
TYK2	FILGOTINIB	7.41	8.61	7.75	9.63	139.20
CLK3	LEUCETTAMINE B	6.38	9.09	9.29	8.70	139.10
SENP7	CHEMBL412603	6.69	9.06	7.81	9.78	138.58
SENP7	CHEMBL484663	6.69	9.06	7.81	9.78	138.58
TYK2	CERDULATINIB	7.41	8.61	7.62	9.63	138.17
BGLAP	PARICALCITOL	8.25	8.34	9.00	7.62	137.81
SENP7	CHEMBL1493528	6.69	9.06	7.65	9.78	137.32
SENP7	CHEMBL602561	6.69	9.06	7.65	9.78	137.32
SENP7	CHEMBL600313	6.69	9.06	7.65	9.78	137.32
PDE4A	FLAVOXATE HYDROCHLORIDE	8.82	7.86	6.96	9.32	135.75
TYK2	FEDRATINIB	7.41	8.61	7.30	9.63	135.62
TYK2	UPADACITINIB	7.41	8.61	7.30	9.63	135.62
BGLAP	CHEMBL38397	8.25	8.34	8.72	7.62	135.50
SENP7	CHEMBL576208	6.69	9.06	7.41	9.78	135.40
PDE4A	PENTOXIFYLLINE	8.82	7.86	6.91	9.32	135.30
CLK3	HARMINE	6.38	9.09	8.80	8.70	135.24
SENP7	IPRIFLAVONE	6.69	9.06	7.34	9.78	134.86
IFNAR2	INTERFERON ALFA-N3	3.59	10.00	9.85	9.93	134.36

SENP7	CHEMBL1504679	6.69	9.06	7.24	9.78	134.10
PDE4A	THEOPHYLLINE SODIUM GLYCINATE	8.82	7.86	6.76	9.32	134.04
PDE4A	INAMRINONE	8.82	7.86	6.76	9.32	134.04
TYK2	BARICITINIB	7.41	8.61	7.07	9.63	133.74
PDE4A	AMINOPHYLLINE	8.82	7.86	6.71	9.32	133.64
PDE4A	DIPYRIDAMOLE	8.82	7.86	6.71	9.32	133.64
VIPR2	SECRETIN	9.16	6.19	9.68	7.71	133.47
BGLAP	GALLIUM NITRATE	8.25	8.34	8.47	7.62	133.40
IFNAR2	INTERFERON ALFA-2B	3.59	10.00	9.70	9.93	133.34
SENP7	CHEMBL428789	6.69	9.06	7.14	9.78	133.30
VIPR2	VASOACTIVE INTESTINAL PEPTIDE	9.16	6.19	9.57	7.71	132.66
BGLAP	ISOFLAVONE	8.25	8.34	8.35	7.62	132.43
SENP7	CHEMBL299853	6.69	9.06	7.00	9.78	132.16
TYK2	ZOTIRACICLIB	7.41	8.61	6.84	9.63	131.91
BGLAP	CALCITONIN	8.25	8.34	8.26	7.62	131.66
TYK2	RUXOLITINIB	7.41	8.61	6.71	9.63	130.88
TYK2	ALDESLEUKIN	7.41	8.61	6.62	9.63	130.13
TYK2	AT-9283	7.41	8.61	6.62	9.63	130.13
PDE4A	ETAZOLATE	8.82	7.86	6.26	9.32	129.91
PDE4A	AMLEXANOX	8.82	7.86	6.16	9.32	129.08
IFNAR2	PEGINTERFERON ALFA-2A	3.59	10.00	9.05	9.93	128.95
IFNAR2	NYLIDRIN	3.59	10.00	9.05	9.93	128.95
IFNAR2	INTERFERON ALFACON-1	3.59	10.00	9.05	9.93	128.95
IFNAR2	PEGINTERFERON BETA-1A	3.59	10.00	9.05	9.93	128.95
IFNAR2	INTERFERON BETA-1B	3.59	10.00	9.05	9.93	128.95
IFNAR2	ANIFROLUMAB	3.59	10.00	9.05	9.93	128.95
IFNAR2	ALBINTERFERON ALFA-2B	3.59	10.00	9.05	9.93	128.95
IFNAR2	PEGINTERFERON ALFA-2B	3.59	10.00	9.05	9.93	128.95
IFNAR2	INTERFERON BETA-1A	3.59	10.00	9.05	9.93	128.95
IFNAR2	INTERFERON ALFA-2A	3.59	10.00	9.05	9.93	128.95
BGLAP	CLODRONIC ACID	8.25	8.34	7.69	7.62	126.95
IFNAR2	SIFALIMUMAB	3.59	10.00	8.76	9.93	126.93
BGLAP	ANDROSTANOLONE	8.25	8.34	7.58	7.62	126.05
TYK2	JNJ-7706621	7.41	8.61	6.08	9.63	125.80
JAK1	RUXOLITINIB	8.54	6.35	8.92	7.90	125.23
BGLAP	MITOMYCIN	8.25	8.34	7.47	7.62	125.16
BGLAP	COUMARIN	8.25	8.34	7.47	7.62	125.16
JAK1	ITACITINIB	8.54	6.35	8.86	7.90	124.76
JAK1	GLPG-0555	8.54	6.35	8.86	7.90	124.76
JAK1	INCB-047986	8.54	6.35	8.86	7.90	124.76

BGLAP	EXEMESTANE	8.25	8.34	7.34	7.62	124.06
JAK1	UPADACITINIB	8.54	6.35	8.75	7.90	123.93
JAK1	PEFICITINIB	8.54	6.35	8.71	7.90	123.62
THRA	LIOTRIX	8.72	8.15	9.89	4.75	123.51
BGLAP	ETIDRONIC ACID	8.25	8.34	7.27	7.62	123.49
JAK1	BARICITINIB	8.54	6.35	8.65	7.90	123.22
JAK1	FILGOTINIB	8.54	6.35	8.65	7.90	123.22
JAK1	SOLCITINIB	8.54	6.35	8.65	7.90	123.22
BGLAP	REGRAMOSTIM	8.25	8.34	7.21	7.62	123.01
JAK1	ABROCITINIB	8.54	6.35	8.62	7.90	122.95
BGLAP	METHIMAZOLE	8.25	8.34	7.11	7.62	122.14
BGLAP	PROPRANOLOL	8.25	8.34	7.07	7.62	121.79
BGLAP	INFLIXIMAB	8.25	8.34	7.03	7.62	121.47
PTGFR	TRAVOPROST	9.13	9.22	9.98	3.25	121.44
THRA	DEXTROTHYROXINE	8.72	8.15	9.65	4.75	121.43
JAK1	BREPOCITINIB	8.54	6.35	8.40	7.90	121.30
JAK1	MOMELOTINIB	8.54	6.35	8.40	7.90	121.30
JAK1	FEDRATINIB	8.54	6.35	8.40	7.90	121.30
JAK1	TRICETAMIDE	8.54	6.35	8.40	7.90	121.30
JAK1	RUXOLITINIB PHOSPHATE	8.54	6.35	8.40	7.90	121.30
PTGFR	LATANOPROST	9.13	9.22	9.87	3.25	120.42
PTGFR	TAFLUPROST	9.13	9.22	9.84	3.25	120.17
JAK1	CHEMBL21156	8.54	6.35	8.23	7.90	120.09
PTGFR	BIMATOPROST	9.13	9.22	9.83	3.25	120.06

Supplementary Table S19. Significant pathways enriched by up-regulated genes in brain endothelial positive cells

Description	Gene size	Enrichment ratio	P Value	FDR
Pathways in cancer	524	2.78	5.21E-08	1.55E-05
Protein digestion and absorption	90	6.37	9.53E-08	1.55E-05
AGE-RAGE signaling pathway in diabetic complications	99	5.79	3.01E-07	2.68E-05
PI3K-Akt signaling pathway	354	3.11	3.29E-07	2.68E-05
Focal adhesion	199	3.99	4.93E-07	3.21E-05
ECM-receptor interaction	82	5.37	1.44E-05	7.84E-04
Relaxin signaling pathway	130	4.07	3.55E-05	1.29E-03
MAPK signaling pathway	295	2.84	3.55E-05	1.29E-03
TNF signaling pathway	110	4.41	3.60E-05	1.29E-03
Proteoglycans in cancer	198	3.34	3.95E-05	1.29E-03
Small cell lung cancer	92	4.31	2.22E-04	6.58E-03
Fluid shear stress and atherosclerosis	138	3.51	2.81E-04	7.64E-03
Prostate cancer	97	4.09	3.32E-04	8.32E-03
Complement and coagulation cascades	79	4.46	3.94E-04	9.17E-03
MicroRNAs in cancer	150	3.23	5.76E-04	1.25E-02
Human T-cell leukemia virus 1 infection	255	2.59	6.47E-04	1.32E-02
Malaria	49	5.40	7.77E-04	1.49E-02
Amphetamine addiction	68	4.54	8.26E-04	1.50E-02

Melanoma	72	4.28	1.16E-03	2.00E-02
Protein processing in endoplasmic reticulum	165	2.94	1.27E-03	2.07E-02
Pertussis	76	4.06	1.60E-03	2.49E-02
Human papillomavirus infection	339	2.21	1.70E-03	2.51E-02
EGFR tyrosine kinase inhibitor resistance	79	3.90	2.01E-03	2.84E-02
Osteoclast differentiation	128	3.10	2.43E-03	3.31E-02
Transcriptional misregulation in cancer	186	2.61	3.28E-03	4.11E-02
Renin secretion	65	4.07	3.41E-03	4.11E-02
Central carbon metabolism in cancer	65	4.07	3.41E-03	4.11E-02
Apoptosis	136	2.92	3.66E-03	4.14E-02
JAK-STAT signaling pathway	162	2.72	3.68E-03	4.14E-02
Toxoplasmosis	112	3.15	3.82E-03	4.15E-02
Signaling pathways regulating pluripotency of stem cells	139	2.85	4.23E-03	4.45E-02
Human cytomegalovirus infection	225	2.35	5.00E-03	4.98E-02
IL-17 signaling pathway	93	3.32	5.04E-03	4.98E-02

Supplementary Table S20. Significant GO-term (biological process) enriched by up-regulated genes in brain endothelial positive cells

Description	Gene size	Enrichment ratio	P Value	FDR
ossification	371	5.39	1.00E-16	1.00E-16
angiogenesis	487	4.62	1.00E-16	1.00E-16
extracellular structure organization	400	6.25	1.00E-16	1.00E-16
connective tissue development	265	5.47	8.75E-14	1.86E-11
epithelial cell proliferation	372	4.57	1.17E-13	1.99E-11
reproductive system development	428	3.97	6.31E-12	8.50E-10
response to oxygen levels	337	4.45	7.00E-12	8.50E-10
in utero embryonic development	345	4.35	1.27E-11	1.29E-09
negative regulation of immune system process	416	3.97	1.37E-11	1.29E-09
morphogenesis of an epithelium	480	3.65	3.37E-11	2.87E-09
morphogenesis of a branching structure	196	5.61	5.89E-11	4.55E-09
negative regulation of cellular component movement	301	4.49	7.00E-11	4.96E-09
negative regulation of intracellular signal transduction	495	3.54	7.90E-11	5.17E-09
embryonic organ development	423	3.78	9.79E-11	5.94E-09
negative regulation of locomotion	314	4.30	1.83E-10	1.04E-08
respiratory tube development	172	5.82	2.38E-10	1.26E-08
respiratory system development	193	5.44	2.92E-10	1.38E-08
positive regulation of cell motility	493	3.45	2.92E-10	1.38E-08
muscle tissue development	371	3.91	3.64E-10	1.63E-08
regulation of DNA-templated transcription in response to stress	78	8.98	3.89E-10	1.65E-08
tissue migration	283	4.42	5.06E-10	2.05E-08
muscle cell proliferation	182	5.50	6.57E-10	2.54E-08
gland development	434	3.57	8.07E-10	2.87E-08
collagen metabolic process	97	7.74	8.11E-10	2.87E-08
regulation of vasculature development	313	4.16	8.53E-10	2.90E-08

response to steroid hormone	388	3.74	1.04E-09	3.39E-08
regulation of hemopoiesis	389	3.73	1.10E-09	3.46E-08
aging	303	4.13	2.10E-09	6.38E-08
response to transforming growth factor beta	238	4.62	2.48E-09	7.26E-08
response to acid chemical	332	3.92	2.99E-09	8.20E-08
cell-substrate adhesion	332	3.92	2.99E-09	8.20E-08
response to fibroblast growth factor	142	5.99	3.49E-09	9.28E-08
regulation of apoptotic signaling pathway	385	3.64	3.76E-09	9.67E-08
fat cell differentiation	210	4.76	8.07E-09	2.02E-07
urogenital system development	326	3.84	9.32E-09	2.19E-07
ERK1 and ERK2 cascade	326	3.84	9.32E-09	2.19E-07
regulation of cellular response to growth factor stimulus	256	4.30	9.58E-09	2.19E-07
skeletal system morphogenesis	234	4.49	9.78E-09	2.19E-07
transmembrane receptor protein serine/threonine kinase signaling pathway	328	3.81	1.05E-08	2.30E-07
ameboidal-type cell migration	381	3.55	1.26E-08	2.67E-07
response to temperature stimulus	201	4.48	1.18E-07	2.09E-06
gastrulation	175	4.29	2.36E-06	3.04E-05
cardiocyte differentiation	141	4.61	5.02E-06	6.10E-05
cell fate commitment	249	3.42	1.11E-05	1.20E-04
cellular response to external stimulus	330	3.03	1.11E-05	1.20E-04
negative regulation of proteolysis	336	2.98	1.45E-05	1.54E-04
leukocyte cell-cell adhesion	319	2.82	7.77E-05	6.67E-04
negative regulation of transferase activity	266	3.01	9.25E-05	7.63E-04
coagulation	326	2.76	1.02E-04	8.27E-04
response to organophosphorus	138	3.99	1.03E-04	8.27E-04
negative regulation of nervous system development	297	2.86	1.03E-04	8.27E-04
sensory organ morphogenesis	250	3.00	1.57E-04	1.19E-03
response to toxic substance	499	2.31	1.79E-04	1.32E-03
regulation of developmental growth	314	2.71	2.01E-04	1.44E-03
response to ketone	189	3.18	4.26E-04	2.70E-03
body morphogenesis	50	6.00	4.60E-04	2.88E-03
response to interferon-beta	33	7.58	4.63E-04	2.88E-03
cardiac chamber development	164	3.36	4.63E-04	2.88E-03
response to tumor necrosis factor	251	2.79	5.41E-04	3.31E-03
regulation of chemotaxis	205	2.93	8.77E-04	4.97E-03
stem cell division	40	6.25	1.14E-03	6.28E-03
negative regulation of defense response	187	2.94	1.37E-03	7.41E-03
inositol lipid-mediated signaling	165	3.03	1.80E-03	9.48E-03
protein hydroxylation	27	7.41	1.91E-03	1.00E-02
coronary vasculature development	47	5.32	2.38E-03	1.21E-02
STAT cascade	148	3.04	2.94E-03	1.41E-02
response to endoplasmic reticulum stress	268	2.43	2.94E-03	1.41E-02
nerve development	77	3.90	4.38E-03	1.98E-02
protein trimerization	54	4.63	4.39E-03	1.98E-02
viral life cycle	285	2.28	4.94E-03	2.16E-02
response to interferon-alpha	20	7.50	7.01E-03	3.01E-02
regulation of protein catabolic process	370	2.03	7.68E-03	3.25E-02

hormone-mediated signaling pathway	237	2.32	8.27E-03	3.45E-02
axon development	490	1.84	9.83E-03	4.00E-02
receptor metabolic process	183	2.46	1.14E-02	4.54E-02

Supplementary Table S21. scDrugHunter-identified top-ranked gene-drug interaction pairs in brain endothelial positive cells (scDGS > 120)

Gene	Drug	Gene specificity rank	Gene correlation rank	Gene-drug interaction rank	SMultiXcan-based gene P value rank	scDGS
PLEKHA4	CHEMBL23552	9.40	9.41	9.88	9.27	180.17
LTF	CHEMBL609880	9.51	8.58	9.90	9.31	173.66
LTF	CHEMBL92636	9.51	8.58	9.90	9.31	173.66
ICAM1	ALICAFORSEN	9.25	9.38	9.75	8.80	172.75
ICAM1	BI-505	9.25	9.38	9.64	8.80	171.76
LTF	PARECOXIB	9.51	8.58	9.59	9.31	170.86
ICAM1	ENLIMOMAB PEGOL	9.25	9.38	9.50	8.80	170.47
LTF	NITRILOTRIACETIC ACID	9.51	8.58	9.53	9.31	170.35
P4HA2	PROLINE	9.57	9.73	9.57	8.03	169.80
LTF	TALACTOFERRIN ALFA	9.51	8.58	9.41	9.31	169.29
LTF	CHEMBL1230004	9.51	8.58	9.41	9.31	169.29
ICAM1	LIFITEGRAST	9.25	9.38	9.32	8.80	168.76
LTF	BACITRACIN	9.51	8.58	9.12	9.31	166.62
ICAM1	NATALIZUMAB	9.25	9.38	9.02	8.80	165.96
ICAM1	HYALURONAN	9.25	9.38	9.02	8.80	165.96
PDE4A	DROTAVERINE	8.74	9.20	9.16	9.14	164.09
PDE4A	ENPROFYLLINE	8.74	9.20	9.06	9.14	163.14
PDE4A	APREMILAST	8.74	9.20	8.86	9.14	161.39
PDE4A	ROFLUMILAST	8.74	9.20	8.82	9.14	161.02
PDE4A	DYPHYLLINE	8.74	9.20	8.71	9.14	160.02
IL10RB	PEGINTERFERON LAMBDA-1A	8.57	7.72	9.93	9.68	159.75
LTF	DODECANOATE	9.51	8.58	8.33	9.31	159.50
PDE4A	AROFYLLINE	8.74	9.20	8.64	9.14	159.41
PDE4A	OGLEMILAST	8.74	9.20	8.64	9.14	159.41
PDE4A	CHEMBL1229585	8.74	9.20	8.64	9.14	159.41
PDE4A	LOTAMILAST	8.74	9.20	8.64	9.14	159.41
ICAM1	NAFAMOSTAT	9.25	9.38	8.30	8.80	159.31
CYP3A43	GINKGO	9.97	8.82	9.57	7.36	159.04
CSF3	BENEGRASTIM	9.14	7.86	9.75	8.94	158.70
CSF3	EFLAPEGRASTIM	9.14	7.86	9.75	8.94	158.70
CCR9	VERCIRNON	9.11	6.73	10.00	10.00	158.39
CCR9	MLN3126	9.11	6.73	9.98	10.00	158.25
PDE4A	CRISABOROLE	8.74	9.20	8.50	9.14	158.14
LTF	NIMESULIDE	9.51	8.58	8.18	9.31	158.11
PDE4A	PICLAMILAST	8.74	9.20	8.46	9.14	157.80
PDE4A	OXTRIPHYLLINE	8.74	9.20	8.43	9.14	157.51
PDE4A	REVAMILAST	8.74	9.20	8.37	9.14	156.98

PDE4A	MK-0873	8.74	9.20	8.37	9.14	156.98
PDE4A	LIRIMILAST	8.74	9.20	8.37	9.14	156.98
PDE4A	CHEMBL74078	8.74	9.20	8.37	9.14	156.98
CSF3	MUPLESTIM	9.14	7.86	9.50	8.94	156.62
IFNAR2	INTERFERON ALFA-N3	5.79	10.00	9.82	9.92	155.81
PDE4A	IBUDILAST	8.74	9.20	8.20	9.14	155.47
CYP3A43	CHLORZOXAZON E	9.97	8.82	9.18	7.36	155.43
IFNAR2	INTERFERON ALFA-2B	5.79	10.00	9.65	9.92	154.46
ICAM1	HYALURONATE SODIUM	9.25	9.38	7.76	8.80	154.28
LTF	RESERPINE	9.51	8.58	7.69	9.31	153.68
PDE4A	TOFISOPAM	8.74	9.20	7.98	9.14	153.46
PDE4A	MILRINONE	8.74	9.20	7.98	9.14	153.46
CSF3	TALABOSTAT	9.14	7.86	9.12	8.94	153.37
CYP3A43	TICAGRELOR	9.97	8.82	8.87	7.36	152.50
PDE4A	TOFIMILAST	8.74	9.20	7.83	9.14	152.14
PDE4A	ROLIPRAM	8.74	9.20	7.83	9.14	152.14
PDE4A	DENBUFYLLINE	8.74	9.20	7.83	9.14	152.14
PDE4A	HT-0712	8.74	9.20	7.83	9.14	152.14
PDE4A	GSK-356278	8.74	9.20	7.83	9.14	152.14
PDE4A	PROPENTOFYLLI NE	8.74	9.20	7.83	9.14	152.14
PDE4A	ILOPROST	8.74	9.20	7.83	9.14	152.14
CYP3A43	COBICISTAT	9.97	8.82	8.70	7.36	150.89
PDE4A	THEOPHYLLINE	8.74	9.20	7.63	9.14	150.31
IFNAR2	PEGINTERFERON ALFA-2A	5.79	10.00	8.95	9.92	148.94
IFNAR2	NYLIDRIN	5.79	10.00	8.95	9.92	148.94
IFNAR2	INTERFERON ALFACON-1	5.79	10.00	8.95	9.92	148.94
IFNAR2	PEGINTERFERON BETA-1A	5.79	10.00	8.95	9.92	148.94
IFNAR2	INTERFERON BETA-1B	5.79	10.00	8.95	9.92	148.94
IFNAR2	ANIFROLUMAB	5.79	10.00	8.95	9.92	148.94
IFNAR2	ALBINTERFERON ALFA-2B	5.79	10.00	8.95	9.92	148.94
IFNAR2	PEGINTERFERON ALFA-2B	5.79	10.00	8.95	9.92	148.94
IFNAR2	INTERFERON BETA-1A	5.79	10.00	8.95	9.92	148.94
IFNAR2	INTERFERON ALFA-2A	5.79	10.00	8.95	9.92	148.94
PDE4A	CDC-801	8.74	9.20	7.46	9.14	148.83
PDE4A	CDP840	8.74	9.20	7.46	9.14	148.83
PDE4A	TADALAFIL	8.74	9.20	7.46	9.14	148.83
IFNAR2	SIFALIMUMAB	5.79	10.00	8.62	9.92	146.36
JAK1	RUXOLITINIB	8.45	9.22	8.81	7.75	146.35
JAK1	ITACITINIB	8.45	9.22	8.75	7.75	145.80
JAK1	GLPG-0555	8.45	9.22	8.75	7.75	145.80

JAK1	INCB-047986	8.45	9.22	8.75	7.75	145.80
VIPR2	SECRETIN	8.12	8.85	9.62	7.54	145.54
JAK1	UPADACITINIB	8.45	9.22	8.61	7.75	144.57
VIPR2	VASOACTIVE INTESTINAL PEPTIDE	8.12	8.85	9.50	7.54	144.57
JAK1	PEFICITINIB	8.45	9.22	8.57	7.75	144.21
CYP3A43	KETOCONAZOLE	9.97	8.82	7.99	7.36	144.21
PSORS1C1	ALLOPURINOL	9.45	7.02	8.63	8.83	143.79
JAK1	BARICITINIB	8.45	9.22	8.52	7.75	143.71
JAK1	FILGOTINIB	8.45	9.22	8.52	7.75	143.71
JAK1	SOLCITINIB	8.45	9.22	8.52	7.75	143.71
JAK1	ABROCITINIB	8.45	9.22	8.48	7.75	143.38
PDE4A	FLAVOXATE HYDROCHLORIDE	8.74	9.20	6.84	9.14	143.27
PDE4A	PENTOXIFYLLINE	8.74	9.20	6.78	9.14	142.74
CYP3A43	MELATONIN	9.97	8.82	7.79	7.36	142.35
LTA	PATECLIZUMAB	7.37	8.23	9.67	8.56	142.20
PDE4A	THEOPHYLLINE SODIUM GLYCINATE	8.74	9.20	6.63	9.14	141.42
PDE4A	INAMRINONE	8.74	9.20	6.63	9.14	141.42
JAK1	BREPOCITINIB	8.45	9.22	8.25	7.75	141.39
JAK1	MOMELOTINIB	8.45	9.22	8.25	7.75	141.39
JAK1	FEDRATINIB	8.45	9.22	8.25	7.75	141.39
JAK1	TRICETAMIDE	8.45	9.22	8.25	7.75	141.39
JAK1	RUXOLITINIB PHOSPHATE	8.45	9.22	8.25	7.75	141.39
PDE4A	AMINOPHYLLINE	8.74	9.20	6.59	9.14	141.00
PDE4A	DIPYRIDAMOLE	8.74	9.20	6.59	9.14	141.00
LTA	BAMINERCEPT	7.37	8.23	9.41	8.56	140.22
JAK1	CHEMBL21156	8.45	9.22	8.10	7.75	140.06
JAK1	TOFACITINIB	8.45	9.22	7.93	7.75	138.50
JAK1	XL-019	8.45	9.22	7.93	7.75	138.50
JAK1	AZD-1480	8.45	9.22	7.93	7.75	138.50
JAK1	CERDULATINIB	8.45	9.22	7.93	7.75	138.50
JAK1	DECERNOTINIB	8.45	9.22	7.93	7.75	138.50
CYP3A43	OLANZAPINE	9.97	8.82	7.35	7.36	138.25
PSORS1C1	ETANERCEPT	9.45	7.02	7.93	8.83	138.04
LTA	ETANERCEPT	7.37	8.23	9.07	8.56	137.58
PDE4A	ETAZOLATE	8.74	9.20	6.15	9.14	137.06
TYK2	TOFACITINIB	8.13	7.24	8.34	9.46	136.81
PDE4A	AMLEXANOX	8.74	9.20	6.05	9.14	136.18
TYK2	BREPOCITINIB	8.13	7.24	8.25	9.46	136.17
JAK1	OCLACITINIB	8.45	9.22	7.63	7.75	135.84
JAK1	NS-018	8.45	9.22	7.63	7.75	135.84
JAK1	BMS-911543	8.45	9.22	7.63	7.75	135.84
JAK1	TOFACITINIB CITRATE	8.45	9.22	7.63	7.75	135.84

JAK1	DELGOCITINIB	8.45	9.22	7.63	7.75	135.84
TYK2	PEFICITINIB	8.13	7.24	8.10	9.46	135.01
TYK2	CHEMBL21156	8.13	7.24	8.10	9.46	135.01
PSORS1C1	CARBOPLATIN	9.45	7.02	7.40	8.83	133.74
PSORS1C1	GEMCITABINE	9.45	7.02	7.35	8.83	133.32
JAK1	PACRITINIB	8.45	9.22	7.32	7.75	133.18
JAK1	PEMBROLIZUMAB	8.45	9.22	7.26	7.75	132.58
LTA	ABACAVIR	7.37	8.23	8.40	8.56	132.28
TYK2	TOFACITINIB CITRATE	8.13	7.24	7.63	9.46	131.34
TYK2	OCLACITINIB	8.13	7.24	7.63	9.46	131.34
TYK2	SOLCITINIB	8.13	7.24	7.63	9.46	131.34
TYK2	BMS-911543	8.13	7.24	7.63	9.46	131.34
TYK2	DELGOCITINIB	8.13	7.24	7.63	9.46	131.34
TYK2	NS-018	8.13	7.24	7.63	9.46	131.34
TYK2	FILGOTINIB	8.13	7.24	7.63	9.46	131.34
CPOX	GIVOSIRAN	9.01	9.09	9.96	4.50	130.84
TYK2	CERDULATINIB	8.13	7.24	7.50	9.46	130.37
JAK1	ENZASTAURIN	8.45	9.22	6.95	7.75	129.85
TYK2	FEDRATINIB	8.13	7.24	7.18	9.46	127.92
TYK2	UPADACITINIB	8.13	7.24	7.18	9.46	127.92
JAK1	ZOTIRACICLIB	8.45	9.22	6.71	7.75	127.79
LTA	CARBAMAZEPINE	7.37	8.23	7.76	8.56	127.36
PTGFR	TRAVOPROST	9.74	9.54	9.97	3.19	126.90
TYK2	BARICITINIB	8.13	7.24	6.95	9.46	126.13
PTGFR	LATANOPROST	9.74	9.54	9.84	3.19	125.62
PTGFR	TAFLUPROST	9.74	9.54	9.81	3.19	125.37
PTGFR	BIMATOPROST	9.74	9.54	9.79	3.19	125.14
TYK2	ZOTIRACICLIB	8.13	7.24	6.71	9.46	124.34
SPARC	CALCIUM PHOSPHATE, TRIBASIC	9.51	9.79	8.25	4.59	123.89
SPARC	CALCIUM CITRATE	9.51	9.79	8.25	4.59	123.89
TYK2	RUXOLITINIB	8.13	7.24	6.59	9.46	123.36
TYK2	ALDESLEUKIN	8.13	7.24	6.50	9.46	122.66
TYK2	AT-9283	8.13	7.24	6.50	9.46	122.66
PTGFR	LATANOPROSTENE BUNOD	9.74	9.54	9.47	3.19	122.10
JAK1	MYRICETIN	8.45	9.22	6.05	7.75	121.92
